# Characterization and expression profiling of serine protease inhibitors in the diamondback moth, *Plutella xylostella* (Lepidoptera: Plutellidae)

**DOI:** 10.1186/s12864-017-3583-z

**Published:** 2017-02-14

**Authors:** Hailan Lin, Xijian Lin, Jiwei Zhu, Xiao-Qiang Yu, Xiaofeng Xia, Fengluan Yao, Guang Yang, Minsheng You

**Affiliations:** 1 State Key Laboratory of Ecological Pest Control for Fujian and Taiwan Crops, and College of Life Science, Fujian Agriculture and Forestry University, Fuzhou, 350002 China; 2 Institute of Applied Ecology and Research Centre for Biodiversity and Eco-Safety, Fujian Agriculture and Forestry University, Fuzhou, 350002 China; 3 Fujian-Taiwan Joint Innovation Centre for Ecological Control of Crop Pests, Fujian Agriculture and Forestry University, Fuzhou, 350002 China; 4 Key Laboratory of Integrated Pest Management of Fujian and Taiwan, China Ministry of Agriculture, Fuzhou, 350002 China; 5 School of Biological Sciences, University of Missouri, Kansas City, MO 64110-2499 USA; 6 Institute of Plant Protection, Fujian Academy of Agricultural Sciences, Fuzhou, 350013 China

**Keywords:** Serpin, Canonical inhibitor, Expression pattern, Phylogenetic analysis, Lepidoptera

## Abstract

**Background:**

Serine protease inhibitors (SPIs) have been found in all living organisms and play significant roles in digestion, development and innate immunity. In this study, we present a genome-wide identification and expression profiling of SPI genes in the diamondback moth, *Plutella xylostella* (L.), a major pest of cruciferous crops with global distribution and broad resistance to different types of insecticides.

**Results:**

A total of 61 potential SPI genes were identified in the *P. xylostella* genome, and these SPIs were classified into serpins, canonical inhibitors, and alpha-2-macroglobulins based on their modes of action. Sequence alignments showed that amino acid residues in the hinge region of known inhibitory serpins from other insect species were conserved in most *P. xylostella* serpins, suggesting that these *P. xylostella* serpins may be functionally active. Phylogenetic analysis confirmed that *P. xylostella* inhibitory serpins were clustered with known inhibitory serpins from six other insect species. More interestingly, nine serpins were highly similar to the orthologues in *Manduca sexta* which have been demonstrated to participate in regulating the prophenoloxidase activation cascade, an important innate immune response in insects. Of the 61 *P.xylostella* SPI genes, 33 were canonical SPIs containing seven types of inhibitor domains, including Kunitz, Kazal, TIL, amfpi, Antistasin, WAP and Pacifastin. Moreover, some SPIs contained additional non-inhibitor domains, including spondin_N, reeler, and other modules, which may be involved in protein-protein interactions. Gene expression profiling showed gene-differential, stage- and sex-specific expression patterns of SPIs, suggesting that SPIs may be involved in multiple physiological processes in *P. xylostella*.

**Conclusions:**

This is the most comprehensive investigation so far on SPI genes in *P. xylostella*. The characterized features and expression patterns of *P. xylostell*a SPIs indicate that the SPI family genes may be involved in innate immunity of this species. Our findings provide valuable information for uncovering further biological roles of SPI genes in *P. xylostella*.

**Electronic supplementary material:**

The online version of this article (doi:10.1186/s12864-017-3583-z) contains supplementary material, which is available to authorized users.

## Background

Serine proteases are ubiquitous enzymes in almost all organisms, from bacteria to mammals [1], and they are known to play significant roles in a wide range of biological processes [2–5]. Besides their crucial physiological roles, proteases carry out an unlimited number of hydrolytic reactions to break down proteins [6], and such proteolytic activity can be potentially hazardous in living systems. Consequently, protease activity must be strictly and precisely controlled [7–9]. There are several distinct mechanisms for the regulation of excessive activity of proteases. The most effective and direct mechanism is to inactivate proteases by protease inhibitors [7, 8]. Serine protease inhibitors (SPIs) have been widely studied with documented roles in digestion, metamorphosis and development, as well as in immune responses [10–16]. SPIs can be divided into three distinct types: serpins, canonical ﻿﻿inhibitors﻿, and non-canonical inhibitors, based on their mechanisms of action [7, 9]. Interaction between a serpin and its target protease is similar to substrate binding, and cleavage of a single peptide bond in the binding loop results in conformational change of serpin [17–19]. The canonical inhibitors bind to the enzymes by an exposed convex binding loop, docking to the active site of the proteases that leads to inactivation of proteases [7, 9]. The non-canonical inhibitors interact with target proteases through their N-terminal segments, which are secondary interactions outside the active site to significantly enhance the affinity, velocity and specificity of recognition [7, 9].

Serpins are found in nearly all organisms, and are a superfamily of proteins with three β-sheets and seven to nine α-helices folding into a conserved tertiary structure with a reactive center loop (RCL) [19]. The RCL is located in the carboxyl terminus, and exposed at the surface of a serpin, acting as a ‘bait’ for target proteases [20, 21]. After proteases cleave the RCL at the scissile bond, serpins undergo conformational change, resulting in inactivation of target proteases [15]. Previous studies have illustrated that most serpins are inhibitors of serine proteases [15, 20, 21], while some serpins become inhibitors of caspases [22] and papain-like cysteine proteases [23, 24]. Meanwhile, some other serpins are non-inhibitors, achieving a number of other biological tasks, such as tumor suppressors, hormone transporters and molecular chaperones [25–27].

In contrast to serpins, canonical inhibitors are the largest group of protein inhibitors and they are usually small proteins with 14 ~ 200 amino acids (aa) [7]. Canonical inhibitors can be classified into different families based on sequence homology, position of active center and disulfide bond, including Kazal, Kunitz and Antistasin families [7, 9]. Alpha-2-macroglobulin (α2M) is a large protein belonging to the thiol ester superfamily [28], which is known for its ability to inhibit a broad spectrum of proteases, including serine proteases, papain, aspartic proteases and metalloproteases [29, 30]. The inhibitory mechanism of α2M is to physically enfold target proteases by a macromolecular cage, forming a complex of α2M-protease to prevent protease from accessing protein substrates [31, 32].

Whole-genome investigations on the serpin family have been carried out in a number of insect species, including *Anopheles gambiae* and *Aedes aegypti* (Diptera: Culicidae) [20, 33], 12 *Drosophila species* (Diptera: Drosophilidae) [34], *Apis mellifera* (Hymenoptera: Apidae) [3], *Tribolium castaneum* (Coleoptera: Tenebrionidae) [35], and *Bombyx mori* (Lepidoptera: Bombycidae) [36]. Seven *Manduca sexta* (Lepidoptera: Sphingidae) serpins have been characterized through biochemical studies [13, 37–42]. Previous studies reveal that most serpins function as serine protease inhibitors and play significant roles in regulation of innate immunity by controlling proteolytic pathways. The genome-wide analysis of canonical inhibitors has been performed in *B. mori* [43], and some individual canonical inhibitors have been characterized in other insect species. For example, two Kunitz-type inhibitors from the hemolymph of *M. sexta* functioned as inhibitors of serine proteases, including trypsin, chymotrypsin and plasmin [44]. A Kazal-type inhibitor in the midgut of *Rhodnius prolixus* (Hemiptera: Reduviidae) may be involved in the interaction between microbiota and *Trypanosoma cruzi* [45]; two non-classical Kazal-type serine proteinase inhibitors (PpKzl1 and PpKzl2) were identified in *Phlebotomus papatasi* (Diptera: Psychodidae), and PpKzl2 has been described as an active serine proteinase inhibitor that is possibly involved in regulating digestive enzymes in the midgut [10]. However, only three *P. xylostella* SPIs (serpins 2, 4 and 5) have been reported. By proteomic profiling and cloning, it has been shown that the expression of *P. xylostella* serpin 2 gene was reduced to 50% of the control during early parasitism (*Cotesia plutellae*) [46]; and its expression was also significantly affected by destruxin A (the mycotoxin produced by *Metarhizium anisopliae*) [47]. Knockdown of serpins 2, 4 and 5 by RNAi can induce expression of cecropins, increase phenoloxidase (PO) activity and body melanization in larvae, as well as mortality of *P. xylostella* larvae [48].

In this study, *P. xylostella* SPIs were identified and characterized based on the *P. xylostella* genome [49]. Our findings provide a foundation for further studies on the biological functions of SPIs in *P. xylostella*, which may be used in the development of genomic strategies for pest management.

## Results

### Identification of *P. xylostella* SPIs

Amino acid sequences of SPIs from *B. mori*, *M. sexta* (Lepidoptera), *D* *. melanogaster*, *A. gambiae* (Diptera), *T. castaneum* (Coleoptera) and *A. mellifera* (Hymenoptea) were used to search *P. xylostella* genomic sequences. A total of 61 putative SPI genes were identified in *P. xylostella* (Table 1 and Additional file 1: Table S1), and the deduced amino acid sequences were provided in Additional file 1: Table S2. The 61 putative SPI genes were classified into three types: serpins, canonical SPIs, and α2Ms (Table 1) based on their mechanisms of action.

**Table 1:** Serine protease inhibitor (SPI) domains in *Plutella xylostella*

Name	SPI domain	Number of the SPI domains	Category
PxSPI1 ~ PxSPI25	Serpin	1	serpin
PxSPI26	TIL	13	canonical SPIs
PxSPI27	TIL	6	
PxSPI28	TIL	17	
PxSPI29	TIL	3	
PxSPI30	TIL	4	
PxSPI31 ~ PxSPI33	TIL	3	
PxSPI34	TIL	1	
PxSPI35	Kunitz	1	
PxSPI36 ~ PxSPI37	Kunitz	2	
PxSPI38	Kunitz	3	
PxSPI39	Kunitz	2	
PxSPI40 ~ PxSPI41	Kunitz	1	
PxSPI42	Kunitz/WAP	10/1	
PxSPI43	WAP	1	
PxSPI44	WAP/Antistasin/Kunizt	3/2/1	
PxSPI45	Kazal	4	
PxSPI46	Kazal	6	
PxSPI47	Kazal	1	
PxSPI48	Kazal	4	
PxSPI49 ~ PxSPI50	Kazal	1	
PxSPI51	Kazal	3	
PxSPI52	Kazal	5	
PxSPI53	Kazal	1	
PxSPI54	Kazal	3	
PxSPI55	Kazal	2	
PxSPI56	Kazal	1	
PxSPI57	amfpi	1	
PxSPI58	Pacifastin	4	
PxSPI59 ~ PxSPI61	α2-macroglobulin	1	α2-macroglobulin

### Serpins

**Fig. 1:**
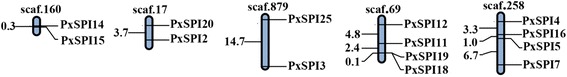
Scaffold localization of the serpin genes in *P. xylostella*. Gene names and the distance of two adjacent genes (kilobases, kb) are shown on the right and left of the bar, respectively. Scaffold numbers are indicated on the top of each bar

In this study, 25 serpins were identified in *P. xylostella* and named PxSPI1-PxSPI25. This number is fewer than 34 serpins in *B. mori* [21], 31 in *T. castaneum* [35] and 29 in *D. melanogaster* [15], but greater than 18 serpins in *A. gambiae* [20] and seven in *A. mellifera* [3]. The 25 serpin genes were spread across 16 different scaffolds (Additional file 1: Table S1). Of the 25 serpins, 14 were grouped in five clusters, forming three 2-gene clusters on scaffold 17, 160 and 879 and two 4-gene clusters on scaffold 69 and 258 (Fig. 1). Eleven of the 25 serpins were predicted to be secreted proteins based on the putative secretion signal peptides; 11 lacked putative signal peptides and were predicted to be intracellular proteins; whereas two (PxSPI10 and PxSPI20) were incomplete at the amino-terminus, one (PxSPI25) was incomplete at the carboxyl terminus (Additional file 1: Table S1).

**Fig. 2:**
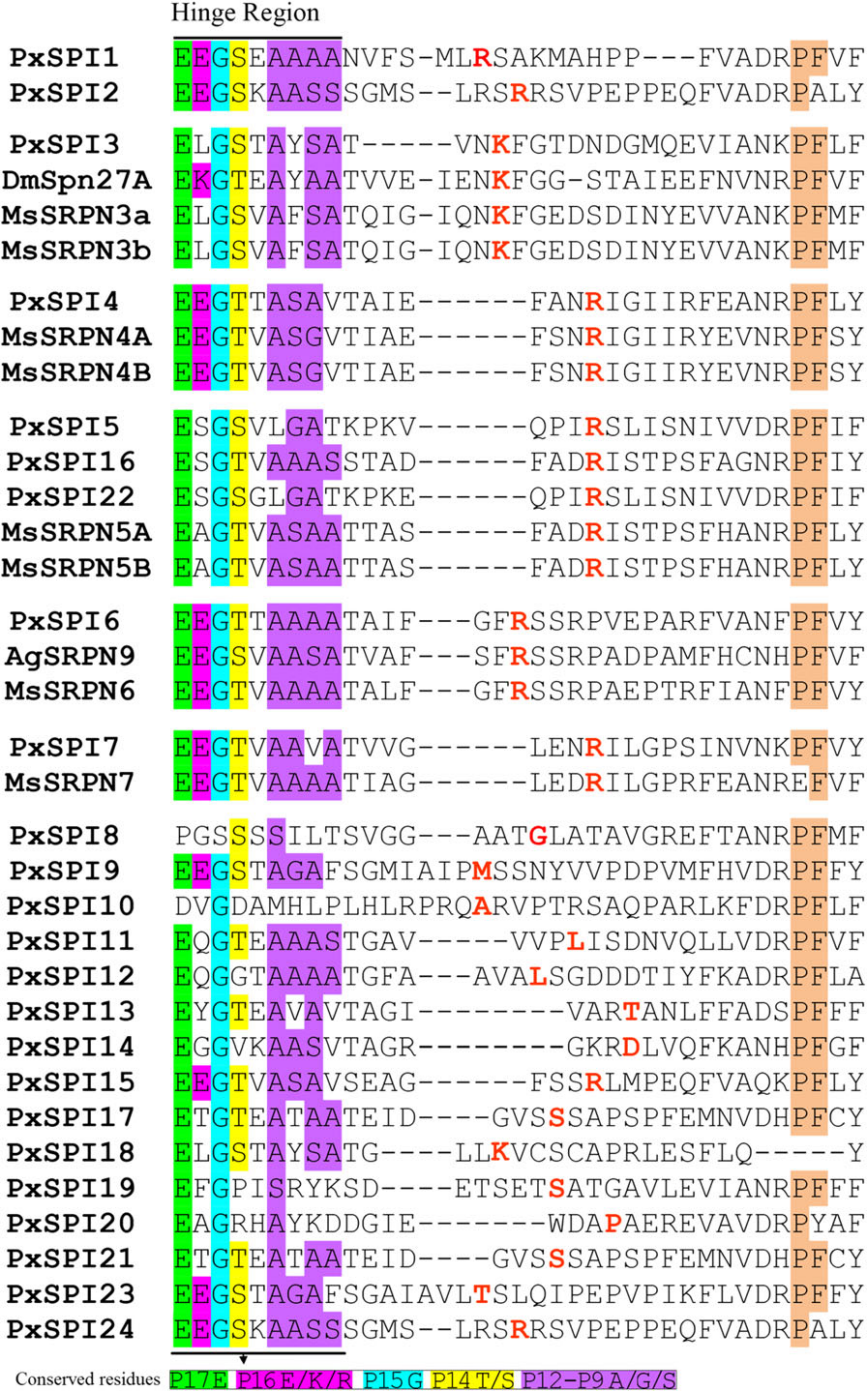
Multiple sequence alignment of the hinge and reactive center loop regions of *P. xylostella* serpins. The hinge and RCL regions of *P. xylostella* serpins were aligned with those of serpins with known functions from *D. melanogaster* (Dm), *M. sexta* (Ms) and *A. gambiae* (Ag) by ClustalX 2.0 program with default parameters. Predicted P1 residues are highlighted in red. *P. xylostella* serpins are presented in numerical order and grouped with homologous serpins from other species as determined by phylogenetic analysis (Fig. 3)

Serpins are metastable proteins that experience conformational changes as they inhibit proteases [19]. Several structural regions (the hinge, breach, shutter and gate regions) are necessary for conformational changes of serpins. The hinge is an essential region where the peptide chain bends to permit RCL insertion, and the breach and shutter regions must open to allow RCL insertion [50, 51]. A region known as the gate, which participates in a structural transition (latency) during RCL insertion, occurs in the absence of RCL cleavage [52]. To investigate the important structural regions of *P. xylostella* serpins, we aligned *P. xylostella* serpins with known inhibitory serpins from *M. sexta* (MsSRPN3-7), *D. melanogaster* (DmSpn27A) and *A. gambiae* (AgSRPN9). The result revealed that most residues in these structural regions (the breach, shutter and gate) in known inhibitory serpins, were conserved in most *P. xylostella* serpins (Additional file 2: Figure S1). The alignment also showed that most residues in the hinge region in the known inhibitory serpins were conserved in most *P. xylostella* serpins, except for PxSPI8, PxSPI10, PxSPI19, PxSPI20 and PxSPI25 (PxSPI25 was incomplete at the carboxyl terminus) (Fig. 2). It has been documented that serpins may inhibit trypsin-like enzymes with either an Arg (R) or Lys (K) residue at the P1 position, inhibit chymotrypsin-like enzymes with P1 residue of Phe (F), Tyr (Y), Leu (L) or Ile (I), and inhibit elastase-like enzymes with P1 residue of Ala (A) or Val (V) [21, 53]. Meanwhile, prediction of the proteolytic cleavage site of serpins showed that PxSPI1-7, 15, 16, 18, 22 and 24 had an R or K residue at the P1 position, indicating that they may inhibit trypsin-like enzymes, whereas PxSPI11 and PxSPI12 containing Leu (L) at the P1 position may inhibit chymotrypsin-like enzymes, and PxSPI10 with Ala at the P1 position may inhibit elastase-like enzymes (Fig. 2).

**Fig. 3:**
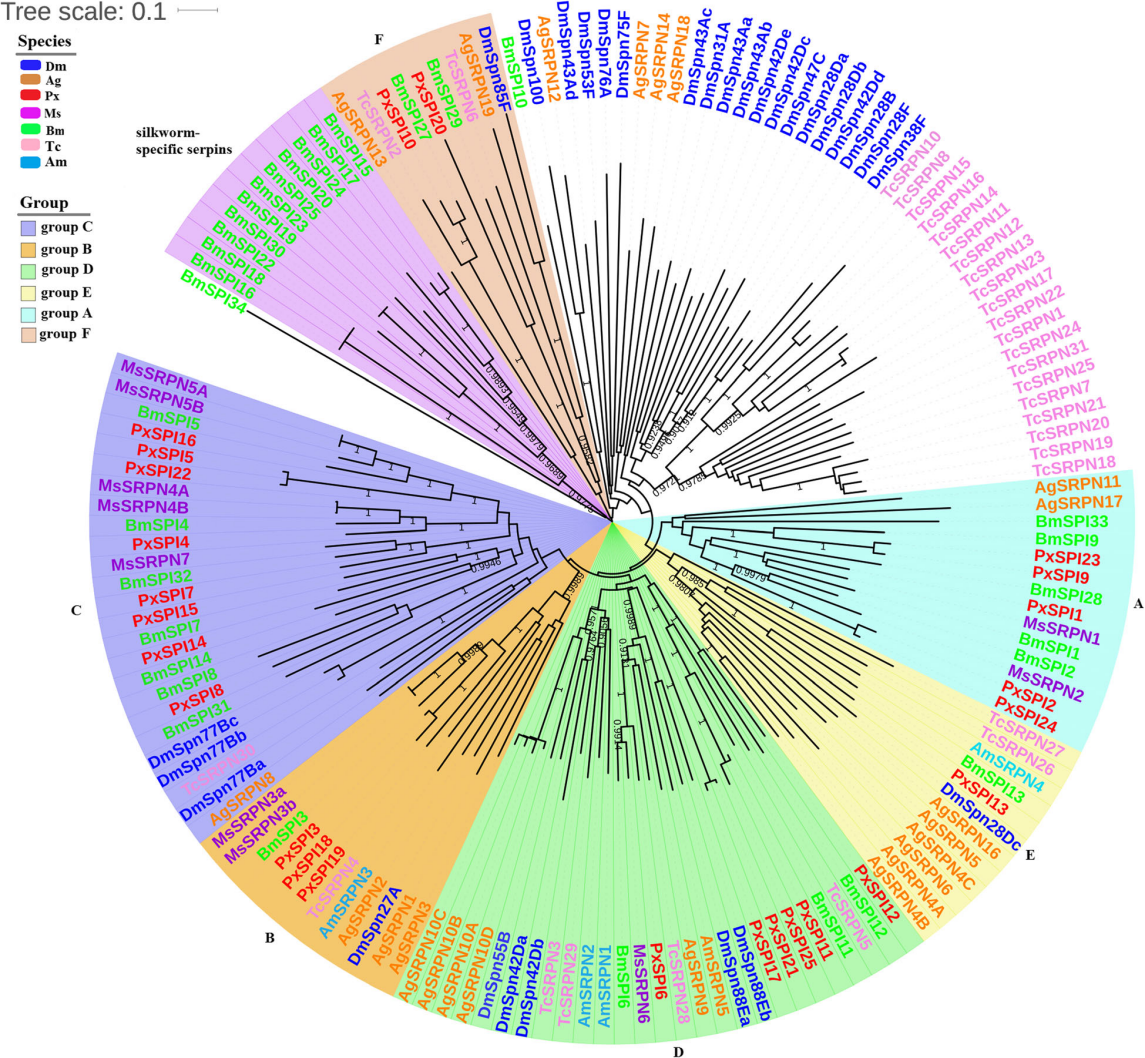
Phylogenetic relationship of serpins from *P. xylostella* and six other insect species. The phylogenetic tree was constructed using MEGA 6.06 with neighbor joining approach on the basis of Poisson model and pairwise deletion of gaps. The bootstrap scores higher than 0.9 are indicated on the nodes. *P. xylostella* serpins clustered to six distinct phylogenetic groups (A through F). The first two letters in each of the serpins represent the acronym of scientific name for a given species (Dm: *D. melanogaster*; Bm: *B. mori*; Ms: *M. sexta*; Ag: *A. gambiae*, Px: *P. xylostella*; Am: *A. mellifera*; Tc: *T. castaneum*)

Phylogenetic analysis of *P. xylostella* serpins with those from two other Lepidoptera species (*B. mori* and *M. sexta*), two Diptera species (*D. melanogaster* and *A. gambiae*), a Coleopteran species (*T. castaneum*) and a Hymenoptera species (*A. mellifera*) showed that *P. xylostella* serpins were divided into six distinct phylogenetic groups (groups A-F). Although distributed to different branches, *P. xylostella* serpins clustered with those of two Lepidoptera species (Fig. 3), indicating that the serpin family is highly conserved in Lepidoptera. However, *P. xylostella* serpins were not grouped with silkworm-specific serpins originally defined as group F that are arisen from recent gene duplications [21]. Except for silkworm-specific serpins, *P. xylostella* serpins were clustered with *M. sexta* serpins and the remaining *B. mori* serpins. For example, five *P. xylostella* serpins were clustered with two *M. sexta* and three *B. mori* serpins in group A. PxSPI1 was clustered with MsSRPN1J and BmSPI1, PxSPI2 and PxSPI24 were clustered with BmSPI2 and MsSRPN2, and so were PxSPI9 and PxSPI23 with BmSPI9. It has been shown that MsSRPN1J (MsSRPN1 splicing isoform J) is an inhibitor of prophenoloxidase (proPO) activating protease 3 (PAP3) in *M. sexta* [54]. Multiple sequence alignments of PxSPI1, MsSRPN1J and BmSPI1 showed that the three serpins had a high level of sequence identity (65.7%) and shared the identical amino acid residues at P1 position and hinge region (Additional file 2: Figure S2A), suggesting that PxSPI1 may function as an inhibitor of PAP in *P. xylostella* proPO activation system. In *M. sexta*, MsSRPN2 has been reported as an intracellular serpin, and its expression increases dramatically after larvae are injected with bacteria [38]. PxSPI2 lacked putative secretion signal peptide and was predicted to be an intracellular protein.

Group B consisted of *M. sexta*, *B. mori* and *A. mellifera* serpin3, TcSRPN4, DmSpn27A, *A. gambiae* serpin1-3. Previous studies indicated that MsSRPN3 and Dmspn27A play major roles in regulation of proPO and spätzle activation [39, 55], and recombinant AgSRPN1 and AgSRPN2 can function as inhibitors of PAP3 [56]. Sequence alignment showed that PxSPI3, 18 and 19, BmSPI3, MsSRPN3a, MsSRPN3b, DmSpn27A, along with AgSRPN1, 2 and 3 shared 50.5% sequence identity and the conserved hinge regions as well as Lys (K) at their P1 positions (Additional file 2: Figure S2B) except for PxSPI19 and AgSRPN3. Therefore, we assume that PxSPI3 and 18 might have similar regulatory functions in proPO and spätzle activation in *P. xylostella*. In Group C, eight *P. xylostella* serpins clustered with *M. sexta* and *B. mori* serpins that were originally defined as group C [21]. The alignment showed that residues at the P1 and P1’ positions (Arg and Ile) and hinge region in PxSPI4, BmSPI4, MsSRPN4A and MsSRPN4B were identical (Additional file 2: Figure S2C) and sequence identity among the four serpins is 77.5%. PxSPI5, 16 and 22, BmSPI5, MsSRPN5A and MsSRPN5B also shared a high sequence identity (67.6%) with the predicted P1-P1’ (Arg-Ile/Ser) residues (Additional file 2: Figure S2D). In *M. sexta*, MsSRPN4 and MsSRPN5 can regulate proPO activation by inhibiting the target proteases upstream of PAPs [40, 57]. In *B. mori*, BmSPI5 functions as an inhibitor to form covalent complexes with either BmSP21 or BmHP6 in both the proPO activation and AMP-producing pathways [58]. Therefore, we suggest that PxSPI4, 5, 16 and 22 may be involved in regulating proPO activation by inhibiting the target proteases upstream of PAPs. Additionally, PxSPI15 was clustered with BmSPI7, PxSPI14 was clustered with BmSPI14, and PxSPI8 was clustered with BmSPI8 and BmSPI31. However, the functions of these serpins are unclear. PxSPI7, MsSRPN7 and BmSPI32 shared 51.7% sequence identity and relatively conserved hinge region, and the residues at the P1 and P1’ positions in PxSPI7 were Arg and Ile, identical to those in MsSRPN7 (Additional file 2: Figure S2E).

Group D was composed of 27 serpins from *P. xylostella*, *B. mori*, *M. sexta*, *D. melanogaster*, *A. gambiae*, *T. castaneum* and *A. mellifera*. PxSPI6 was homologue to BmSPI6, MsSRPN6, AgSRPN9, DmSpn88Ea and DmSpn88Eb, and they shared 67.8% sequence identity and the strictly conserved hinge region with identical Arg and Ser residues at P1 and P1’ positions (Additional file 2: Figure S2F). Previous studies suggest that MsSRPN6 and MsSRPN7 with Arg (R) at the P1 position are likely involved in regulation of proPO activation in plasma by inhibiting PAPs [13, 42]. Therefore, we assume that PxSPI6 and PxSPI7 may also function as inhibitors of the melanization cascade. Group E contained PxSRPN13, DmSpn28Dc, BmSPI13, AmSRPN4, TcSRPN26, TcSRPN27 and *A. gambiae* serpin 4–6 and 16. *A. gambiae* serpin 4–6 and 16 have been reported as mosquito-specific expansions [20, 59], and biochemical analysis indicated that AgSRPN6 is an inhibitor of trypsin-like serine proteases [60]. In group F, PxSPI10 was grouped with BmSPI27, TcSRPN2 and AgSRPN13, PxSPI20 was clustered with BmSPI29, AgSRPN19, TcSRPN6 and DmSpn85F. In *D. melanogaster*, DmSPN85F functions as a non-inhibitory serpin, and it is highly conserved in all the 12 sequenced Drosophilid genomes [15, 34]. *A. gambiae* SRPN13 and SRPN19 also have been predicted as non-inhibitor serpins [20]. Furthermore, PxSPI10 and PxSPI20 had the least conserved residues in the hinge region (Fig. 2), supporting a prediction that they may not function as inhibitors.

### Canonical SPIs

A total of 33 SPIs genes were identified as canonical SPIs and were divided into seven different families (Trypsin Inhibitor like cysteine rich domain (TIL), Kunitz, Kazal, amfpi, Pacifastin, Antistasin and whey acidic protein (WAP); Additional file 1: Table S1). Seven of the eight SPI families found in *B. mori* [43] were identified in *P. xylostella*, except for Bowman-Birk family. Sequence alignments showed that the numbers and positions of Cys residues in the same family were highly conserved in *P. xylostella* canonical SPIs (Additional file 2: Figure S3). For instance, three pairs of disulfide bonds were identified in the Kunitz, Kazal, Pacifastin and Antistasin families, followed by four pairs in WAP, five pairs in TIL, and six pairs in amfpi (Additional file 2: Figure S3).

Domain architecture analysis suggests that 11 out of the 33 canonical inhibitors were single-domain proteins, while the remaining 22 SPIs encompassed multiple domains (Table 1 and Additional file 1: Table S1). Proteolytic cleavage sites of *P. xylostella* canonical SPIs were investigated using sequence alignment with known SPIs. The P1 residue in the cleavage site determines the specific inhibitory activity of SPIs, with Arg or Lys in the P1 site indicating the inhibitory activity to trypsin-like enzymes, Ala, Val or Gly showing the inhibitory activity to elastase-like enzymes, and Phe, Tyr or Leu suggesting the inhibitory activity to chymotrypsin-like enzymes [43]. Furthermore, it has also been reported that a Thr residue at the P1 position of the FPI-F scissile bond has inhibitory activity to subtilisin and some fungal serine proteases [61]. The alignments indicated that PxSPI34 (TIL family) had a Leu residue at the P1 position, suggesting that it may be involved in inhibiting chymotrypsin-like enzymes; PxSPI43 (WAP family) had a Val residue at the P1 site with predicted inhibitory activity against elastase-like enzymes; PxSPI40 (Kunitz family) and PxSPI57 (amfpi family) had an Arg/Lys (R/K) at the predicted P1 position, suggesting they may interact with trypsin-like enzymes (Additional file 2: Figure S3).

In addition to single-domain inhibitors, 22 canonical PxSPIs contained two or more inhibitor domains (Table 1 and Additional file 1: Table S1). Other than PxSPI28 (containing 17 tandemly arranged TIL domains), the other 21 PxSPIs contained 15 or fewer inhibitor domains (Table 1 and Additional file 1: Table S1). Previous studies show that SPIs containing multiple inhibitor domains have various residues at the P1 positions, suggesting that they are likely to have different inhibitory activities [43, 62]. We found that PxSPI32 consisted of two TIL domains, with Ala and Lys residues at the P1 site of the first and second domains, respectively. PxSPI39 comprised two Kunitz domains, with Arg and Phe residues at the P1 site of the first and second domains, respectively. PxSPI48 contained four Kazal domians, with Ala residue at the P1 site of the first domain, and Arg residues at the P1 sites of the second, third and four domains. PxSPI51 and PxSPI54 contained three Kazal domains, with Phe, Arg and Lys residues at the P1 site of the first, second and third domains, respectively. PxSPI55 contained two Kazal domains, with Lys and Arg residues at the P1 site of the first and second domains, respectively. PxSPI58 consisted of four Pacifastin domains, with Arg residue at the P1 site of the first and second domains, and Leu residue at the P1 site of the third and fourth domains. Thus, PxSPI55 may have inhibitory activity to trypsin-like enzymes, PxSPI32 and PxSPI48 may have inhibitory activity against elastase-like and trypsin-like enzymes, PxSPI39, PxSPI51, PxSPI54 and PxSPI58 may have inhibitory activity to both trypsin-like and chymotrypsin-like enzymes.

**Fig. 4:**
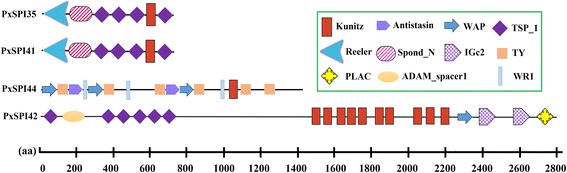
Domain organization of four *P. xylostella* SPIs in the Kunitz family. The sizes of domains are indicated by the scale

In addition, four SPIs, PxSPI35, PxSPI41, PxSPI42 and PxSPI44, comprised both inhibitor and non-inhibitor domains (Fig. 4). For instance, PxSPI35 and PxSPI41 were composed of one Kunitz domain, one reeling domain, one spond_N domain and four thrombospondin type 1 ﻿(TSP_1) ﻿ domains, similar to the structure of *B. mori* BmSPI55 [43]. PxSPI42 had various types of domains, including Kunitz, WAP, TSP_1, immunoglobulin C-2 type (IGc2), ADAM_spacer1 and PLAC (protease and lacunin) domains. Moreover, PxSPI44 contained three inhibitor domains (Kunitz, WAP and Antisasin) and two non-inhibitor domains (thyroglobulin type I repeat (TY) and worm-specific repeat type 1 (WR1)).

### Alpha-2-macroglobulins

In our study, three α_2_M genes were identified in the *P. xylostella* genome (Additional file 1: Table S1). To clarify the conserved and diverged residues of *P. xylostella* α_2_Ms, amino acid sequences of α_2_Ms were aligned and compared with other known α_2_M sequences using Clustal X and GeneDoc (Additional file 2: Figure S4). The results showed that PxSPI60 and PxSPI61 were composed of three functional domains required for the α_2_M mechanism, including a bait region, a thiol ester domain, and a receptor-binding domain, whereas PxSPI59 lacked the receptor-binding domain. The amino acid residues in the thiol ester domain in *P. xylostella* α_2_Ms were GCGEQNM, which are identical to those of α_2_Ms in other species. The region around the thiol ester domain of *P. xylostella* α_2_Ms showed similarity to the corresponding region in other α_2_Ms. Nevertheless, the similarity of the bait region between α_2_Ms of *P. xylostella* and other species was low as previously reported [63–66]. A completely conserved sequence of FPETW in the bait region of α2Ms in other species was replaced with FQEAW in PxSPI59 and PxSPI61, and with FPEAW in PxSPI60. The alignment also showed that the receptor binding regions of PxSPI60 and PxSPI61 shared high similarity with those of other α_2_Ms, and a consensus sequence of GGxxxTQDT was found in all the aligned sequences with GGMTNTQDT sequence in PxSPI60 and PxSPI61. Additionally, two amino acid blocks (Box1 and Box 2) (Additional file 2: Figure S4) in the N-terminus were conserved in all the aligned sequences, suggesting that these blocks may have an important functional or structural role. A neighbor-joining phylogenetic tree was constructed with the amino acid sequences of α_2_Ms, complement proteins and thioester-containing proteins (TEPs) using MEGA software (version 6.06) (Additional file 2: Figure S5), and the tree contained three main distinct branches, including α_2_Ms, TEPs and complement proteins. *P. xylostella* α_2_Ms were clustered with insect α_2_Ms, and they apparently are a monophyletic group.

### Expression profiling of *P. xylostella* SPIs

#### Stage-specific expression pattern

**Fig. 5:**
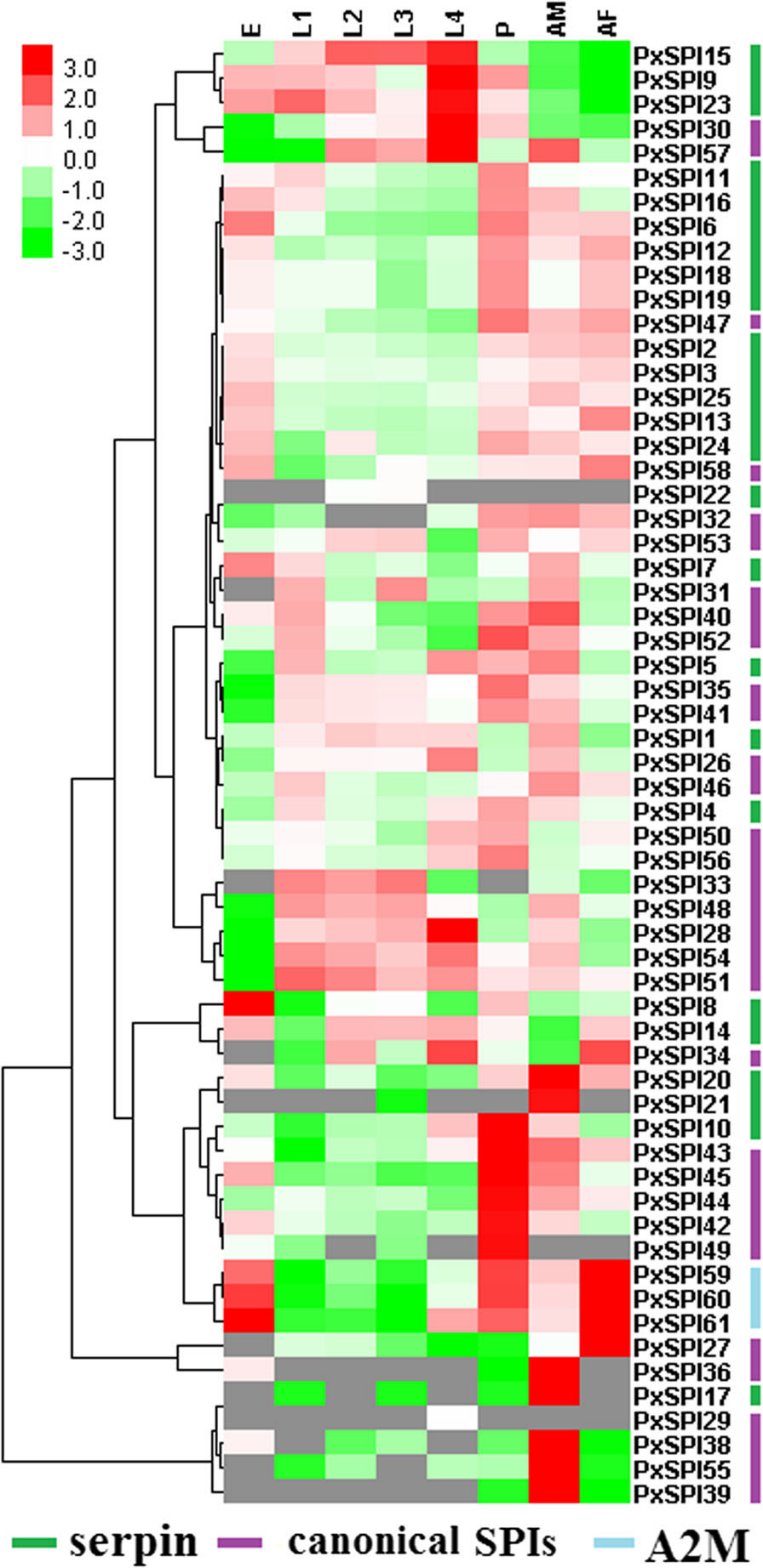
Expression profiling of PxSPIs at different developmental stages. The log2 RPKM values are colored, where red represents higher expression, green represents lower expression, and gray represents the RPKM values missed. E, eggs; L1-L4, 1st, 2nd, 3rd and 4th-instar larvae; P, pupae; AM, adult males; AF, adult females

Using RNA-seq data, expression patterns of PxSPIs at different developmental stages of the insecticide-susceptible strain (Fuzhou-S), including egg, larva, pupa and adult, were characterized (Fig. 5 and Additional file 1: Table S3). The results showed that PxSPIs exhibited gene-differential, stage- and sex-specific patterns. For example, PxSPI15 and PxSPI33 were expressed at high levels exclusively in the larval stage. Sixteen PxSPIs (PxSPI2-3, 6, 12–13, 16, 20 and 24–25 from the serpin family, PxSPI42-43 from the WAP family, PxSPI58 from the Pacifastin family, and PxSPI59-61 from the α2M family) were expressed at moderate or high levels in eggs, pupae and adults, but were undetectable or at very low levels in the larval stage. Seven PxSPIs (PxSPI17 and PxSPI21 from the serpin family, PxSPI27 from the TIL family, PxSPI36 and 38–39 from the Kunitz family, and PxSPI55 from the Kazal family) displayed sex-specific patterns and were exclusively expressed at high levels in adults, with PxSPI27 highly expressed in females and the other six genes highly expressed in males. PxSPI9 and PxSPI23 were expressed predominantly in egg, larval and pupal stages, rather than in the adult stage. Five SPIs (PxSPI28 and 33 from the TIL family, and PxSPI48, 51 and 54 from the Kazal family) showed moderate transcript levels in larvae and adult males, and no expression was detected in eggs, pupae and adult females. The stage-specific expression patterns of 12 genes (Additional file 2: Figure S6) was confirmed by qPCR and the results were consistent with the RNA-seq data.

### Tissue-specific expression pattern

RNA-seq analysis showed that 56 of the 61 PxSPI genes exhibited expression in at least one tissue (Additional file 1: Table S4 and Additional file 2: Figure S7). The midgut of 4th-instar larvae had fewer number (5) of genes expressed among the four tissues, while the numbers of genes expressed in the heads of 4th-instar larvae, adult males and females were 32, 28 and 34, respectively. PxSPIs had inverse tissue distribution tendency when compared with PxSPs and PxSPHs (the highest numbers of SPs and SPHs were distributed in the midgut) [67]. Such a tendency was also documented in *B. mori* [43]. Five PxSPIs, including 2 serpins (PxSPI1 and PxSPI14) and three Kazals (PxSPI51, 54 and 55), were expressed in the 4th-instar larval midguts and heads, while 15 PxSPIs were all highly expressed in the heads of adult males and females. Thirteen PxSPIs (PxSPI2-6, 10–12 and 16 from the serpin family, PxSPI26 and PxSPI28 from the TIL family, and PxSPI45 and PxSPI48 from the Kazal family) were expressed at a higher level in the heads of both larvae and adults than in the midgut of the 4th-instar larvae.

## Discussion

In the current study, we performed an overall analysis of SPI genes in *P. xylostella*, including analysis of their sequences, scaffold location, phylogeny, and expression profiles. A total of 61 SPI genes were identified in the *P. xylostella* genome, which were divided into nine different families based on the sequence similarity in amino acids. The number of SPI genes in *P. xylostella* is less than that in *B. mori* (80) [43]. This may result from fewer serpin (25) and TIL genes (9) identified in *P. xylostella*, compared to 34 annotated serpin and 15 TIL genes in the *B. mori* genome. In *B. mori*, eight silkworm-specific serpins and 9 genes of the TIL family have been found to come from tandem repeat evolution, and they may play important roles in protecting silk or protecting the cocoon from complicated environment conditions [21, 43].

Serpins are a superfamily of proteins with a number of functions, such as regulation of innate immunity, acting as tumor suppressors, hormone transports and molecular chaperones [15, 20, 21, 25–27]. A total of 25 *P. xylostella* serpins were identified, with each containing three β sheets and seven to nine α helices (Additional file 2: Figure S1), suggesting the overall folding of serpins remain largely unchanged in the long history of evolution [19, 68]. Mature *P. xylostella* serpin proteins were found to contain 379 ~ 503 aa with theoretical molecular masses of 41–55 kDa (Additional file 1: Table S1), consistent with previously documented sizes of serpins (350–500 aa) with molecular masses of 40–60 kDa [19, 69]. Moreover, we predict that the 20 *P. xylostella* serpins (PxSPI 1–7, 9, 11–18, 21–24) might be inhibitory serpins based on the conserved residues in the hinge region that can serve as a theoretical guideline to identify inhibitory serpins [20, 50]. Based on the results of sequence alignments and predicted proteolytic cleavage sites, we would suggest that 14 of the 25 *P. xylostella* serpins (PxSPI1-7, 11–12, 15–16, 18, 22 and 24) function as protease inhibitors. Phylogenetic analysis further confirmed that 14 putative *P. xylostella* inhibitory serpins were clustered with the known inhibitory serpins in other insect species (Fig. 3). Nine of 14 putative *P. xylostella* inhibitory serpins (PxSPI1, 3–7, 16, 18 and 22) were clustered with the orthologues in *M. sexta* (MsSRPN1 and 3–7) with a high sequence identity. These *M. sexta* serpins have been demonstrated to participate in regulating the prophenoloxidase activation cascade [13, 39, 40, 42, 54, 57]. Therefore, we propose that nine *P. xylostella* serpins may play vital roles in regulation of innate immunity in *P. xylostella*. PxSPI10 and PxSPI20 lack the characteristic features of inhibitory serpins, however, they were found to be highly expressed in pupae and adult males, respectively. Some serpins do not function as inhibitors, and others do inhibit proteases even though the conserved features are missing [70, 71]. It is worth testing the biological roles of PxSPI10 and PxSPI20 by genetic and biochemical studies.

Apart from the serpin family, the current work identified 33 SPIs belonging to canonical SPIs that contained rich cysteine residues to form disulfide bonds to keep their rigid structure. The rigid structure are necessary for keeping right conformation to interact with the active site of serine proteases [72]. And 11 of the 33 canonical inhibitors were single-domain proteins with low molecular weights, exemplified by PxSPI57 that contained an amfpi domain with a low molecular weight (8.97 kDa). A BLAST search of GenBank™ revealed that PxSPI57 was similar in amino acid sequence to the small cationic peptide (MsCP8) in the hemolymph of *M. sexta* larvae, BmSPI69 (*B. mori*), and a fungal protease inhibitor-1 of *Antheraes mylitta* (AmFPI-1). MsCP8 has a Lys residue at the P1 position, however, it shows no inhibitory activity against serine proteases [73]. AmFPI-1 shows inhibitory activity against some fungal serine proteases and trypsin [74], while BmSPI69 has a predicted P1 residue of Lys and may have inhibitory activity to trypsin-like enzymes [43]. Therefore, the exact function of PxSPI57 needs to be further studied. The remaining canonical PxSPIs contained two or more inhibitor domains, forming multi-domain compound inhibitors. The results were fully in consistent with those of previous work, in which the single inhibitor domain can be repeated 2–15 times to form multi-domain compound inhibitors in some SPI families [7, 8]. For instance, PxSPI26, PxSPI46 and PxSPI58 have 13 TIL, 6 Kazal, and 4 Pacifastin domains, respectively. In addition, some compound inhibitors contained inhibitor domains (from more than one family) and non-inhibitor domains, which are defined as mixed-type inhibitors, exemplified by PxSPI35, PxSPI41 and PxSPI42. PxSPI35 and PxSPI41 were homologous to BmSPI55 and F-spondin. F-spondin is an extracellular matrix protein that inhibits the outgrowth of embryonic motor axons in a contact-repulsion fashion [75]. BmSPI55 might play an important role in the innate immune system of *B. mori* [43]. BLAST search revealed that PxSPI42 was similar to BmSPI58 and papilin, which has a broad expression spectrum in different pericellular matrices in *Drosophila* embryos and is indispensable for embryonic development [76].

Gene expression profiles showed that PxSPI15 and PxSPI33 were expressed at high levels exclusively in the larval stage. Their expression patterns are extremely parallel to those of 50 serine protease genes previously found in *P. xylostella* [67]. Additionally, expression patters of seven PxSPIs (PxSPI17, 21, 27, 36, 38–39 and 55) are consistent with those of 16 serine protease genes highly and exclusively expressed in adult males and one highly expressed in adult females of *P. xylostella* [67], indicating that these PxSPIs may participate in regulation of the activity of serine proteases in adults. Their functions still need to be validated by molecular studies.

## Conclusions

It is by far the most comprehensive research on whole-genome identification and expression profiling of SPIs in *P. xylostella*. We explored the sequences and possible physiological functions of *P. xylostella* serpins and canonical SPIs, revealing that nine *P. xylostella* serpins may have inhibitory activity and be involved in regulation of the immune system of an important agricultural pest, and some canonical SPIs may have inhibitory activity to trypsin/chymotrypsin/elastase-like enzymes. However, additional biochemical and biological studies are required to determine and validate their functions. Analyses of sequences, substrate specificity and domain architecture of alpha-2-macroglobulins will also be useful for further molecular genetic studies in *P. xylostella*. This article provides an overview of the *P. xylostell*a SPIs, and will facilitate future functional investigation.

## Methods

### Identification of SPI genes in *P. xylostella*

SPI protein sequences of *D. melanogaster*, *A. mellifera*, *T. castaneum*, *A. gambiae*, *B. mori* and *M. sexta* were downloaded from their genome databases [77–81] and/or the NCBI (National Center for Biotechnology Information) (http://www.ncbi.nlm.nih.gov/), and then used as queries against the DBM database [49, 82] using local TBLASTN program (E-value < 10^−7^). We preformed online FGENESH+ programs (http://www.softberry.com/) to find complete open reading frames (ORFs) of SPI genes with the method used by Yu et al. and You et al. [83, 84]. These putative protein sequences were manually confirmed using NCBI online BLASTP.

### Characterization of PxSPIs

Domain architectures of PxSPI genes were predicted by NCBI CDD database (http://www.ncbi.nlm.nih.gov/Structure/cdd/docs/cdd_search.html) and SMART (http://smart.embl-heidelberg.de/). Theoretical molecular weight and isoelectric point of proteins were calculated based on the predicted sequences using Compute pI/Mw tool (http://web.expasy.org/compute_pi/). The signal peptides and their cleavage sites of SPIs were analyzed by online SignalP 4.0 (http://www.cbs.dtu.dk/services/SignalP/). Multiple sequence alignments of SPI protein sequences were performed by ClustalX 2.0 program with default parameters [85], and alignments were manually modified using GeneDoc (http:www.nrbsc.org/gfx/genedoc/index.html). The identical amino acids and blocks of conserved amino acid residues are shaded in black and gray, respectively. The reactive sites of SPIs were marked with asterisks.

### Phylogenetic analyses

The phylogenetic relationships of SPI genes were compared among seven insect species: *A. gambiae*, *D. melanogaster*, *M. sexta*, *B. mori*, *T. castaneum*, *A. mellifera* and *P. xylostella*. Putative amino acid sequences of SPIs were aligned using ClustalX 2.0 [86]. A phylogenetic tree was constructed with MEGA 6.06 using the neighbor-joining method by Poisson model with bootstrap test (1000 replicates) [87].

### Expression profiling and qPCR analysis

Based on the RNA-seq data from different developmental stages and tissues previously completed in our laboratory, expression profiling of the 61 SPI genes were analyzed and clustered by Cluster 3.0 and visualized by Java TreeView [88]. The developmental stages (E: eggs; L1-L4: 1st, 2nd, 3rd and 4th instar larvae; P: pupae; AM: adult males; AF: adult females) and tissues (L4M & L4H: midguts and heads of 4th-instar larvae; and AMH & AFH: heads of adult males and adult females) were used in this study. The RPKM values were log_2_ transformed, and the clustered genes were illustrated in terms of their expression patterns using the similarity metric of Euclidean distance and clustering method of complete linkage [67, 83].

A susceptible strain of *P. xylostella* (originally collected from a vegetable field in Fuzhou) was reared on radish seedlings at 25 ± 2 °C, 70 ~ 80% RH, 16 : 8 h = light : dark cycle and used for genome sequencing [49]. Stage-specific diamondback moth samples (newly laid eggs, 1st-4th-instar larvae, pupae and unmated or mated adult males and females) were collected and stored at −80 °C until RNA extraction. The total RNA of each sample was extracted using TRIzol reagent (Invitrogen, USA) and digested with 1 μL gDNA Eraser (Takara Biotechnology (Japan) Co., Ltd.) for 2 min at 42 °C to remove contaminating genomic DNA. The template (cDNA) for qPCR was synthesized with total RNA (1 μg) using PrimeScriptTM RT reagent Kit (TaKaRa, Japan) according to the manufacturer’s instructions. qPCR was carried out using a CFX96 Touch™ Real-Time PCR Detection System (Bio-Rad, USA). The 20 μL mixture included 10 μL 2× real-time PCR Mix (containing SYBR Green I), 2 μL cDNA template from the relative sample (100 ng/μL), 0.4 μL of each primer (10 mmol/L), and 7.2 μL nuclease-free water in each well of a 96-well plate. PCR was conducted with an initial denaturation at 95 °C for 3 min, followed by 40 cycles at 95 °C for 10 s and 60 °C for 30 s, and a final melting curve starting at 63 °C for 5 s up to 95 °C with 0.5 °C increments. Twelve SPI genes were selected for further validation of expression by qPCR and the primers were designed for qPCR performance (Additional file 1: Table S5). The *P. xylostella* ribosomal protein gene L32 was used as the housekeeping reference (forward primer: 5′-AAT CAG GCC AAT TTA CCG C-3′; reverse primer: 5′-CTG GGT TTA CGC CAG TTA CG-3′). Relative gene expression data were normalized against Ct values for the housekeeping gene.

The qPCR data were statistically analyzed using the R statistical program version 3.0.2, with the supplemented package ‘agricolae’ [89]. If the data satisfied normality assumption, one-way ANOVA was performed, otherwise the Kruskal-Wallis nonparametric test was used.

## Additional files

Additional file 1: Table S1. Description of serine protease inhibitor (SPI) genes in *P. xylostella*.** Table S2.** The predicted amino acid sequences of 61 SPIs. **Table S3.** RPKM values of the PxSPI genes at different developmental stages obtained from the RNA-seq data. **Table S4.** RPKM values of the PxSPI genes at different tissues obtained from the RNA-seq data. **Table S5.** Primers used for qPCR study on gene expression. (DOCX 52 kb)
Additional file 2: Figure S1. Multiple sequence alignment of 25 *P. xylostella* serpins with known serpins from *M. sexta* (MsSRPN3-7), *D. melanogaster* (DmSpn27A) and *A. gambiae* (AgSRPN9) using Clustal X. **Figure S2.** Alignment of *P. xylostella* serpins with some known serpins from other insect species by Clustal *X*2. A, Alignment of PxSPI1 with MsSRPN1J and BmSPI1; B, Alignment of PxSPI3, 18 and 19 with MsSRPN3a, 3b, BmSPI3 and AgSRPN1, 2, 3; C, Alignment of PxSPI4 with MsSRPN4a, MsSRPN4b and BmSPI4; D, Alignment of PxSPIs 5, 16 and 22 with MsSRPN5A, 5B and BmSPI5. E, Alignment of PxSPI7 with MsSRPN7 and BmSPI7. F, Alignment of PxSPI6 with MsSRPN6, BmSPI6, AgSRPN9, DmSpn88Ea and DmSpn88Eb. **Figure S3.** Alignment of serine protease inhibitor domains using Clustal *X*2 with default parameters and shading was done using GeneDoc. (A) TIL family, (B) Kunitz family, (C) WAP family, (D) Kazal family, (E) amfpi family, (F) Antistasin family, (G) Pacifastin family.** Figure S4.** Multiple sequence alignment of *P. xylostella* α2Ms with other α2Ms using Clustal *X*2. **Figure S5.** Phylogenetic tree of α2Ms, complement proteins and thioester-containing proteins constructed using the neighbor joining method. **Figure S6.** qPCR-based expression profiling of PxSPI genes across different developmental stages. **Figure S7.** Expression profiling of the *P. xylostella* SPI genes in different tissues. (DOCX 8635 kb)

## Abbreviations

α2M: Alpha-2-macroglobulin
aa: Amino acid
ADAM_spacer1: Spacer-1 domain from the ADAM-TS family of metalloproteinase
AgSRPN9: *A. gambiae* serpin9
Am: *Apis mellifera*
AmFPI-1: A fungal protease inhibitor-1 of *Antheraes mylitta*
Bm: *Bombyx mori*
Dm: *Drosophila melanogaster*
DmSpn27A: *D. melanogaster* serpin27A
IGc2: Immunoglobulin C-2 type
Ms: *Manduca sexta*
MsCP8: A small cationic peptide in the hemolymph of *M. sexta*
MsSRPN 3–7: *M. sexta* serpin 3–7
NCBI: National Center for Biotechnology Information
PAPs: Prophenoloxidase activating proteases
PLAC: Protease and lacunin
PO: Phenoloxidase
proPO: prophenoloxidase
Px: *Plutella xylostella*
RCL: Reactive center loop
SPIs: Serine protease inhibitors
Tc: *Tribolium castaneum*
TEPs: Thioester-containing proteins
TIL: Trypsin inhibitor-like cysteine-rich domain
TSP_1: Thrombospondin type 1 repeat
TY: Thyroglobulin type I repeat
WAP: Whey acidic protein
WR1: Worm-specific repeat type 1
