# Supplementary material for: Characterization and expression profiling of serine protease inhibitors in the diamondback moth, Plutella xylostella (Lepidoptera: Plutellidae)
Source: BMC Genomics. 2017 Feb 14;18:162. doi: 10.1186/s12864-017-3583-z (PMC5309989; doi:10.1186/s12864-017-3583-z)
Supplement: Additional file 1: Table S1. — Description of serine protease inhibitor (SPI) genes in P. xylostella. Table S2. The predicted amino acid sequences of 61 SPIs. Table S3. RPKM values of the PxSPI genes at different developmental stages obtained from the RNA-seq data. Table S4. RPKM values of the PxSPI genes at different tissues obtained from the RNA-seq data. Table S5. Primers used for qPCR study on gene expression. (DOCX 52 kb) [file 12864_2017_3583_MOESM1_ESM.docx]

**Supplemental Tables**

**Additional file 1: Table S1**

Description of serine protease inhibitor (SPI) genes in *P. xylostella*.

**Additional file 1: Table S2**

The predicted amino acid sequences of 61 SPIs.

**Additional file 1: Table S3**

RPKM values of the PxSPI genes at different developmental stages obtained from the RNA-seq data.

**Additional file 1: Table S4**

**Table S4** RPKM values of the PxSPI genes at different tissues obtained from the RNA-seq data

**Additional file 1: Table S5**

Primers used for qPCR study on gene expression.

**Table S1** Description of serine protease inhibitor (SPI) genes in *P. xylostella*

| Gene ID | Name^a^ | SPI Domain | No. of domain | Length (residues)^b^ | Locus^c^ | Gene size (bp) | Signal peptide | pI | MW (kDa) | Exon | ortholog in *Manduca sexta* | ortholog in *Bombyx mori* |
| --- | --- | --- | --- | --- | --- | --- | --- | --- | --- | --- | --- | --- |
| Px001494 | PxSPI1 | serpin | 1 | 379 | scaf_12 | 15682 | no | 5.10 | 41.93 | 8 | MsSRPN1J | BmSPI1 |
| Px004227 | PxSPI2 | serpin | 1 | 503 | scaf_17 | 26806 | no | 9.01 | 55.43 | 9 | MsSRPN2 | BmSPI2 |
| Px017059 | PxSPI3 | serpin | 1 | 449 | scaf_879 | 7923 | no | 6.28 | 50.14 | 8 | MsSRPN3a -3b | BmSPI3 |
| Px007271 | PxSPI4 | serpin | 1 | 18+394 | scaf_258 | 1239 | SFG-QQ | 7.05 | 44.69 | 1 | MsSRPN4A-4B | BmSPI4 |
| Px007273 | PxSPI5 | serpin | 1 | 18+378 | scaf_258 | 1191 | CVC-AV | 5.44 | 43.18 | 1 | MsSRPN5A-5B | BmSPI5 |
| Px002424 | PxSPI6 | serpin | 1 | 17+396 | scaf_137 | 9262 | AAG-QC | 5.19 | 44.48 | 7 | MsSRPN6 | BmSPI6 |
| Px007274 | PxSPI7 | serpin | 1 | 16+382 | scaf_258 | 1197 | VLT-AP | 5.08 | 42.82 | 1 | MsSRPN7 | BmSPI32 |
| Px013290 | PxSPI8 | serpin | 1 | 21+366 | scaf_54 | 1164 | VVG-ET | 5.51 | 41.02 | 1 |  | BmSPI8 |
| Px010990 | PxSPI9 | serpin | 1 | 395 | scaf_408 | 8211 | no | 5.59 | 43.68 | 8 |  | BmSPI9 |
| Px002882 | PxSPI10* | serpin | 1 | >398 | scaf_144 | 1635 | no | 8.64 | 45.35 | >3 |  | BmSPI27 |
| Px015089 | PxSPI11 | serpin | 1 | 19+385 | scaf_69 | 4040 | LNG-QY | 5.90 | 43.62 | 6 |  | BmSPI11 |
| Px015087 | PxSPI12 | serpin | 1 | 364 | scaf_69 | 3405 | no | 8.70 | 33.87 | 6 |  | BmSPI12 |
| Px011577 | PxSPI13 | serpin | 1 | 432 | scaf_440 | 4694 | no | 5.53 | 48.46 | 8 |  | BmSPI13 |
| Px003822 | PxSPI14 | serpin | 1 | 21+389 | scaf_160 | 1233 | VVG-KQ | 5.97 | 43.74 | 1 |  | BmSPI14 |
| Px003823 | PxSPI15 | serpin | 1 | 403 | scaf_160 | 1538 | no | 4.99 | 44.41 | 2 |  | BmSPI7 |
| Px007272 | PxSPI16 | serpin | 1 | 17+380 | scaf_258 | 1194 | GNC-DI | 5.77 | 43.00 | 1 | MsSRPN5A-5B | BmSPI5 |
| Px014010 | PxSPI17 | serpin | 1 | 25+401 | scaf_6 | 8621 | VVG-SD | 5.84 | 44.11 | 9 |  |  |
| Px015090 | PxSPI18 | serpin | 1 | 441 | scaf_69 | 6404 | no | 5.36 | 49.26 | 7 | MsSRPN3a-3b | BmSPI3 |
|  | PxSPI19 | serpin | 1 | 382 | scaf_69 | 2531 | no | 9.09 | 43.04 | 7 | MsSRPN3a-3b | BmSPI3 |
| Px004226 | PxSPI20* | serpin | 1 | >309 | scaf_17 | 4928 | no | ? | >34.43 | >6 |  | BmSPI29 |
| Px002346 | PxSPI21 | serpin | 1 | 298 | scaf_1350 | 4779 | no | 6.46 | 33.25 | 7 |  |  |
| Px016549 | PxSPI22 | serpin | 1 | 316 | scaf_80 | 952 | no | 5.29 | 36.08 | 1 | MsSRPN5A-5B | BmSPI5 |
| Px003869 | PxSPI23 | serpin | 1 | 16+384 | scaf_161 | 10004 | CWA-YP | 6.31 | 42.34 | 9 |  | BmSPI9 |
| Px007765 | PxSPI24 | serpin | 1 | 28+381 | scaf_275 | 8125 | TQS-EE | 5.30 | 42.01 | 13 | MsSRPN2 | BmSPI2 |
| Px017058 | PxSPI25* | serpin | 1 | >164 | scaf_879 | 2870 | LNG-QY | ? | >21.99 | >3 |  |  |
| Px002767 | PxSPI26 | TIL | 13 | 18+1332 | scaf_141 | 74091 | SLA-EE | 5.43 | 146.95 | 20 |  |  |
| Px006736 | PxSPI27 | TIL | 6 | 1061 | scaf_237 | 12692 | no | 4.61 | 114.12 | 17 |  |  |
| Px006737 | PxSPI28 | TIL | 17 | 18+1934 | scaf_237 | 20929 | ANA-TA | 4.78 | 211.49 | 30 |  |  |
| Px006739 | PxSPI29 | TIL | 3 | 199 | scaf_237 | 1364 | no | 4.32 | 22.23 | 3 |  |  |
| Px006740 | PxSPI30 | TIL | 4 | 887 | scaf_237 | 11727 | no | 3.91 | 90.38 | 7 |  |  |
| Px007753 | PxSPI31 | TIL | 3 | 21+238 | scaf_275 | 8237 | VSS-LY | 4.35 | 25.49 | 5 |  |  |
| Px007757 | PxSPI32 | TIL | 3 | 23+209 | scaf_275 | 3594 | VFG-QV | 8.19 | 23.20 | 6 |  |  |
| Px010825 | PxSPI33 | TIL | 3 | 313 | scaf_4 | 4354 | no | 4.97 | 33.61 | 6 |  |  |
| Px011976 | PxSPI34 | TIL | 1 | 24+138 | scaf_464 | 1377 | VFS-TS | 8.50 | 15.44 | 3 |  |  |
| Px003939 | PxSPI35 | Kunitz | 1 | 16+743 | scaf_162 | 13213 | VDA-QD | 5.79 | 81.80 | 14 |  | BmSPI55 |
| Px011924 | PxSPI36 | Kunitz | 2 | 179 | scaf_46 | 2879 | no | 7.44 | 20.08 | 4 |  |  |
| Px011925 | PxSPI37 | Kunitz | 2 | 140 | scaf_46 | 4468 | no | 7.44 | 15.75 | 4 |  |  |
| Px011926 | PxSPI38 | Kunitz | 3 | 19+254 | scaf_46 | 2531 | TSM-AY | 8.07 | 28.27 | 5 |  |  |
| Px011927 | PxSPI39 | Kunitz | 2 | 17+154 | scaf_46 | 2963 | VYT-KI | 8.00 | 17.70 | 4 |  |  |
| Px013236 | PxSPI40 | Kunitz | 1 | 133 | scaf_534 | 2750 | no | 4.79 | 13.95 | 4 |  |  |
| Px009905 | PxSPI41 | Kunitz | 1 | 16+750 | scaf_356 | 14331 | VGA-QD | 6.49 | 82535.97 | 15 |  | BmSPI55 |
| Px012095 | PxSPI42 | Kunitz/  WAP | 10/1 | 2870 | scaf_471 | 47650 | no | 4.62 | 309871.67 | 54 |  | BmSPI58 |
| Px006046 | PxSPI43 | WAP | 1 | 374 | scaf_213 | 6771 | no | 9.84 | 42.51 | 7 |  |  |
| Px006986 | PxSPI44 | WAP/  Antistasin/Kunizt | 3/2/1 | 1441 | scaf_247 | 11948 | no | 5.06 | 156.43 | 22 |  |  |
| Px002484 | PxSPI45 | Kazal | 4 | 540 | scaf_139 | 6298 | no |  |  | 11 |  |  |
| Px003407 | PxSPI46 | Kazal | 6 | 21+646 | scaf_152 | 11118 | VGG-CY | 6.06 | 72.32 | 10 |  |  |
| Px006079 | PxSPI47 | Kazal | 1 | 174 | scaf_214 | 1484 | no | 4.83 | 18.64 | 4 |  |  |
| Px007758 | PxSPI48 | Kazal | 4 | 17+242 | scaf_275 | 1072 | AQS-AP | 5.86 | 25.25 | 2 |  |  |
| Px009964 | PxSPI49 | Kazal | 1 | 112 | scaf_36 | 2410 | no | 8.60 | 12.02 | 3 |  |  |
| Px010786 | PxSPI50 | Kazal | 1 | 90 | scaf_4 | 798 | no | 4.14 | 9.79597 | 2 |  |  |
| Px013782 | PxSPI51 | Kazal | 3 | 27+168 | scaf_584 | 783 | ACG-VC | 6.15 | 18.26 | 2 |  |  |
| Px013990 | PxSPI52 | Kazal | 5 | 1082 | scaf_6 | 45754 | no | 5.71 | 115.94 | 16 |  |  |
| Px013993 | PxSPI53 | Kazal | 1 | 135 | scaf_6 | 7673 | no | 6.96 | 14.11 | 3 |  |  |
| Px014318 | PxSPI54 | Kazal | 3 | 22+168 | scaf_62 | 2137 | ACG-VC | 6.31 | 18.31 | 2 |  |  |
| Px014322 | PxSPI55 | Kazal | 2 | 136 | scaf_62 | 411 | no | 8.21 | 15.19 | 1 |  |  |
| Px014515 | PxSPI56 | Kazal | 1 | 487 | scaf_635 | 23510 | no | 7.49 | 52036.05 | 7 |  |  |
| Px003613 | PxSPI57 | amfpi | 1 | 18+86 | scaf_158 | 1997 | VAG-DL | 8.63 | 8.97 | 2 | MsCP8 | BmSPI69 |
| Px013712 | PxSPI58 | Pacifastin | 4 | 19+451 | scaf_58 | 13847 | ANA-GA | 5.20 | 49.49 | 12 |  |  |
| Px015934 | PxSPI59 | A2M | 1 | 1063 | scaf_751 | 9874 | no | 8.95 | 117.34 | >19 |  |  |
| Px013431 | PxSPI60 | A2M | 1 | 1738 | scaf_55 | 21129 | no | 6.49 | 190.47 | 31 |  |  |
| Px013945 | PxSPI61 | A2M | 1 | 1727 | scaf_598 | 27514 | no | 6.32 | 188.98 | 31 |  |  |

a * represents partial gene (missing some exons or 5’ terminus and 3’ terminus).

b signal peptide+mature protein.

c scaffold number.

A2M: α2-macroglobulin

**Table S2** The predicted amino acid sequences of 61 SPIs

| Gene ID | Name | Amino acid sequences |
| --- | --- | --- |
| Px001494 | PxSPI1 | MADEIEDFKQQLNNGNDLFTANMFSEVLKQQNGKNVVLSAFSVLTPVAQLSLASSGETHDLLLKTLGLPSDEAIKSVFPLVNAKLRDIKGVTLKQASRIYLPLTAELADDFAAASRDVFGSEAKNLDFTKNVESAKEINDWVEEQTNNRIQDLVDPDSIKPNTASLLVNAIYFKGTWKSKFDNASTTERDFHVSKDKTVKVPTMYQKASFKYGESAELDAKLLEMPYEGEKASFLIILPNDIEGLPALTEKLKEPQHLNKALSEMYKAEIQVYIPKFKIETETDLKEVMSNMGLSSLFEPGKATIDKLLKSKAPLYIGDAKQKAFIEVNEEGSEAAAANVFSMLRSAKMAHPPFVADRPFVFSIQQASTSLFSGAFLDA |
| Px004227 | PxSPI2 | MTGGEGGKLKTPPERLRACGEGGKLKTPPARLVRSSRLFRETARRSRQSGAIIAWRISRLATGATAIRSPDTANEDTPKHSTLYVPNPHLLNYALRRNIQKYLQATESPLLTFELTNFRAKPPSPSSKTGQCPNYKFTLDRTHTAYVPGKNLVSSPVMVELLLGLLALGSKDPSQLIDVMHLGSNKDQLKVAFKKVSGAMQNIKGVTFYAANKLYVKECDSVVMPAFKKEAVHAFWAEVEKVDFDMGPVAADIISTWVEKITKGLIKDIIDPSTIESNTQAVMVNGLYFKGKWEKEFDPLDTETQTFHVSSSTAKNVQMMSQEETFKYGNCSALSAQLLELNYSKGKASMLIVLPKEVEGLKNVLRLLSDGYDLVSERGKMKPTRVQVRIPKFAIESDLDLSVLLPKMGVSSIFSSSAGITQILGNNESLEVSKGVQKAFLEVDEEGSKAASSSGMSLRSRRSVPEPPEQFVADRPALYIILLDDLPVLSAIYHPEPDTRDEL |
| Px017059 | PxSPI3 | MTVLFSSTPDIIPKPPLAHSANTGTPKPPTIDESVLQNVFGSPQSFYSQAVPVVPENGTLVDPDYWDSEDIYRPSSLTYDLFDLSITKRVAKSSPDNFLMSPLGLKLALAILTEAATGTTQAELVSVLNFDKDRNTVRHKFASIVSSLKTKSPQYILDLGSRIYVDNTAKTRQRFAAIAEQFYNTELQNIDLSKPGPAAKDINAWVSEKTQGRISQLVTEADLASAVVLILNTLYFRGTWRHQFAPNETAAAPFYVTPSVQKQAHFMKVQNSFYYAESMKFDAKILRMPYLGRKYSMNIIVPNSLSGLPRVVENLSELRQEMTFLTEKVVDVTIPRFQFDYTTHFENILKELGVREAFEDTASFPGISRGQTLRQRLKVSNVLQKAGIEVNELGSTAYSATVNKFGTDNDGMQEVIANKPFLFFIEDEATRQLLFSGRFADPTATIKMA |
| Px007271 | PxSPI4 | MWRLTSLVTLLCVCSSFGQQPVETTTPPYSLELHEGLSQSIGNFSVEILYHTAVSSLMANKNLVISPLTMWIALAVTNEGADGRTSKQIKDAIRSPIKKDEFGKIARWLKVNGSTVELQNINTIFVDVKNLMERDFRDVALRYYETQVNALDFQDKVGTANTINKRVSDITRGRIPKLVDSADFEQAQMLLISALYFKGQWTSPFNATQTAPRPFFDSNGKTIGTVNMMYNRYTYPFANIRELEARVIELPYGKENRLSMLIMLPNPNVSLENMFLKFATVPLDKVFQELRISQSEYSDDEVDCFIPRFKIESDLVLNSALNNMGIYDMFNPAKARLPKVSRVPVYVSKVIHKAEIEVNEEGTTASAVTAIEFANRIGIIRFEANRPFLYMIIEKVTNSIVFGGVYRQPSLY |
| Px007273 | PxSPI5 | MLFKIFIIFSLCVSLCVCAVEYSDRGRNFSLELIYFTGQENQNHFVVSPFSVWTLLTGLAYGTIGDIYRELRFALILPLKMSRTAEQYPEFMKAVFDIPKPEISVSTNSYIFYDNVVPVERDFMGTLERDFKTVVNQISPDNWTATDDIVNDIKKSHTHAVNVINLSNLQGAATMRAINFISFEGLRGVPFDKRDTAVNNFYNDQGDLIGEVNMMHQKGPFQFTDIKELEAVLIDLPFANNDKYSMLVILPYDKRNTTAVYRKFLTYSIPDIYKSLKDDLEAYGEEVVDLKIPRFRITDEIKMEKPLNSMGVYSLFEKSDFHRAVRLPVQISGFTQSVNIEITESGSVLGATKPKVQPIRSLISNIVVDRPFIFFIMEKSTLSILIGGIYSKPEKY |
| Px002424 | PxSPI6 | MLIGVLSALIMASSAAGQCFSSNDTSGKKALPEARTSLYRGQLEFTLNVFNAINKALPEDNIFFSPFSVYQSLLLAYFSAGGSTVESLREALVIDEKLDKASLMSAYKSDKWSRANNNNSDSYEFSSANKLFVEESLDVAPCTKEFFNEEMEPLDFQNAPTEAREHINQWVARVTKDNIKNLIPEDGIGQSTKLVLANAAYFKGVWASKFPAERTKKDVFYVSETRQTLVPFMKQKGSFHYTVNEDLGAQILELPYKGNDISMYIFLPPYSMKEGVTNIIANLTPERLAAVVEESYMSREVLVEIPKFTIERELPLRGVLESIGAGDLFDGKANFSSLTSQRDVAFDDAIHKAKITLDEEGTTAAAATAIFGFRSSRPVEPARFVANFPFVYILYERPTNSVLFMGVYRDPKK |
| Px007274 | PxSPI7 | MLKAVVIITLVNSVLTAPSGVNFSPINDFSFNLLQNVFAYQENFGPQNLAVSPVSVWNIFSLLSGGASGATFAELSQLLRLPKDVRVTQDLHLASNSVLKVKNKDVILNKQAAMFAERELQIHPEFCESASKYNTEIYTVDASNTTKLANDINYFICMATEGAIKDAVKPDLLENLRLLLVDAMYFKANWTTPFNSRQTKRETFYDQQGRSIGDVNMMYQKAPNKIFYAAELEADVLELTYGEDKEFAMVIFLPDYGVPLKRVLNNLSTKPTDAWTMLLQNEEQMSSVECQVPRFKISSQLDLIQPLKYMGLYSIFDEMKAELPGISDSPLYVSKTIQKTELEVTEEGTVAAVATVVGLENRILGPSINVNKPFVYAIVNRRHNIVLFTGVYTNPSVV |
| Px013290 | PxSPI8 | MRLLVLASCLITVAGVTLVVGETCRRGDVTTKFRRSLYDFACKLYKQVASQTDDHFAVSQYSIWLALAGMAEGASGDVRAEILATLKIPDPICLRLEYYELATELESSAEDVRLERTRSLLVDEDMKVNETWMRDVREVGLLDTVFTPQDKLRELLQVNDSSFHSIQAVLSDSLDYSGLWTSSFTSITPNVSFYDDYGNKIGTVDMMTTKRRVKLAYIPILNAKVLELPVGNNGRYRMLLQTNLGKKSIRDTIDVFQSTLIVDIFDRLLQSIIPLEISIPVFSMTSDLRLRSALENLEIRKMWKENKGWSPNPVEFFQRVSLDIKSEGLSPAPGSSSSILTSVGGAATGLATAVGREFTANRPFMFALLDATTRTCLFSGAFSKPNL |
| Px010990 | PxSPI9 | MYVTKYGVLLVAAACWAYPQDVPESALGKVIDRASMKVLKEAFTLEAGKNVVSSPLGMLLLLSQYSAGLGDGALKQEITSLLSTKGYSELVSDYGKLSNTFSSLNPNFLSLKNKIYVAEGFTLDDEFSASSRGTYRSEIENLKFTEPSKAAAVINEWAEKETHGNIKNAVSPDALSPDVAVAMFNVIYFKGQWEYPFTKEETKEKDFHLSKDKKVKKSIMHVMKNFRYHDSELLGAQLAELPYQEEGFRMIVALPHEVDGLSSVVEKVAQNGLLGEASKLEYTRGGVCLELPKFNVDSDLDFEDILKKVGLSHLFTEPATKLVKNQSVVVSKAFQKAFIKVDEEGSTAGAFSGMIAIPMSSNYVVPDPVMFHVDRPFFYAILHQDVIVFAGTYTH |
| Px002882 | PxSPI10 | LGEDIAQFTELCNELAFRYWHSMTENLEKRRSFVLSPYSITSMLAMMFMGARGATSGEMNDILKLDDMVTFNPHFTLKNISDSIETTPEAGVVVSAFVRQLYSDRNKGKILSFYKERAQHFYNGHVEEINFKLISDIIRRRTNLLVKRHSWNRVPEYMKTNSITMQPPLAALSTNVFQTDCSGTSVDGRDGEMYFVVSPNVRQRRLVPVPAVVYKRGFLAGYDPSLDATAAALGNTNSIVSTLFLMPGQQGNVTMGLKELFDPEHADLGGLNGPSKDLYLSDMIQLTTFVTCGEGSIGEQHHIEEYPEKLDANRRRRTSRWAASWEEPRDYQRAFHDPHDVGDAMHLPLHLRPRQARVPTRSAQPARLKFDRPFLFFVRHNPSGMILYVGRYNPRLLP |
| Px015089 | PxSPI11 | MKVLICVVFLASQLCTLNGQYSQKDKSLNSRLNYFDTDLLRYAAEDRPGNIMLSPASIKSVLAMILEGAGGKTEAEIRSALRLSPNKRDYREQLHLYLQSLQAHTPGTQLENANAVFVSTKLGRQVKQEYDQMIRKVYMSELTPLDFLRTDKSAQHINKWVKEKTHGLIPEVIQPGNINPTTDILLLNALYFKSNWARSFFEGSRSSCFYTQGQCRKVAMMSQVDEVNYAFVDNLRAHAVELPYVHGKYSMIVLVPAEREGLGTLIRDLPYMSLPQISDLMQRTEVDLSLPRFQVTYEEDLVPILKKMKINEAFTKDSNLTGVFQGESAQINALFHKVYIDVNEQGTEAAASTGAVVVPLISDNVQLLVDRPFVFFIRDNELGSVLFEGKIEEPTRWEDGPRGS |
| Px015087 | PxSPI12 | MTNFGLNLLKNLAQPGNVVVSPYSIAVCLALLQQGARGDAEYQISRVIGMRADEAAYSYKRLTDTVKNRSSKNILQVATSIFTAMNAFQLNQNFSNVAKQNFDSEVNPINFERPAYAVSRINSWVAVKTKNFIKQLLSTDSISAATQLVLANAVYFKGTWQTRFSSEMTQPMEFYLSNGQRKMVPFMRQITNFQAGLDKYIGAQVVVMPFEGAQYSLMLILPEGGSSLDTVLSKIDEVRLLEYSKLESREVRMFLPRFTIRTDTQLRNVLQKMGLTRIFEARSDLSGIEATGTQTPKVSTAVHTAVLSIDEQGGTAAAATGFAAVALSGDDDTIYFKADRPFLAILWDSQQAVPLFMTKIEDPS |
| Px011577 | PxSPI13 | MRVNINEKLKFTNTMDWSIQIVLVCGAICLTGSDPVPQVEETQPPLSHCINNFGYRLLLDLTTRSEGQNVVLGPTSVAEISANRQNHEQFGELLQGLEVGNSTSTVYADAIFVDASAQVREIYREYLQRVYHGDALTTDFTNQSLAKKAINEWVKAQTLGRIEEFVRNPLSRETRVVLLSALAFSGQWAQPFMPEYTKKLSFQKATGETVLTDLMLNQGRFNYILSEEHGCHMVALPYNDTTTTMYILAPRSFAKKKKMDLPTLLTGLNWTTIENLISQMRNETTVIRLPKMQLKGHADLKSSLKSLGVKSIFEVGKANFALLIQGKSRLQANKSDELLTRINDPDDYEDDRMLKEQLNKLPNPGVYVDTVMHEVEMTIDEYGTEAVAVTAGIVARTANLFFADSPFFFFIKNELTGLVTFSSAIFDPTIAV |
| Px003822 | PxSPI14 | MWFKWWFKSFTWFAAIAMVVGKQVPCNHENAMLLSKGPSYRFAMEVLHAMDFENDGHFVFSPLASWLQLMNVLEGASGYTQAEILKATGHHPNICYRRKLREVLNGLLGNESRTEYKMNSLIIVDELLGVKKEFKEEVTKTKTLDVLALDFVHPNASANIANQLIENKTGGIFKDMVFEDDFEDTYFLTYDFANYKSAWMQSFDPLKTKMEVFYGPDGKAIGYVRMMNTVGPFYTASLPQLNLNILELTSDKNNQVSMLVFLPQHPSPDSVFSNLNKTTMAGIFNSLKKEAPKTVAVSLPKFEIETWSSPNEMMFDMGMKSMFYLDNAQLGGISKFKINVSMMLQFAAIEVTEGGVKAASVTAGRGKRDLVQFKANHPFGFAIVDKKTEFVLFAGMYSGPRRADRLDETD |
| Px003823 | PxSPI15 | MHGVQKDGTIDLQIPLIPLAASQCSVEKAKPGLRRAIYDFATELTTRVAIDSENHFVMSPLSLWTLLAAVSEGADSETLVQLKRALRLHPRKCFNSKYYEILRGVVESQGETILERGGAVVIDEGFSVKKAFKSRIDAAGVCKVLTTQFAAAEDAASAINSYVSRATHGAVEEITSPEDLDGKSLLLLDTIYFKGKWTVPFSTADTEVSTFYDEFGGAAGDVNLMFITGDFNVAQIDKIQAKVLELPYAGGKYSMLVFLPYTDVPLVQVIEALNLINLQSIFHLYEKKPARTVMVQLPRFKIESNLDFLVELLQDMGLTHMFDEQASFPLISDQALYVSTLLQKANIEVTEEGTVASAVSEAGFSSRLMPEQFVAQKPFLYMIVDKVNEVVLFTGAYSKPSVY |
| Px007272 | PxSPI16 | MITIVYILGFLVAVGNCDIDYSDRPRNFSIELVHFTAQQTGGHVVVSPFGIWTLMNGIALGATGGTYEEIRRAFFLPRNERKMIIGYGNLTKIVLNPTTPEVSLTSKNFMFLDRYFQINDEFLSVIQTDFQATVSRLDFNDPKEAAKTANGYIQNSGARVSNVLTSEDFAESRMILTNVISFKGLWGLPFNKTDTIEEKFYNERREPIGRVNMMYQRGKFPYGFFKELNGHVLELPYGSDGKYSMLILLPFGGNTTTDMYMRLETISLKDIFRILQEDAENYGVEDVDVRIPRFKVSSNIVMNKPLNDMGVYKVFDPLKSSFERVTKEPLFVSAIVHKAEIEVTESGTVAAASSTADFADRISTPSFAGNRPFIYFVMEKSTTTMIFGGIYSKPAVY |
| Px014010 | PxSPI17 | MKTMIFNRIAILLLVLVYLSEEVVGSDDRSVNIVDRTLVPTTPNVNTKGLTRSSGSDEVNGKGSIRRDLALMAALESPEENLVISPVSLVVLLSQFLLGATGKTETELVQAVGMDKTQLSRDLKPLLAVLNQKVENMTIHSVTKLFVDQSIKLLETFESKNKLFLDNSVKKIEFSKSVESAKIINDWVSEETNDKIKNMVTPKEVANAVMIGLNAIYFKGVWQTKFNDAKPGHFTTNNGVATVPMMSVTDFFEYMNSTDLDAQMIRIPYQGNVSSMVIILPSGKKDLPSLLRKLRQSPELLNKNLAALRKDLYPSNYKQLDLTMPKFKIENEINLKPLCKKLGLEAIFESNTGLSEMSASPLSANAIKQKAFLSVTETGTEATAATEIDGVSSSAPSPFEMNVDHPFCYIIQTQNEIIFGGIVNSP |
| Px015090 | PxSPI18 | MLTLTDDAPLAHSTNTGTPKPPTIDESVLQNVFGSPQSFYSQAVPVVPENGTLVDPDYWDSEDIYRPSSLTYDLFDLSITKRVAKSSPDNFLMSPLGLKLALAILTEAATGTTQAELVSVLNFDKDRNTVRHKFASIVSSLKTKSPQYILDLGSRIYVDNTAKTRQRFAAIAEQFYNTELQNIDLSKPGPAAKDINAWVSEKTQGRISQLVTEADLASAVVLILNTLYFRGTWRHQFAPNETAAAPFFVTPSVQKQAHFMKVQNSFYFAESMKFDAKILRMPYLGRKYSMNIIVPNSLSGLPRVVENLSELRQEMTFLTEKVVDVTIPRFQFDYTTHFENILKELGVREAFEDTASFPGISRGQTLRQRLKVSNVLQKAGIEVNELGSTAYSATGLLKVCSCAPRLESFLQYCVQAEPLKGNLIDADYWDDDEPSTYEPPT |
|  | PxSPI19 | MPYDFFDLGLIQRKAEGVGNFLISPFGVKTAIGLLAEASTDTTQVELINALGFQTDRYANRKKYSEINNFMKMNSTANVMDAGSRIYLNTSNTPRARFAAIAQEFYGTDLVNLDYSNPTLASQDINSWVSGVTKGRIPDLITEAEFRDASLMVMNTMFFHGTWRHQFDSRRATARVFYYTARTSKILPFMRVQNTFYYADSLVFDAKILRMPFLSNKLAMNIIIPNSSTGFARIMDNLQELHKEMGFLRERLGDVNLPKFQFADITSYRQILSELGVREAFGATASFPGVSRGQQLNGRWKLSTVLQKVGIEVNEFGPISRYKSDETSETSATGAVLEVIANRPFFFFVEYEPLRQVLLAGRLADPAPPPARVPIPLALPWN |
| Px004226 | PxSPI20 | MVSTTPSYTEITETSKQYEVDQLSSNQLKEKYFKAVEIFDRNLYQVTFAKLSNSSEEGRQENGISFVQSGLLLHLVLTALSPAVDQEIRTEIEDVTGYAPTEEERTLILESVLSWLPSSSASLIFRRAARLVAAPGVPVGEGLGLRVDRLNGTEGPEELARVLNDMAIEIQYLEPGLSLLLVVPREPSLRSLAQRLAAEGISPVLQRLQERRLAVVMPLYTLRMTLLLPNKLESMGVSGLVSGRGGCGALRLSHAVQRIMFWAEAGRHAYKDDGIEWDAPAEREVAVDRPYAFYVRWRNVTLMNGHFVL |
| Px002346 | PxSPI21 | MHKGSIRESQLSRDLKPLLAVLNQKVENMTIHSVTKLFVDQSIKLLETFESKNKLYLDSSVKKIEFSKSVESAKVINDWVSEETNDKIKNMGVWQTKFNDAKPGHFTTNNGVATVPMMSVTDFFEYTNSTDLDAQMIRIPYQGNVSSMVIILPSGKKDLPSLLRKLRQSPELLNKNLAALRKDLYPSNYKQLDLTMPKFKIENEINLKPLCKKLGFEAIFESDTGLSEMSASPLSANAIKQKAFLSVTETGTEATAATEIDGVSSSAPSPFEMNVDHPFCYIIQTQNEIIFGGIVNSP |
| Px016549 | PxSPI22 | MSRTAEQYPEFMKAVFDIPKSEISVSTNSYIFYDNVVPVERDFMGTLERDFKTVVNQISPVNWTATDVIVNDIKKSHTHAVNVINLSNLQGAVTMRAINFISFEGLWGVPFDKRDTAVNNFYNDQGELIGEVNMMHQKGPFQFTDIKELEAVLIDLPFANNDKYSMLVILPYDKRNTTAIYRKFLTYSIPDIYKSLKDDLEAYGEEIVDLKIPRFRITDEINMEKPLNSMGVYSLFDKSDFHRAVRLPVQISGFTQSVNIEITESGSGLGATKPKEQPIRSLISNIVVDRPFIFFIMEKSTLSILIGGIYSKPEKY |
| Px003869 | PxSPI23 | MATGYLILLLVAACWAYPQDVPESALGKVIDRASMKVLKEAFTLEAGKNVVSSPLGMLLLLSQYSAGLGDGALKQEITSLLSTKGYSELVSDYGKLSNTFSSLNPNFLSLKNKIYVAEGFTLDDEFSASSRGTYRSEIENLKFTEPSKAAAVINEWAEKETHGNIKNAVSPDALSPDVAVAMFNVIYFKVLFISRANGSTRSRRXETKEKDFHLSKDKKVKKSIMHVMKNFRYHDSELLGAQLAELPYQEEGFRMIVALPHEVDGLSSVVEKVAQNGLLGEASKLEYTRGGVCLELPKFNVDSDLDFEDILKKVGLSHLFTEPATKLVKNQSVVVSKAFQKAFIKVDEEGSTAGAFSGAIAVLTSLQIPEPVPIKFLVDRPFFYAILHKDIVLFTGTYIH |
| Px007765 | PxSPI24 | MNTWNEKHIKMSPFFIFMLLIQLTTTQSEETIMNPYAKEVSLFSAKFMNFAYVPGKNLVSSPVMVELLLGLLALGSKDPSQLIDVMHLGSNKDQLKVAFKKVSGAMQNIKGVTFYAANKLYVKECDSVVMPAFKKEAVHAFWAEVEKVDFDMGPVAADIISTWVEKITKGLIKDIIDPSTIESNTQAVMVNGLYFKGKWEKEFDPLDTETQTFHVSSSTAKNVQMMSQEETFKYGNCSALSAQLLELNYSKGKASMLIVLPKEVEGLKNVLRLLSDGYDLVSERGKMKPTRVQVRIPKFAIESDLDLSVLLPKMGVSSIFSSSAGITQILGNNESLEVSKGVQKAFLEVDEEGSKAASSSGMSLRSRRSVPEPPEQFVADRPALYIILLDDLPVLSAIYHPEPDTRDEL |
| Px017058 | PxSPI25 | MKVSICVAFLASQLCTLNGQYSQKDKSLNSRLNYFDTDLLRYAAEDRPGNIMLSPASIKSVLAMILEGAGGKTEAEIRSALRLSPNKRDYREQLHLYLQSLQAHTPGTQLENANAVFVSTKLGRQVKQEYDQMIRKVYMSELTPLDFLRTDKSAQHINKWVKEKTHGLIPEVIQPGNINPTTDILLLNALYFKSNWARSFFEGSRSSCFYTQGQCRKVAMMSQVDEVNYAFVDNLRAHAVELPYVHGKYSMIVLVPAEREGLGTLIRDLPYMSLPQISDLMQRTEVDLSLPRFQVTYEEDLVPILKKMKINEAFTKDSNLTGVFQGESAQINALFHKVYIDVNEQGTEAAASTGAVVVPLISDNVQLLVDRPFVFFIRDNELGSVLFEGKIEEPTRWEDSPRGRKPAKAPQSSGNRKQGRK |
| Px002767 | PxSPI26 | MRDTTVTILKLLLSGSLAEEGDQVDPYRAGEATQPQCRENEEPTNCYNSCVEDSCSNRLSPNRPCPRNCEGNRCKCKKGFYRNENGDCVGVEQCGAPKCRDTEVYTQCGSACPPTCETRDSGPTICTQQCVEQCACKKGLIKNQYGTCVKPEDCPSQQQCPAGQEFTTCTRQKCVKTCAHVLHRPLCPRQSAGKCYSPGCICPGELVEGPDGTCIPKEQCPTFECQENEVYSNCSNTCPPQTCNSILELYNCPDQPPKDCKPGCLCKPGYLRNGTTSDACILSDFCFGEPVCGDNQSYVQNKTICQASCDSEVVVCDTVTGCACNDGFIPDGQGQCVKKEDCPTPDCKYQDTEYYSNCGTACELTCENYKNPPEVCTQQCVDKCFCYKGFVRAPDGSCVKPENCPPNTCPSHERPADCYNACRENSCSTRLQTKKDCDYRCEHDKCTCQDDYYRHSNGSCVTPLECSPPKCKEDEVYNNCATACPITCANRNNPPEVCITLCVEECSCKPGLIRDQDGSCVLPEDCSISPPTCAANEEPTDCDNDCIPTCKTRSRSAKQLCSADCKPGCKCKEGYLKNDNNVCVPEEECSKPPNPPTEVCGQNEVYEKCKIHCPPQLCSTVYAPINCFAPDYCTEPGCNCKEGFLRNGTTTDPCIRREDCPKPTCPDKEVFSECGSRCELTCDNYGNPQKCSDQCVTGCFCKPGLVRNSDGLCVKPRECPPPLCGKDEVYNRCAPKEELTCANCKNPPRNNDHQCVEKCVCKDGLVRGPNGKCIKSEKCPQIQCGKNEVFAECRTKCPPQNCLSLYAQFRCQENIPCEPGCNCKEGYKRNGNFSAPCIPVKDCPKPGETEPPQIKCGRNEVYTNCTIKCPPQTCNALYAKFKCASDIPCEPGCNCREGYRRNGNENAPCIPVKECPNTNVAPPPQQQCGHNEVYTKCKKSCPPESCLSKFIKVSCLEDQACSGGCKCRKGYLRNGTAPDSPCIRKRHCQAPEPPQLPQCGENQVFSNCSSACLKTCRNRFLHPVCTRQCKPACVCADGYLKHDDGTCVREKQCSPIICNDTEEYNECGTACEDTCTPTQCKENEVFSDCKFVCPPQTCDSLYTSYVPCDDCVPGCNCVDGYLRNGTANDLCIPSDLCPPVYEPSSLDSSSWQPPSVLSDSDVHLSDIHLSLQKIQYELKSLDAHKGPGPDGLPAIFLKNTSVSVCTPLYIIYNKCLSEGTCPASWKMSNIVPVHKSGSRSEVDKYRPISIMSALSKLLERLIHSVIYPALHKIILPEQHGFVKKRLTTTNLILFTSFIFESIDNREQVDAVYTDFQKAFDNGIFYDGFAHISRIEARELWSMDFPPILNE |
| Px006736 | PxSPI27 | MTQAPPQCSSGEEYKTCGTACEETCQNYENTCKFCNFKCVEGCFCKKGLVRDSNGVCVTPEKCPKPICKGPNEYYDKCPNTCPPQECSADRKSYDCSNTKNCCAPQCRCVKGHLRDNKGICIPEEKCPTPSNTSSPSGGPNNATTGGSTSPNNGTSSSSVTVSPTSSDSSSSSTNTTVSTSPSSNGSTASPGVCANNEIFVDCTDIKCYQTCDDVNKRPTCPTIENECSKPGCICIDGYVKDQNGNCIDADDCPIPNQPICPQNERYTLCNLTKCYQTCEDVQRKPECPEIDSLCEKGGPACVCIDGYVRTANGTCIDADDCPVKPIPECPINEEYTTCRLDKCYATCDDVINKPDCKASSGCFRPGCVCIEGYARARNGSCIDADECPRNPPPECPTNEEYTTCVDTKCYQTCDDVTQESDCEAASDCYRPGCICIDGYARARNGTCIDADECVVTATPTGTTTQSTSSGQTNSSGGSSNSSGSSTTAPGGNTTTPEDCPVNEIFVECTLTRCYQTCEDVISGDECEEVPYYCYEPGCVCKDGYVRADNGTCIDADECSQPPTPICGPNEQYVKCNLTKCYQTCNDVLTQPDCPYIDEDCVQPACVCIDGWVRGPDGNCIDADDCSTGQSSPNGNNTSTTTGPDQTTPQCPNDEEYNSCGPYCEKSCSNYNESINCPTLCVAGCFCQFGLVRANDGRCVKPEECTPNPIPACPVNQKYSPCTLTKCYQTCDDVINESDCPEVPPNCRRPGCVCIDGYVKPRNGSCVPADKCLQFVIMYSSISHISHVPAPNPGPKCLTNEQYTNCTLAKCKQTCDNVVFKSDCPIVPSNCSRPGCECIDGYARARNGTCIKNENCPTPPPPVCPTNEVYSNCTATKCYKNCYELLTTPTCPPVPDPCNKPGCVCINDYLRAPNGTCVPRADCPPDPIPQCDDDEEYTNCTTSKCLKTCDDLLKQPTCATPPANCSRPGCVCAKDTVRARNGTCVPVDQCPPPPKPQCPRNEILSNCTLNKCYKTCSDLKKKPLCPTAPTECQKRGCICKDDWLRAKNGTCIPQDQCGKLL |
| Px006737 | PxSPI28 | MQLFLLFFTAYFAVCANATAPIVNDPSCETYNDSDLSSSASSSEASSNSEGSENNGSVTQESSQSSSASSFAAATASSQSSGSSDDGSYAYASSSSSAASSSSAASSSSSSTVTTGNQNNQNRCGINEVPDNCYNPCYEKNCANRLKPSRLCPLYCMTDGCSCREGFYRDDNRNCVPLQECTPPKCGTNEVYTNCGSACETNCTNIDNPPQACTLQCVEKCECLEGFIRNSDGNCVKKEECSSVQCGPNEESNGCYNSCLEKSCASRLNPPEVCPLYCEYGKCSCTDGYYRDDNGDCVQPLDCTPPRCGLNEVYTNCGSACEITCANVDNPPRVCTLQCVERCQCAEGFIRNSDGNCVRREECLPTIQCDENEEPTDCYNSCLEDSCANRLQPRRFCAQYCETNKCTCKRGLFRNDDGVCVPVDQCSTPKCFEGEEYTECGSACEATCNNLNDTSCTKQCVEKCSCRKGLVRSDDGSCILPENCSLQQQCKENEVYSDCKITHPPQTCDSIYSQYQPCEDCPCNPGCNCKDGYLRNGTMDDACIPSESCPPPTPVCSDNEKFNSCGTACELTCDNYKNPPNVCTKECVAQCFCQEGLVRASDGSCILPEYCEIQNQCQENEVFSDCKITHPPQTCDSIYTQYQPCEDCPCNPGCNCRDGYLRNGTMDDACIPSELCPTPQPSCPDNQEFNSCGSACELTCDNYSNPPGFCTFQCVAKCFCKDGLVRASDGSCVRPQDCSLQPQCKENEVYSDCKIIRPPQTCDSIYTQYQPCEDCQCSPGCNCKDGYLRNGTMDDLCIPSESCPPPSPSCADKEQYKSCGTACELTCDNYKDAPLDCTKQCVEKCFCQDGLVRASDGTCILPENCPTTQCKENEVYSDCKFVCPPQTCDSLYTSYQPCDDCVPGCNCVDGYLRNGTANDICIPSDLCPPAVQQPPTCPEGQEFNSCGSACELTCDNYSNPPGFCTYQCVAKCFCKDGLVRASDGSCVLPENCSAKPQCRENEEPTNCYNSCVEDSCSNRLTPNRPCPRNCEGNRCKCKKGFYRNENGDCVGVEQCGAPKCRDSEVYTQCGSACPPTCETRDSGPTICTQQCVEQCACKKGLIKNQYGTCVKPEDCPSQQQCPAGQEFTTCTRQKCVKTCAHVLHRPLCPRQSAGKCYSPGCICPGELVEGPDGTCIPKEQCPTLECQENEVYSNCSNTCPPQTCNSILELYNCPDQPPKDCKPGCLCKPGYMRNGTTSDACILSDFCFAAPVTCPANEVYYKCKDERCYTDCNQLYTSRACPSLSADCYNPGCLCKEGWYRNSSGVCVTEEQCAPNTCPSHERPADCYNACRENSCTTRLQTKKDCDYRCEHDKCTCQDDYYRHSNGSCVTPLECSPPKCKEDEVYNNCATACPITCANRNNPPEVCITLCVEECSCKPGLIRDQNGSCVLPEDCSISPPTCAANEEPTDCDNDCLPTCKTRFRTDQQLCSADCKPGCKCKEGFFKNDNNVCVPEEECSPPQCSKDEVYSRCAPKEELTCANCKNPPRNNDHQCTEKCVCKDGLVRGPNGKCIKSEKCPQIQCGKNEVFAECRTKCPPQNCLSLYAQFRCQENIPCEPGCNCKEGYKRNGNFSAPCIPVKDCPKPGETEPPQIKCGRNEVYTNCTIKCPPQTCNALYAKFKCASDIPCEPGCNCREGYRRNGNENAPCIPVKECPNTNVAPPPPQQCGANEVYTKCKKSCPPESCLSKFIKVSCLEDQACSGGCKCRKGYRRNGTAPDSPCIRKRHCQAPEPIPLCGPNEVFVRDADVKQSTCSNPQGDSIPSYTGCICQLGYVKNDNDECVDPKQCAPQLPQCGENQVFSNCSSACLKTCRNRFLHPVCTRQCKPACVCADGYLKHDDGSCVRVEQCSPPQCGPNEHFEPCPPNCPPNTCFANRARYDCDKFSKECIPQCYCDEGYQRNKNNVCVKTENCD |
| Px006739 | PxSPI29 | MTKTNEFPGAQPVSQCPGINEEPTTCVPCEESCRYRLKRSDIACSSVCNAGCRCVDGFYRLDSGECVPVDQCSPPQCYENEYHDTCGPYCKESCATMNNTCQVCLSGCVEGCFCQPGFIRDRYGLNCVTPDQCPQPECDRFEHFETCPNVCPLETCRAKLEAEDCSSLQCCEPQCRCDEGYLRNDSGECIPEEYCEVDY |
| Px006740 | PxSPI30 | MYDATRCCVKSRQNLTKACGINEVETSCDDGRIATCKYRFSVNPPCVGDNSSGVTCVCAPNYYKHDNGTCVPANECSPPQCGKYEVAKIGEQCGPACEGTCQNRGKPGACSTVCTDGCFCKPNFVREVDGSCVPLPSCKNIIYGSASAVSSAQSGDGNSIAQSASSNDDTNSQTLASSDGSGFSQSEASSSNNGDSSADAFVPNDGSEYSQIEASPSSDDSESSQAVASNFNDGSSYSQTVVSNSNNGDSFSQAVASNSNDGSGGSQAAASSYNDGSGVYYATASSSNENNDSSQAGAYASNDDGSNFSQAIASSSNDNGSAQAFASSSNDGPDTSQADASSYNDGSGVSYAAASSSNENNASPQVVSYTSNDDGSSFSQATASSTNENNKSPQAVAYASNDNESSFSQASSTNDNGYSQASNSDDGSYTSQAYASSSDDGSGVSHAAASSSNENHNTPQAVSYASNNEDSSFSQAATSSSSNDGSGVSYASASSSNDNNNSPQAVAYASNDDGSSFSQATASSTNDNSYSQASSSNDGSYTSQADASSYNDGSDGSYAAASTSNENSDTPQAVAYASNDDTSSQTLASTSSDGSGFSQAEASSTNNNNDSPQAIAYASNDDGSSFSQASSSSTNGDGSAQAISSSSASNGDSGYSQAVVANSNNDNGSSQAAASSSSTNYGPRYTQAVASSQSSGGSAQAIALSSSGQENVCGANEIPSDCVSECDVDCKTRDYPSNQVCALGCKPGCKCIPGYLRNDEGYCVPVYQCFCYGRNEVPTNCMPCVDSCRLRLKMNQTACAAACYSGCTCRRGYWRHDNGECVRVDQCSEPLCYDDEVYDPCGPSCEETCDNSIAPDDVCMQQCNEGCFCFPGTVRSKNGSCVSPENC |
| Px007753 | PxSPI31 | MLKRVDILFIVLFGAFASVSSLYTVQEECAPNEEYLICGSACPFNCTDPEGPVVCEEDCVEGCFCKNGYLRDVNGSCVPADQCVGETPSICSPNEEFLFCGSACPLTCAQPERAPCGLACSVGCFCKAGYVRDEKSNKCVTLDQCPVACHVGCFCKAGYVRDEKSNKCLTLDQCPVEQCLNSNETYAICTASCQPTCSDPEPLCLQVCSSGCVCNEGLLRDDSGVWVRAEQCPPSNSTDAGLVGKYYDVINKILHLSTA |
| Px007757 | PxSPI32 | MKTNAICGLYLLLCVAVDIVFGQVASLCRPHERFLSCGGCQRSCRHPAPACEAVCRQGCYCPVDLVRNDDGECVRLSECPTFSDAVTPDTTEPRPRGTVCPPNEEYHYCSPCNRTCAAPDPVCPAQCARGCFCRGDLVRNESGVCVERRECPERQLKPAPNIICDLTQQFQTCGCEATCFDQNPSCDQKDCSAGCFCRPGLVRAPGGKCVKLTHCSQAPPLRKLKRRPRWLH |
| Px010825 | PxSPI33 | MEKLALNRKTYLWKFIYFHKDAKQQDLTMPSSSTEECAANEEYLICGSACPFNCTDPEGPVVCEEDCVEGCFCKNGFLRDVNGSCVPADQCVGDTPSICSPNEEFLSCGSACPLTCAQPERAPCGLACSVGCFCKAGYVRDEKSNKCVTLDQCPVGLLHGATLYHAAWAASARLATSGMRRAILVSRWISALLPERAPCGLACSVGCFCKAGYVRDEKSNKCVTLDQCPVEQCLNSNETYAICTASCQPTCSDPEPLCLQVCTSGCVCNEGLLRDDSGVCVRAEQCPPSNSTDAGLVGKYYDVINKILHLSTA |
| Px011976 | PxSPI34 | MAEQQRNWLWTCSVVLFLLNYVFSTSVYQPPPPVDQHGCTANQVYVSCAFNCPSDYCPRSDGPVPQCSPPYPCQPGCRCKVNHRIHPDGQCRRSDLCPPIRCTRFNEVYNSCTDVCTRESCADRNNTSQCNTLLLNCEPRCACRKGTWRNRHGICVPANRCT |
| Px003939 | PxSPI35 | MWQLCMVLAAAAAVDAQDCALRPGDSAAPKSPGDNFYRLILNGDLERYAPGQRYVVTLVGARTHSVVQQFTRFKLIVDPLDPAAPRLPSKQGQFQLFADTLTKFDEDCTNAVVEADDLPKTEVAVMWKAPPAGSGCVLLKAMVYENGRRWFAEDGQLTRRVCEDTSVSMPDCCACDDAKYKMVFEGLWSPQTHPKDFPKQALWLTHFSDVIGATHPKNFTFWGEGVIATDGFRSLAEWGSVGAVEAELRQQGGLLRSIVKAQGLWHPRVNSNTSAVFTVDRKRPYLSLASMFGPSPDWVVGVSGLDLCLKDCSWVESKVIDLFPYDAGTDSGLSYMSANSETVPRERMYRITPMYPEDPRAPFYNPQATEIPPMARLYLTREKLISRSCDEEVLQMMVAEEQENTQTADRPECAVSAWGAWSACSVSCGKGLRMRTRQYRLPQKALMFACDRQLVSKEMCVAAVPECEGDSAAAAALESPPVEDLSGVCATSAWGPWSECSVTCGIGFNTRRRSFLNHMGLKKCPLVATEENRKCMEPECVEKESDISDPSCPTTEWAAWSPCSASCGRGVRQVTNIHSLLLLRTRLLLVPPERQQECSSKVELLQQRPCAERDDCSIDMATAKQICMEAPAQGPCRGLYRRWAFAAEKGMCVPFQYGGCRGNRNNFISEQDCMDTCRVVLGGAAPAPPSPASPPRPAASPGYPALSLNSIQPPPTLNGSSRDCEVGPWGEWGRCSVSCGVGYQERTRAVLVSSTFRAF |
| Px011924 | PxSPI36 | MADENPHRIDPTCFLPPEPGRCKARKRRYYFDPTTGSCSSFNYGGCGGNGNRFKTINHCKNKCLPSTRSPEYCQLPFDYGDCDVGEGVKWYYDAASALCLEEIYSGCGGNGNIFDTEEHCYDTCAELKPTNELPKRKPRLSEAGVSAILGSKRLQGILENIFRNKPPKNLKVVFNLYED |
| Px011925 | PxSPI37 | MSYHSIDPTSCLLPPEPGRCKARKRRYYFDPTTGSCSSFNYGGCGGNGNRFKTINHCKNKCLPSTRSPEYCQLPFDYGDCDVGEGVKWYYDAASALCLEEIYSGCGGNGNIFDTEEHCYDTCAELKPTNEQPKRKPRRVF |
| Px011926 | PxSPI38 | MLFLQVIFLFGYLTEHTSMAYLYPEYPDRVGSIKDIDPACLLPPETGMCKARMPRYYFDPKTLSCSSKFIYGGCHGNGNRFLTELQCKNKCFPARASITDIDPACILPPETGMCKARTPRYYFDPTTGSCSSFIYGGCGGNGNRFLTEEQCKNKCLNKSLARTSTPEYCQLSFDYGDCHSNGLVLSPRWYYDPDTASCKVAMYSGCGGNGNRFDTEEDCYDTCAEPKPTNKLRKPSLRETGVSAILGSEKLQGILENIFRTKTPKNLMIVFDL |
| Px011927 | PxSPI39 | MFLFSFVLFSSCLNVYTKISYNTLTPPVSKAHESADNIDEVAKMCLLNPQRGPCRAGLQMHYFNAKDMNCASTFTWGGCQGNGNRFDTEEECEEQCFRARYRRRPEHCLLNFDYGRCFGEIKRWYYDRVYGDCKETMYSGCGGNANRFGSKAECDQACVKDNRKAPYKPTE |
| Px013236 | PxSPI40 | MQEALCDGEHPDCAPVQPGPEVCTLPLSVGPCRGYSERWFYSPRARACEPFGYTGCGGNGNNFPTHDECAAMCKGVGEGSATTAVPATVPTTVSTEPPAKKLKPLVTKEDNEVMQSDSPAGDAQTSSRVFPLS |
| Px009905 | PxSPI41 | MWQLCIVLAAAAAVGAQDCALRPGDSAAPKSPGDNFYRLILNGDLERYAPGQRYVVTLVGARTHSVVQQFTRFKVIVDPLDPAAPRLPSKQGQFQLFADTLTKFDEDCTNAVVEADDLPKTEVAVMWKAPPAGSGCVLLKAMVYENGRRWFAEDGQLTRRVCEDTSVSMPDCCACDDAKYKMVFEGLWSPQTHPKDFPKQALWLTHFSDVIGATHPKNFTFWGEGVIATDGFRSLAEWGSVGAVEAELRQQGGLLRSIVKAQGLWHPRVNSNTSAVFTVDRKRPYLSLASMFGPSPDWVVGVSGLDLCLKDCSWVESKVIDLFPYDAGTDSGLSYMSANSETVPRERMYRITPMYPEDPRAPFYNPQASEIPPMARLYLTREKLISRSCDEEVLQMMVAEEQENTQTADRPECAVSAWGAWSACSVSCGKGLRMRTRQYRLPQKALMFACDRQLVSKEMCVAAVPECEGDSAAAAALESPPVEDLSGVCATSAWGPWSECSVTCGIGFNTRRRSFLNHMGLKKCPLVPTEENRKCMEPECVEKESDISDPSCPTTEWAAWSPCSASCGRGVRLRTRLLLVPPERQQECSSKVELLQQRPCAEREDCSIDMATAKQICMEAPAQGPCRGLYRRWAFAAEKGMCVPFQYGGCRGNRNNFISEQDCMDTCRVVLGGAAPAPPSPAAPPRPAASAPAYPALSLNSIQPPPTLNGSSRDCEVGPWGEWGRCSVSCGVGYQERTRAVLRPASPGGAPCPERLSRRRRCARHC |
| Px012095 | PxSPI42 | MVQQSKVILNDIFSGSVLRHRSRHRRQGAGLYLAASYVLPGGEGGGAWGPWGEVSPCSRTCGGGVASQKRICLEIGPDGQPRCAGGDTKYFSCETHDCPAGAADFRSEQCAEYNNVTFRGVTYNWIPYTKAPNPCELNCRPRGERFYYRQARQVVDGTRCNEESFDVCVNGECQPVGCDMMLGSNAKEDKCRECRGNGTNCHTANGRIDNQDLTQGYNDVLLIPQGATNIYIAEVRASNNYLALRSKQNNNYYLNGDYHIDFPRSISIAGALWQYERSQQGFPAPDKLRCLGPTNEPLYLSLLLQDVNVGIEYEYSIPKSIAPPPNQQYNWVFDEFTPCSASCGGGIQYRNVTCRSREDLDVVDSSLCDEGLRPATNQTCANHACAARWVEGPWSPCSKPCGEAGTRSRAVYCEKVISNGRGSVVDDSECFNQLGPKPELYQECNKNSACPSWFTGPWKACDKLCGEGKQTRQVVCHQKTNGKVEVFPDKECADEKPPSEQSCMIHPCEGVDWVVSDWVGCDSCLSKERTRQAECATKDRTVVNSTFCSYHPMPVLKEDCDRTKLPPCTVQWYATQWSNCSVECGHGVKVRRIFCGLFDGTAVIKVDDARCSEEEKYNETTPCEIPDEKCPAKWFGGPWSECTKECGGGEQFRHTMCLKGSLEASDCDPEAVLDSSQACNTQACNKGDTYLKLILNKNLFIVTDEIIPVDSKSTPIMDEDYEDEECEEEEEEYPEVGAEESLEGEEDTASSVSDIMFSDSPSTDITEEGSGMESTLFTSSDTETSDEGSAFGSTTELEESLPTSETVSEGSTELVSEGSTEAGESSTTEAGISGSTETLETDVTSSSSDATEVTGESDSTTESSTDVTGATEESGSTEAGGSTEANTGASDSTDQSTTESGSSEASTESASEATSESSTIEESTTEGSTESSTDGASTTDSGSTESSTSASESSDSATDGSATTEESSTDFAGTTEDAEITESSGATEASASEGSTETEVTESAITEVTESGATDVTGSEATEETGGSTVTESTEETGSTVTEETGSTVTEETEETGGSTATEETGSTVTEESVATESEGSTVTEETEATSELSSESTTPWDWYSTVEGTTKHKVCKPRKKKAKCKKSKFGCCPDKRTPAAGPFDEGCPNPKTCKESKYGCCPDGVSPAQGEKNEECPQPPCKETLYGCCKSDNKTPAEGNNEEGCPPPPPKYGCCKDGKTEATGPKKKGCPETAPKKGTKGCAASPQGCCSDGKTEATGPNGAGCPCNITEFGCCPDNVSPDHHFQQSANQILSPARGPQMEGCVMSCNTSAYGCCPDGETPAHGPDNEGCCLLYSFGCCPDNYKPAEGPQLQDYQKMLTGCGCQYAKFGCCPDNITIAHGTNKEGCGCQYTQHGCCPDRHTEAAGPNYEGCGCHTYQFGGCPDGTTIARGPNLYGCHGSQSQFGCCGDGQTAAKGPEGAGCDCAASKHGCCPDGQTEATGEKFAGCADIPVNRQASCALKTDRGPCRNYSVSWYYDIEYGGCSRFWYGGCEGNGNRFSSQEECQDVCVHPSPKDACKLPKSKGACQGYNLHWYFDTDRQQCGQFVYGGCLGNDNNFETRDACQDQCEPARSGNQCHLPLERGSCAGSFNRWGYNKEAGRCEQFTWGGCEGNGNRFNSEAACVQKCDPPGALKPECTLPKEPGNCTEKYARWAFSESENRCLPFYFTGCNGNDNNYQTEEECSARCPSAYEQDTCLLPAETGSCADYREKWYFDSAEQRQVRHSITFCKPCRQFYYGGCGGNDNNFNSQIECERRCRQAASTTTTPQPSAPEQFRNEFCFMEVDQGPCHEAEPRWAYDSARGACTQFQYGGCGGNRNNFPSREYCQYYCSPAQDVCQLPMSTGPCEASVMAWFYDASSDSCSQFTYGGCEGNGNRFQSRDECERRCRTGPAAPAPAGTTPTAGAPPPAETTTTAAAPTESIAPQCQSYPEPCTSTGEVWYYDQGRSECVPHQNQQYGAECRNTGVFQSQEACDRSCGAFRGLDVCNSPLDAGPCQAFETKIFFNQTAQACQAFTYGGCHGGPNRFSTVDECEQTCRPRTDGSDVCNMEQDSGSCDGYYIKWFYDRLRGDCGQFIYTGCGGNQNNFETQEACEGRCIYGQLQPTTALPPPTAAQTVQQQVTVTPQATDPKCTTPESLEPCGFNSTVFYYDSTVQDCVAGNIGGCRHPNSYATEEQCQRECGLFRGLDVCRYNLDPGPCKASIPKYFYDPVTGACQIFAYGGCGGGPNRFSTVEECEKICTRGAAPACLEAVDTGREQCEEVPSRRGHFSALDNDCYAFVFSGCGGNGNTFRSYQHCLSDCEPYGINEVTECDAYIEECNQIRCEYGVQSVRTETGCERCLCAAAPSNCDAQARECAEIKCPFGVDKTVGEDNCERCTCKEHLCAGQVCPAGQRCVLDAYRDPVTQESRYNADCRTVNKPGLCPSEQLLATEQACRRECTDDADCRGVGKCCASGCSQLCVAPLASATTPYVPSTTGVPELPQAPQANPQTEPEVMSEENGKATLRCLFHGNPPPKITWQRGKVTIDGTVGRYRLLSDGALEIVQLYRNDSGVYICVANNGLGTARQEVRLQVNVISSNNNSSGPMVPFVSRQYEARGFVLQIRALDLETIGEYACQAYNGLGKPASWAVVVQAYQPDGQHIDSPFLVARESVVRVTPRLAYTEPTTAATTPEPEPEVPVYTVPVSTRLRATQNKFASGSELSIPCEVDGYPTPEVHWTKDGVRLQPSDRVQLSDARLTISQLAPADSGVYGCHAANAFSSHYATLQITVEGLYIPASCYDNPFFANCALIVSGHFCHHKYYSGFCCNSCVRSGQISIEEIQSLMSSKRRK |
| Px006046 | PxSPI43 | MNRQKDPLTKARCDLICVQVVPEDRSRCRSDCYTGVQKPGTCPAADSAGWAAACVAACDKDSQCDGTKRCCRHECGASCQEPSDLTTVPGLPGLPIMGEIQEKRRSAVLTWSDGVGDLARAVPGKVLYIVEEQHHLGPKYEEERLENWNFVLRSNKTRVSIRDLLKPGRWYRFRVAAVSAEGTRGYSAPSAPFTPRRPPRPPLQPRKLRVVPVDGENQNGTMTIRLMWEEPRSDLPVLRYKVFWSRRLRGVQSQLDSVVVNHQTVAKTQTYYDIKDLEPNSMYFLQVQTISQYGLTKLRSEKAAIFFNTTNVNEHITKKKKPHALKKKSNKKHKKIKGLKLNRIIWQSNHIMANLTWTPSRARENGTPRKFVTI |
| Px006986 | PxSPI44 | MLITNNIKSRLASAESLRRCPAEDSPCPARSGPCGSDQECGQDQICCNTACGPTCVDPLNTGCENIRLSLERISRALAVENSRGGRGLLRGVRAPRCRATDGEFEEIQCDNEIVSSCWCVDPAGFEIAGTRAPAASLVNCTRTAPCAAHTCRMLCPLGFELDAQGCPLCKCRDPCSSITCPGQLSCQLEETPCIRPPCPPVATCKRGRSLQNICPVGEPLLISETNRPFLCGTDPGKPNCPPLYRCLVDQGNDYGVCCPASLEIQKPSTCPAPTLDGECGIPCTHDLECPSVQKCCDGAECGRHCVLPQNVTKCTQQKMLAELLVISEKEGKGYVPQCAADGSFEWRQCSRNGLVCWCVDSDGNKLRGSMGPAASVQCSPSIVQARSGGRSLASECVKALCAGVCEYGYKTGSDGCPTCECDDPCAGYPCPDGEECIRVKASDCVGELCTGYPVCRPLVSYDNPCEVGTPASDSEGNLVACKIDDDCPTEHTCNTTRRNSAAVCCPDPSGYENSSESFVSDMIPSCSEEAEVYCSLNSTVTCSEGSCDKDQVCCNTETCGAICVAPSKMQLQTDDIDATPTMCEYLRDFDEKMEGTVDGMRLALPPPSCTEDGSFTARQCAAGRCWCVDSFGTEIPETRTSNFTALDCAKIREELSCLDLTCRMGCDYGFELGANKCPTCKCREPCADVTCPDGRTCAVVDVACDADYCPPVPACLPRKLGQCPFLVPPNGACEWACRFDAECGPSERCCATGCGTACTKAVHQTACQQRRALAIHDAAESGNPPADSWIPACKEDGSYEDVQCKNSERECWCVDPTGNEIPGTRSANSTLSCSAPSKCPEITCNDIELQCPHSRKLDDKGCPTCECRDPCAEAKCRDDEKCELMKLDCEGESCPPIARCVPAPLCPDGGSPIQTPDGSSNLICGPTAAACPSSHACRFAPHDSRPPVCCPKPRMVCFETMEEGVCKDHEGLNTTRWHFNPEHNRCERFQYHGCAGNHNNFRTKKECMAVCPVLSACERLREKNEAAAQKYGKAYFIPKCDAGGAWENVQCMSHIDVCWCVSTRGEPLKGSLVRGDKPNCNFRQARKWKQRDPLDEKAQADEVLEELIRQMTAYTFEEYEEEDDTEEQSLTTDSSKSEEKKETAEVISTTTEKVKPDEKPKEDTKKPMEKPTKTVFKTKCQLLQQQVENGSDGVRPRCMSDGSFSPRQCSRGRCWCVDAAGQRRPSLGVVPDLPEPDPCELTQIESASLELEIFKVDESIAERSRKLLTTKLARLGARVPVTLAKDREVITVRTMVAGPRAVDIIYQVENWLKEEKLSGVKKGTEGVLSADVIRSEYRLTAPAPAMQQREIVTESTLSSTTAYHTAIIVLAATSAFIISVLCVIVMLYRARLQREPHKAERFLPPAPPVYVLSADEKAELARVLHPPPPVHPQPSNDSMRN |
| Px002484 | PxSPI45 | MSMFTSSVCRYSTQTDRSTDVWSLTEIRLQMHPPIQWVDSARDATCPRICGPALQGEPVCATDGYIYPSLCDMRKKTCGKGVRLAQDQGSCSRAQGSKCDHRCTSEKDPVCGTDGRTYLNRCMLQVEICRVGIGLSHLGACNNISAHRENCPVDCSQAPLDGPICGSDGNVYKNTCQMKLLTCGQGVVRTSKKHCQTTRHCRESCWRVARPTCGSDGKLYANACRMKATNCGKHVFEVPMAFCVSQERTSGGEACATDCSGQKEKLVCGSDENIYRNECEMKMLNCGVSNRKIVKKVDMEKCRSKMNKCLKVKCPVESDPVCGTDANVYTNPCHLKVATCLKGVQLAHFGNCTLLPKLEIDCNEDCDNTVEQPVCGSDGNVYNMRAPGPNXXXXXXXXXXXXXXXCGLRRLPCGQHVVAVSASHCRTTALCHETCPDTPAFVCGSDNRFYKNECIMKKENCGKHVFVVPLKRCLARFQYSGCARVCPPEYDPVCGTDDKTYSNKCFLEMENCRSRSLVQMKHLGTCAEPIAEEPKNYLYR |
| Px003407 | PxSPI46 | MLEPPLASSLLLSALVAFVGGCYLFPSDVPNPCIGVTCGFGEQCRPTADGRSYTCECPASCPSYGEHEGSRPLCASDARDYPAHCEMRRAACETHSNITFKYYGKCVKICRQRRPEPLSWCSYTCECPASCPSYGEHEGSRPLCASDARDHPAHREMRRAACETHSNITFKYYGKCDPCAGVTCPDPEVCQLDENRAPSCRCAESCPLEFSPVCASDGKTYSNECQMNRESCRARKPLRIIFKGQCSSGVNPCAEVECRHGSECRVEGGGAVCACAAPCEPVLRPVCGSDSLTHDSECELRRAACLLGREIQVLHAGACGSNGVCAGRVCPHGGECVSTGGRGVCRCPRCSNEFAPVCGSDGISYGNRCKLQLEACRHRRAVAVLYEGPCNGCENKKCEFYAVCESDGVSEASCVCPKHCDEGTETEEICGSDNKTYSSICSLRDIACRERRHLFVKHMGTCESCASITCPTGTWCARGACACSNPCADAAREPVCASSHQTYQHECALRRAACEARMRGEPPLTVAYYGECSEDREANGTAPNKPKPDLTNDITDEAESSSEPGSAVCARVQCAFEATCAVDAHGQPRCACLFDCAAAAAGAPVCASDMRLYPTLCHMKLEACRRQEDLRLRPLALCRGLEVSWLCYVCYNDRNVFYYIYFIFFSL |
| Px006079 | PxSPI47 | MAGTTIPYTNNSNLEPFKVNLTAACNFNCLCTETDMEPVCGNNGLTYFSPCHAGCAAFSSRENFTNCACVHENPLTIPGVSLNGASASALTAGGGREYSEVTVVPVATAGACNPPCTTIFPFLVLLFFMTFVVAVTQMPLLMIVLRSVSEEERSFALGMQFVIFRLFGYIPSFV |
| Px007758 | PxSPI48 | MIRQLALTVLLAAAAQSAPTVCDHCPADHAPVCGSDQKTYHNECFLTCLTYARAAKIHDGPCGSPSSQVQVANSNGVQVAENTDPCTCTRSYKPVCGSNGETYNNECLLNCVAATDAMARSPVAKIHDGPCLPAGSQVIVADANGVQVAENSADPCTCTRAYKPVCGTNGETYNNECLLNCVASTSSSVALKHVGACGSDHVVPSNKCDCGRDLEPVCGSNGVMYNNKCLFDCALTWNTGMTVVHPSRCGPPPPCCKKP |
| Px009964 | PxSPI49 | MRKKTCGKGVRLAQDQGSCSRAQGSKCDHRCTSEKDPVCGTDGRTYLNRCMLQVEICRVGIGLSHLGACNNISAHRENCPVDCSQAPLDGPICGSDGNVYKNTCQMKLLTCG |
| Px010786 | PxSPI50 | MFVSCPEENYRYLGPGGLNSSLACNSACDCAGMPYNPICGQDSFTYLSPCQAGCQSSMPLDNGSSWLYYNCSCIDNTVRGEKAINTYVLY |
| Px013782 | PxSPI51 | MCNVSPEVLKCAVLLLAAAAHCGPACGVCPFINQPLCGSDGRTYGNQCEFNCAQSKSADLTVQHHGDCEPHGRRGRGVTVVDAAAIPGGVQVAESPLPCNCPRNAKPVCGDNGHTYTNECLLNCQAPSTAVRHAGPCDRGSECDCSKEREPVCGSDTRWYGNRCLLECAQSKDPSVHEAHPRYCIDKDRPYLRDN |
| Px013990 | PxSPI52 | MKSARVRIQKSSAEEKALFSDDRALFILNQFTERAVIRTWGSTAETDLVQACYLHYQPRPPAPEEACEKTFCRWGAECVSLGDGRAHCACPASCPAAPAPVCSTAGRTYRNHCYLRKEACERRLNLRVKHDGECARLNKSDLNILSTLQVVADPCTGISCQAGAKCVVTFGQPECRCPRPPRPRAPPPRKPVCGSDGKEYPSACHLDKHACDSQVNVTIKYHGKCDPCDEHECSDGGECQLSEGRVPVCRCGPPCDLLVRPGSAVCGSDYRDYPSECALRRESCRTRQQLTIAYRGECASAQHPCASVKCGIRERCTLDARGVAVCGCGPDCEEVARPVCGTDGKTYDNPCVLDRSACRANRDIRIAYMGQCGDENPCARAHCPWGGACVARGGAPHCACPVCDSTLAPVCASDHNTYGSECKMRMHACQEGIKEKEISVLYNGTCQTCSDIVCQGGGDCSLDAETGRPVCLCNHNCTEPQEADVVCGSDAQSYPSECELMATACRDQTDLKVAFRGDCALCDGVQCSNGAHCSAGECVCPTECAGVPREPVCGSSMQTYPSECELQRAACRLPPPAALHVIFYGDCKDRLAVVPPIAMTTPAVSTTVSEGEGSTDPAGSTDFGEGPESACRDIRCDFEASCEVGADGYPRCSCLLECPVEEEYFPVCASDFRLYPSLCAMRKEGCQKQLELRLRPLDLCKGMEVRPCGNNKAMLDPTTGLEIDCGNGPHRQDCPIGSYCHITLSAARCCPKNDTKEMQEKKQVHCSETPFSCCADGVTAALGPGEEGCPATASSSCGCNRLGSVSDRCDEAGACACRPGVGGLKCDRCEPGYWGLPRIGSGHTGCRPCGCSAFGSVREDCEQMTGRCVCRAGVQGQKCTICADHRRRLGPNGCSDPESGGVAESCSELSCYFGAVCTERTGGALCECAAAPCADSDSNMLASSLNYYVINCCKVRRPSLSHGGLKFTLFNEIQQVCGSDGKTYESECHLKLQACRTQEDIVVQAFGPCKMYNLGNGAIILTSLEKITLNDYHKVSAKRYHRDGILSVDDMEDVAGQSSGHLKALDLAEDAYIGKVPTNFSR |
| Px013993 | PxSPI53 | MATPFDGGHSENFSDASPLKPVLRPVCGSDSLTHDSECELRRAACLLGREIHALHAAGACGSNGVCAGRVCPHGGECVSTGGRGVCRCPRCSNEFAPVCGSDGISYGNRCKLQLEACRHRRAVAVLYEGPCSESL |
| Px014318 | PxSPI54 | MFFQLGAVLLLAAAAHCGPACGVCPFINQPLCGSDGRTYGNQCEFNCAQSKSADLTVQHHGDCEPHGRRIRGVTVVDAAAIPGGVQVAESPLPCNCPRNAKPVCGDNGQTYTNECLLNCQAPSTAVRHAGPCDRSSECDCSKEREPVCGSDTRWYGNRCLLECAQSKDPSVHEAHPRYCIDKDRPYLRSN |
| Px014322 | PxSPI55 | MFDCAQSRSADLFVLHDGRCEAVLDTRAAIPGGVQVAESPRLCRCPKIKQPVCGDNGHTYSNECLLNCLSPTTSVRHAGPCKRRHPCYCTRERNPVCGSDTRWYGNPCLLECAQSEKKDPNLQEVHPRNCIDKDRP |
| Px014515 | PxSPI56 | MFMLVFLSGWVLQGMFLTYFVSITTTLEKLYKVESKTTGTLLAATEIGQICTALFLTYLAGRGHRPRWIAAMMLVLAVGVVGCAVPHALYGGRLLDVHLDAHRAGPAPTCHTPYNATCDAAHESSNEARSSIAAVMLPWLFVCLLIVGVGQTGIATLGIPYVDDNVGSRQSPLYMDFFTTIKRQLTNDILMWRTASSVLHLMPISGVYSFLPKYLEKEFKLPTHDANLVSGTGGILVMGLGIITSGVVILKLVPTARQVAAWTAITAAIYSFGMIMLMFVSCPEENYRYLGPGGLNSSLACNSACDCAGMPYNPICGQDSFTYLSPCQAGCQSSMPLDNGSSWLYYNCSCIDNTVRGEKAINTILRYNSSTSFDPIFPGPSGFAVSGECGGPCRQIYVFIAIFAALMFVHASGEVGSVLLIIRCTDKMGERAQRSYQVHQPSYGSPAPAAPGYPHTGGSGYLLWGGNSYSKSSASASSSSGSGSVHG |
| Px003613 | PxSPI57 | MKLIILLAFVSLAAVVAGDLVCGSDFCKKNPCVSSTQASSCAVPSVYRANHAGKCACCPACVTYLNEGSGCKTYSKELGETPSAVCKEPLKCLKGVCTKVAPRN |
| Px013712 | PxSPI58 | MKWLLFTAAVGCLAGFANAGALKCTPASSDPVCLEKDIQEESVQSVWQPAVPESQRECAPGSEWVSRCHRCHCSGAGVADCQKLTDCQDAELGLGPVHCKPNSTLRRGCNTCVCLESGLGLCTLNICKEKPVAVPSDVSSPAPPAQGMECLPESTWRSQCNDCKCTTDGRAVCTENKCSGFEKESPKTCAPESAWKNDCNSCWCTEDGRAMCTKMGCITGVIMNFDDDDNDSEAKQEASDKKAEKLDLALRIIQRQCRPDEVFELDCNMCRCNPDGKSFSCTRRACFTEPIIEGVQEDVKDKESKNTSLARKVRAIPPTETPKTCLPGQEFRMDCNKCLCDNKGQDFSCTRINCLATQANSNVGSRRKREVSQQEVAKCSPGEVFDQGCNVCRCTSDGHHATCTLNRCSPKPEEEKKEDESSNLTEGDPNFRCNPGEQFKRGCNDCTCSADGNSVFCSLRYCDKDLTPRL |
| Px015934 | PxSPI59 | MQHRLDEQAASGTWTVSTWLNDGTTASASFLVGDYEVPPFHLSVRHAPRVLRTSERLVWNVCVRYPWSEAVQGMLVIRLRGAARSGGVRTALRLRAPKACHRHAVAARRVGLQGVDPPDVVVADFAFQEDGTRIWQNTTVISQVIEERVALKFLTKYRTVVSPGLPYKLKIKATQWDGKPSANERLRICRSPASQPLVVTEESSFKLHKVLPRNSTVGECDETVTDGRGVARIMFTVGEDEVFPFYRFEATIANGSQISAPPLYLPVKRAGRSHAAFGPFSGDSLTPGARVFVPLYLNLKNVSKAITVHFVVVTRGGTIHRWGATSQCPIANSGRARSASRDTDCNYFRNHDSYGSKPQQTDYFQRENSLYVPDPNAFRRPSRSDPENLLDRHLLRVMLPIRLTHQMCPDSHLLAYFYYNDELVSASKHIEIDECFANKAQLTWKSRQASPGSFTTLQVSTQGPSLCALTALDTASKWVQPEILGVRELLMAEVNKLMDANRNMTVYDATGDCFMTDDNPGLPTNSRDLTIESFASAGLRWVGAETPTQRSCDAPSPLTTGQYGDLKPRSDFQEAWLWKLVGVSGNGSALVSARLPDSITRHEATAYCLSRGGVAVSPPAVLQVFREFFIHADGPKRLRKGDTTFIKYRIFNYLHEPLSVQIHVLPDPHMSLLSLPVVSACLAPRASVAPRAELQARVAGQGRVSVRAVEVRGGCGGNESSSERELVSDEVIIKVNIEHEGVPKQEHRSSLLCARGSSESSPSKVTWSWPGADVVPGSESMVVWAVGDATKTLLSYADSLVTLPRGCGEQNMARLATNLLALTRLEPHTTAAALARDHVSRGLTRQLQYAHPSGGFSAFGVSDGTPSTWLTAFCIRYLRRAYQVISPRSRVPQDVERSERWLLNQQMENGCFRNQGQVFHRELKGGVGEEGEIASVALTAYVITSLVEGGVTIPSKVLANSLACLRALPATKNNVSSRIYAQALLAYTFVTLKKYEEDLRKTNDASLLQDRQETVAGLLKDEESAVMMQLLRSATRQDDFVWWETGKQVPILFLLVQLVNSVV |
| Px013431 | PxSPI60 | MDLVNSYPCTDANHIFLSPGVLAAGGYSRSCLSRFHTAGAATLTLTLAAGGQQTTAVRELVAGDGGCIDIAVPQLPNTKAELIVNVNYPSTPCTWQQRSSVRISSASVLVVSSERARCRRGEALRVRASALHADLTPRNAAIDEIYLQSPADSTGERTVAARWRGVRTRLGLAQIQHRLDEQAASGTWTVTARLNDGTTGLTQMQHWLDEQAAAGTWTASASFLVGDYEVPPFHLSVRHAPRVLRTSERLVWNVCVRYPWSEAVQGMLVIRLRGAARSGGVRTALRLRAPKACHRHAVAARRVGLQGVDPPDVVVADFAFQEDGTRIWQNTTVISQVIEERVALKFLTKYRTVVSPGLPYKLKIKATQWDGKPSANERLRICRTPASQPLVVTEESSFKLHKVLPKNSTVGECDETVTDGRGVARIMFTVGEDEVFPFYRFEATIANDSQISAPPLYLPVKRAGRSHAAFGPLSGDSLTPGARVFVPLYLNLKNVSKAITVHFVVVTRGGTIHRWGATSQCPIANSGRARSASRDTDCNYFRTQDSYGSKPQQTDYFQRENSLYVPDPNTFRRPPRSDPENLLDRHLLRVMLPIRLTHQMCPDSHLLAYFYYNDELVSASKHIEIDECFANKAQLTWKSRQASPGSFTTLQVSTQGPSLCALTALDTASKWVQPEIQGVREVLMAEVNKLMDANRNMTVYDATGDCFMTDDNPGLPTNSRDLTIASLASAGLRWVGAETPMQRSCDAPSPLTTGQYGDVKPRSDFPEAWLWKLVGVSGNGSALVSARLPDSITRHEATAYCLSRGGVAVSPPAVLQVFREFFIHADDPKRLRKGDTTFIKYRIFNYLHEPLSVQIHVLPDPHMSLLSLPLVSACLAPRASVAPRAELQARVAGQGRVSVRAVEVRGGCGGNESSSERELVSDEVIIKVNIEHEGVPKQEHRSSLLCARGSSESSPSKVTWSWPGADVVPGSESMVVWAVGDATKTLLSDADSLVTLPRGCGEQNMARLATNLLALTRLEPHTTAAALARDHVSRGLTRQLQYAHSSGGFSAFGVSDGTPSTWLTAFCIRYLRRAYQVILPRSRVPQDVERSERWLLNQQMENGCFRNQGQVFHRELKGGIGEDGEIASVALTAYVITSLVEGQVTIPSKVLANSLACLRALPATKNNVSSRIYAQALLAYTFVTLKKYEEDLRKTNDASLLQDRQETVAGLLKDEESAVMMQLLRSATKQDDFVWWETGPRSTSMEATSYFILATSRWAGGRAAQWEARAAARWLHSHVGSRGGMTNTQDTLIALEGLTAISSFLPTPAANLTLTVICGATVLSETLTEAAGVPVTLRMKACDEVKVGVEGSGCALVQATRSYNSLSAAPTSGRLTVQLSVRTDGPFDCDNNSTFCFCAAVVEACVLWTGPFPEMALLEVALPGGFGADAARVYALMQDRHTLLRRIEMPPNGGRVTFYLASTDGSAAITGSGGHQCYSLHAVGPRARTKPAYARVMDYYNADVNDTQIYTIPEECPPRYPLEKKPDTGSDNIFNNARSLNGDPIIDEDFFEEPIDAPLEDPLYDNLADDSHMEDKDRSIVLEKYSEPEKAGTEADFPSNKYTDSRHNPEEKILENEIVNRSDAHEFGTFKNGTDPNVASELKSQDVKEVTVETVLENPNLSHFHVIDSDKDLEVPDGDNGPAPSFALPPEGFVVPPPTMSRPSYPPPEDYSIPKMFRPRPHFYYEYPLTRSRRPPAG |
| Px013945 | PxSPI61 | MSYEDTENLLTIVNYPCTDANHIFLSPGVLSAGGYSRACLSRFHTAGAATLTLTLAAGGQQTTAVRELVAGDGGCIDIAVPQLPNTKAELIVNVNYPSTPCTWQQRSSVRISSASVLLIDEIYLQSPADGTGERTVAARWRGVRTRLGLAQIQHRLDEQAASGTWTVTARLNDGTTTQHRADKQAASGTWTVSARLNDGTTASASFLVGDYEVPPFHLSVRHAPRVLRTSERLVWNVCVRYPWSEAVQGMLVIRLRGAARSGGVRTALRLRAPKACHRHAVAARRVGLQGVDPPDVVVADFAFQEDGTRIWQNTTVISQVIEERVSLKFLTKYRTVVSPGLPYKLKIKATQWDGKPSANERLSICRSPASQPSVVTEESSFKLHKVLPRNSTVGECDETVTDGRGVARIMFTVGEDEVFPFYRFEATIANDSQISAPPLYLPVKRAGRSHAAFGPLSGDSLTPGARVFVPLYLNLKNVSKAITVHFVVVTRGGTIHRWGATSQCPIANSGRARSASRDTDCNYFRSHDSYGSKPQQTDYFQRENSLYVPDPNAFRRPHRSDPENLLDRHLLRVMLPIRLTHQMCPDSHLLAYFYYNDELVSASKHIEIDECFANKAQLTWKSRQASPGSYTTLQVSTQGPSLCALTALDTASKWVQPDILGVREVLMAEVTKLMDANRNMTVYDATGDCFMNAFSHPADDNPGLPTNSRDFTIASLASAGLRWVGAETPTQRTCDAPSPLTTGQYGDLKPRSDFQEAWLWKLVGVSGNGSALVSARLPDSITRHEATAYCLSRGGVAVSPPAVLQVFREFFIHADGPKRLRRGDTTFIKYRIFNYLHEPLSVQIHVLPDPHMSLLSLPVVSACLAPRASVAPRAELQARVAGQGRVSVRAVEVRGGCGGNESSSERELVSDEVIIKVDIEHEGVPKQEHRSSLLCARGSSESAPSKVTWSWPGADTVPGSESMVVWAVGDATKTLLSDADSLVTLPRGCGEQNMARMATNLLALSRLEPHTTAAALARDHVSRGFTRQLQYAHPSGGFSAFGISDGTPSTWLTAFCIRYLRRAYQVISPRSRVPLDVERSERWLLNQQMENGCFRNQGQVFHRELKGGVGEEGEIASVALTAYVITSLVEGGVTIPSKVLANSLACLRALPATKSNVSSRIYAQTLLAYTFVTLKKYEEDLRKTNDASLLQDRQETVAGLLKDEESAVMMQLLRSATRQDDFVWWETGSLSTSIEATSYFILATSRWAGGRAAQWEARAAARWLHSHVGSRGGMTNTQDTLIALEALTAISSFLPTPAANLTLTVICGATVLSETLTEAAGVPVTLKMKACDEVKVGVEGSGCALVQATRSYNSLSAAPTSGRLAVQLSVRTDGPFDCDANSTFCFCAAVVEACVLWTGPFPEMALLEVSLPGGFGADAARVYALMQDRHTLLRRIEMPPNGGRVTFYLASTDGSAAITGSGGHQCYSLHAVGPRARTKPAYARVMDYYNADVNDTQIYTIPEECPLRYPLEKKPDTGSDNIFNNARSLNGDPIIDEDFFEEPIDAPLEDPLYDNLADDSHMEDKDRSIVLEKYSEQEKAVTEADFPSNKYTDSRYNSNEKIVENEIVNRSDAHEFGTLKNGTDPNVASELKSQDVKDVTVETVLENPNLSHFHVVDSEKDLEVPDGDNGPAPSFALPPEGFVVPPPTMSRPSYPPPEDYSIPKMFRPRPHFYYEYPLTRSRRPPAG |

**Table S3** RPKM values of the PxSPI genes at different developmental stages obtained from the RNA-seq data

| **Gene ID** | **E** | **L1** | **L2** | **L3** | **L4** | **P** | **AM** | **AF** |
| --- | --- | --- | --- | --- | --- | --- | --- | --- |
| PxSPI1 | 406.76395 | 783.49272 | 1014.1773 | 914.75634 | 922.24786 | 402.9807 | 1364.6795 | 268.36871 |
| PxSPI2 | 25.321914 | 14.20364 | 15.164422 | 12.758647 | 11.239629 | 26.12121 | 30.817689 | 33.231089 |
| PxSPI3 | 17.032331 | 11.04897 | 10.129756 | 10.439768 | 8.3755178 | 13.77394 | 15.993447 | 18.133357 |
| PxSPI4 | 56.941755 | 164.95972 | 94.047803 | 82.895165 | 147.1479 | 252.02072 | 166.66463 | 104.66204 |
| PxSPI5 | 1.083597 | 8.8631007 | 2.8733669 | 3.1494897 | 11.390127 | 8.8741868 | 13.208434 | 2.7846587 |
| PxSPI6 | 109.27884 | 32.970867 | 16.670039 | 15.244632 | 14.403186 | 107.04417 | 56.9171 | 58.079303 |
| PxSPI7 | 26.774442 | 13.68415 | 6.4326691 | 8.0580929 | 3.8607036 | 9.4493331 | 19.630161 | 8.3121015 |
| PxSPI8 | 60.610682 | 0.1563567 | 1.029006 | 1.0741816 | 0.2561391 | 1.7522868 | 0.5132208 | 0.7123128 |
| PxSPI9 | 281.90351 | 273.30518 | 235.63495 | 122.83957 | 1390.9713 | 347.59158 | 36.037779 | 9.4917482 |
| PxSPI10 | 3.3665973 | 0.9792082 | 2.8036935 | 2.9267819 | 8.4944982 | 133.54685 | 7.548325 | 2.4332564 |
| PxSPI11 | 76.005723 | 98.598338 | 55.874697 | 42.442207 | 37.647757 | 172.26916 | 65.971002 | 69.178776 |
| PxSPI12 | 167.40702 | 72.761877 | 90.351948 | 66.217776 | 99.072382 | 306.34399 | 165.21767 | 257.00513 |
| PxSPI13 | 28.811668 | 13.170072 | 11.196509 | 10.450501 | 12.164538 | 26.264803 | 20.234887 | 47.233085 |
| PxSPI14 | 2.6167154 | 0.4428204 | 2.6367161 | 2.6076067 | 2.9016637 | 1.6542269 | 0.3230003 | 2.2863335 |
| PxSPI15 | 12.422921 | 30.633527 | 77.224936 | 75.309704 | 127.42541 | 11.474245 | 5.093251 | 2.5995892 |
| PxSPI16 | 68.455379 | 49.234286 | 25.508712 | 21.243041 | 19.102316 | 97.991974 | 67.210438 | 27.915504 |
| PxSPI17 | - | 0.1420759 | - | 0.1394388 | - | 0.1447493 | 229.28672 | - |
| PxSPI18 | 23.474602 | 18.452868 | 18.635248 | 8.7906468 | 15.08052 | 50.063158 | 19.826765 | 33.512882 |
| PxSPI19 | 23.474602 | 18.452868 | 18.635248 | 8.7906468 | 15.08052 | 50.063158 | 19.826765 | 33.512882 |
| PxSPI20 | 2.7754065 | 0.5870942 | 1.6558935 | 0.576197 | 0.8014676 | 3.1900869 | 31.689455 | 4.1010836 |
| PxSPI21 | - | - | - | 0.2731209 | - | - | 13.092624 | - |
| PxSPI22 | - | - | 0.6003825 | 0.6267406 | - | - | - | - |
| PxSPI23 | 35.364555 | 55.747499 | 28.903547 | 18.505785 | 114.82648 | 20.463437 | 5.3818833 | 0.3734829 |
| PxSPI24 | 11.030462 | 2.3333229 | 7.6779679 | 3.8166893 | 4.140916 | 13.074755 | 9.7862879 | 7.7952593 |
| PxSPI25 | 28.457455 | 11.570395 | 11.172065 | 10.741816 | 13.575374 | 20.071648 | 27.713925 | 20.229684 |
| PxSPI26 | 9.1213435 | 24.350319 | 24.215129 | 23.898347 | 62.554784 | 14.263369 | 39.145152 | 15.590519 |
| PxSPI27 | - | 0.2856233 | 0.2685326 | 0.1121287 | 0.04679 | 0.0581996 | 0.3750088 | 141.51979 |
| PxSPI28 | 1.9824332 | 28.484939 | 33.17628 | 38.931404 | 165.58575 | 11.013395 | 29.024876 | 8.5757649 |
| PxSPI29 | - | - | - | - | 80.002496 | - | - | - |
| PxSPI30 | 0.7405427 | 2.9051514 | 6.0517424 | 6.5410529 | 612.43564 | 8.5312358 | 1.7451115 | 1.4013501 |
| PxSPI31 | - | 0.6999969 | 0.2193705 | 0.9160054 | 0.1911192 | 0.2377228 | 0.7658834 | 0.212598 |
| PxSPI32 | 0.3038051 | 0.514122 | - | - | 0.8422202 | 2.3570822 | 2.5313095 | 1.8737449 |
| PxSPI33 | - | 1.5456407 | 1.2715106 | 1.7065706 | 0.1582516 | - | 0.4227806 | 0.1760365 |
| PxSPI34 | - | 0.3721865 | 3.4991615 | 1.0958347 | 7.9261704 | 1.5167591 | 0.4072182 | 7.4604935 |
| PxSPI35 | 2.3584869 | 23.947262 | 21.763864 | 21.465864 | 17.91491 | 56.847033 | 25.240561 | 15.928064 |
| PxSPI36 | 5.346505 | - | - | - | - | 0.2363592 | 75.514444 | - |
| PxSPI38 | 3.40173 | - | 0.8326472 | 1.5211039 | - | 0.9023056 | 547.24326 | 0.4034706 |
| PxSPI39 | - | - | - | - | - | 0.7186969 | 332.65463 | 0.321369 |
| PxSPI40 | 1.6051792 | 2.7164058 | 1.2769328 | 0.444331 | 0.3708283 | 3.2287726 | 5.4488222 | 0.8250071 |
| PxSPI41 | 1.7760913 | 12.971694 | 11.972424 | 11.644137 | 9.3292083 | 23.93345 | 17.567721 | 7.4229125 |
| PxSPI42 | 19.104483 | 11.347215 | 7.7677486 | 5.3298052 | 7.9962515 | 93.497684 | 18.380135 | 8.394325 |
| PxSPI43 | 3.6326988 | 0.1617771 | 2.2814533 | 2.0640656 | 4.1077886 | 44.666533 | 11.682275 | 5.7486494 |
| PxSPI44 | 3.0827158 | 5.7637283 | 3.7971484 | 4.2115923 | 2.1365059 | 48.263337 | 13.441024 | 7.5898363 |
| PxSPI45 | 5.3011462 | 0.8971001 | 1.1597036 | 0.6603366 | 0.7348021 | 77.459849 | 7.6069257 | 2.349974 |
| PxSPI46 | 1.3640524 | 3.4625291 | 1.7633111 | 1.3451447 | 1.5953113 | 2.3517882 | 5.3669514 | 2.8919392 |
| PxSPI47 | 19.554001 | 15.833751 | 10.621135 | 9.6032828 | 7.2132085 | 55.735892 | 30.657796 | 38.579364 |
| PxSPI48 | 8.2728464 | 133.93274 | 110.56274 | 122.28673 | 60.775905 | 29.953075 | 110.28721 | 48.472339 |
| PxSPI49 | 1.2689912 | 0.5368708 | - | 0.5269058 | - | 9.8455114 | - | - |
| PxSPI50 | 6.8283812 | 8.3957959 | 6.7900396 | 3.8984755 | 13.753757 | 15.819888 | 5.3338308 | 8.9658136 |
| PxSPI51 | 0.3658061 | 91.309117 | 70.713413 | 43.743933 | 62.367265 | 32.480699 | 37.929464 | 28.765808 |
| PxSPI52 | 3.0245777 | 7.644819 | 3.5782664 | 2.1252911 | 0.9137338 | 16.914448 | 8.2207856 | 3.9461129 |
| PxSPI53 | 0.4537848 | 0.5759468 | 0.9024736 | 0.9420942 | 0.15725 | 1.1735683 | 0.6301572 | 0.8746119 |
| PxSPI54 | 5.255351 | 223.9257 | 183.05378 | 139.34313 | 280.19475 | 96.756921 | 152.90936 | 41.094856 |
| PxSPI55 | - | 0.4428204 | 1.2489708 | - | 1.4508319 | 1.3534584 | 626.45908 | 0.4034706 |
| PxSPI56 | 2.4976763 | 3.6051752 | 2.5713101 | 2.4401784 | 5.1931159 | 9.8791368 | 2.4483158 | 3.1715436 |
| PxSPI57 | 10.242572 | 15.59993 | 299.84815 | 246.10013 | 1522.9106 | 82.410577 | 448.19983 | 72.647767 |
| PxSPI58 | 97.728487 | 14.683586 | 27.488848 | 51.576357 | 40.723403 | 61.151798 | 61.585047 | 139.77302 |
| PxSPI59 | 2.0215602 | 0.0570173 | 0.2680279 | 0.111918 | 0.4670206 | 2.9045081 | 0.9357598 | 4.9353102 |
| PxSPI60 | 13.31711 | 0.3837437 | 0.9183538 | 0.2739062 | 2.2859571 | 13.07954 | 3.7024304 | 22.440762 |
| PxSPI61 | 12.24011 | 0.2457551 | 0.2640571 | 0.0344562 | 2.5880725 | 4.5783654 | 1.6517316 | 13.722904 |

E, eggs; L1, 1^st^-instar larvae; L2, 2^nd^-instar larvae; L3, 3^rd^-instar larvae; L4, 4^th^-instar larvae; P, pupae; AM, adult males; AF, adult females. -, missing values.

**Table S4** RPKM values of the PxSPI genes at different tissues obtained from the RNA-seq data

| Gene ID | L4M | L4H | AMH | AFH |
| --- | --- | --- | --- | --- |
| PxSPI1 | 3765.707 | 1588.318 | 410.4159 | 485.1577 |
| PxSPI2 | 2.129952 | 23.80103 | 17.13045 | 24.85528 |
| PxSPI3 | 1.011968 | 30.98132 | 12.03996 | 15.79285 |
| PxSPI4 | 9.675036 | 328.6498 | 362.5929 | 366.9059 |
| PxSPI5 | 0.150223 | 29.52605 | 7.31165 | 12.73577 |
| PxSPI6 | 1.440547 | 141.1712 | 194.342 | 193.3998 |
| PxSPI7 | 0.448411 | 12.7671 | 73.49616 | 96.83628 |
| PxSPI8 | - | 0.282345 | 3.261058 | 8.946788 |
| PxSPI9 | 44.42779 | 1118.737 | 26.50118 | 52.02457 |
| PxSPI10 | 0.262533 | 10.97107 | 21.84266 | 16.44378 |
| PxSPI11 | 4.858546 | 314.3704 | 94.36244 | 103.0524 |
| PxSPI12 | 7.710294 | 360.2179 | 324.264 | 326.7765 |
| PxSPI13 | 2.066004 | 19.98717 | 10.31347 | 8.365547 |
| PxSPI14 | 1.305956 | 8.26289 | 0.543276 | 0.183611 |
| PxSPI15 | 0.442861 | 23.86236 | 1.105379 | 3.735852 |
| PxSPI16 | 1.947996 | 162.1229 | 169.2415 | 174.4399 |
| PxSPI18 | 3.179641 | 93.1137 | 23.65647 | 27.28678 |
| PxSPI19 | 3.179641 | 93.1137 | 23.65647 | 27.28678 |
| PxSPI20 | 0.192383 | 0.53008 | 2.881117 | 3.894927 |
| PxSPI22 | - | 0.576579 | - | - |
| PxSPI23 | 4.432603 | 144.3394 | 4.023181 | 6.11872 |
| PxSPI24 | 0.382299 | 11.58701 | 4.293972 | 7.739919 |
| PxSPI25 | 1.229663 | 110.397 | 23.01923 | 33.8422 |
| PxSPI26 | 8.52821 | 34.46393 | 27.08506 | 33.00629 |
| PxSPI27 | - | 0.154732 | - | 0.071059 |
| PxSPI28 | 0.213759 | 24.17615 | 9.603723 | 6.723386 |
| PxSPI30 | - | 10.44067 | 2.23636 | 1.34631 |
| PxSPI31 | - | 0.210673 | 0.286265 | - |
| PxSPI32 | 0.505412 | 0.928389 | 5.046024 | 4.47669 |
| PxSPI33 | - | 2.267753 | - | - |
| PxSPI34 | - | 2.688342 | - | - |
| PxSPI35 | 0.078472 | 1.585591 | 7.442885 | 9.830206 |
| PxSPI36 | - | - | 0.142311 | 0.288582 |
| PxSPI38 | - | - | 0.814914 | - |
| PxSPI40 | 0.445065 | 0.817537 | 16.10774 | 9.573818 |
| PxSPI41 | 0.311023 | 0.928389 | 4.269712 | 3.050053 |
| PxSPI42 | 3.759874 | 47.86847 | 13.35105 | 16.42812 |
| PxSPI43 | 0.318073 | 17.23586 | 123.4527 | 122.7551 |
| PxSPI44 | 0.454941 | 8.850601 | 46.14382 | 33.85946 |
| PxSPI45 | 0.330713 | 16.70586 | 15.40856 | 9.903806 |
| PxSPI46 | 0.850968 | 3.191407 | 28.05463 | 23.68912 |
| PxSPI47 | 7.607864 | 26.18276 | 38.19662 | 35.07647 |
| PxSPI48 | 13.99215 | 197.8219 | 228.4393 | 297.2129 |
| PxSPI49 | - | 2.908405 | 1.317325 | 0.667825 |
| PxSPI50 | 4.792392 | 11.24843 | 3.98726 | 5.053407 |
| PxSPI51 | 100.1077 | 226.6454 | 24.68304 | 21.5612 |
| PxSPI52 | 0.064509 | 3.3179 | 16.3832 | 19.79456 |
| PxSPI53 | - | 0.866693 | 5.41729 | 3.582162 |
| PxSPI54 | 222.3179 | 628.6216 | 70.14237 | 72.6985 |
| PxSPI55 | 2.611912 | 7.996345 | 1.086553 | 0.550834 |
| PxSPI56 | 2.933049 | 8.979503 | 1.220145 | 4.175274 |
| PxSPI57 | 9.655782 | 17.73666 | 167.2877 | 140.1478 |
| PxSPI58 | 1.139592 | 32.09743 | 282.0711 | 264.3651 |
| PxSPI59 | - | 0.669243 | 0.979327 | 1.9859 |
| PxSPI60 | 0.137179 | 1.165425 | 6.120371 | 5.641373 |
| PxSPI61 | 0.138052 | 0.41208 | 3.919575 | 3.319028 |

L4M, midguts of 4th-instar larvae; L4H, heads of 4th-instar larvae; AMH, heads of adult males; AFH, heads of adult females. -, missing values.

**Table S5** Primers used for qPCR study on gene expression

| Gene ID | Gene Name | Forward primer (5'-3') | Reverse primer (5'-3') | Product Length (bp) |
| --- | --- | --- | --- | --- |
| Px001494 | PxSPI1 | AAACAAGCCAGCCGCATC | TCCACCCAGTCGTTGATTTCT | 149 |
| Px004227 | PxSPI2 | ACACCGCCAATGAGGATACG | GCCCTGTCTTTGAACTCGGA | 180 |
| Px007271 | PxSPI4 | CCACTAACCATGTGGATTGCTTT | GGGCTCCTGATTGCGTCTT | 89 |
| Px007273 | PxSPI5 | TGCCTTGATCCTCCCACTCA | TTGAAGTCCCTTTCGAGCGT | 177 |
| Px002424 | PxSPI6 | TGCCATCAATAAGGCTCTACCA | TCTCATCAATCACCAACGCTTC | 137 |
| Px007274 | PxSPI7 | TTGTTGGTCGACGCGATGTA | ACGTTTCCCGTTTCGTCTGT | 76 |
| Px010990 | PxSPI9 | GGACAAGAAGGTCAAGAAGAGCA | AGCAACGATCATGCGGAAC | 127 |
| Px015089 | PxSPI11 | TCATGCAGAGGACGGAGGT | CGGTCAGGTTTGAGTCTTTGG | 126 |
| Px015087 | PxSPI12 | GTTGTTGTTATGCCGTTTGAGG | GCTGTGTGTCTGTTCGTATTGTG | 184 |
| Px003823 | PxSPI15 | ACAATGCTCGGTGGAAAAGG | AGACACAGCCGCCAGAAGA | 148 |
| Px003939 | PxSPI35 | ACGCGCTTCAAGCTGATAGT | TTCCACATGACAGCCACCTC | 173 |
| Px013945 | PxSPI61 | GACCAACAGCCGAGACTTCA | GACCAACAGCCGAGACTTCA | 165 |
