# Supplementary material for: Characterization and expression profiling of serine protease inhibitors in the diamondback moth, Plutella xylostella (Lepidoptera: Plutellidae)
Source: BMC Genomics. 2017 Feb 14;18:162. doi: 10.1186/s12864-017-3583-z (PMC5309989; doi:10.1186/s12864-017-3583-z)
Supplement: Additional file 2: Figure S1. — Multiple sequence alignment of 25 P. xylostella serpins with known serpins from M. sexta (MsSRPN3-7), D. melanogaster (DmSpn27A) and A. gambiae (AgSRPN9) using Clustal X. Figure S2. Alignment of P. xylostella serpins with some known serpins from other insect species by Clustal X2. A, Alignment of PxSPI1 with MsSRPN1J and BmSPI1; B, Alignment of PxSPI3, 18 and 19 with MsSRPN3a, 3b, BmSPI3 and AgSRPN1, 2, 3; C, Alignment of PxSPI4 with MsSRPN4a, MsSRPN4b and BmSPI4; D, Alignment of PxSPIs 5, 16 and 22 with MsSRPN5A, 5B and BmSPI5. E, Alignment of PxSPI7 with MsSRPN7 and BmSPI7. F, Alignment of PxSPI6 with MsSRPN6, BmSPI6, AgSRPN9, DmSpn88Ea and DmSpn88Eb. Figure S3. Alignment of serine protease inhibitor domains using Clustal X2 with default parameters and shading was done using GeneDoc. (A) TIL family, (B) Kunitz family, (C) WAP family, (D) Kazal family, (E) amfpi family, (F) Antistasin family, (G) Pacifastin family. Figure S4. Multiple sequence alignment of P. xylostella α2Ms with other α2Ms using Clustal X2. Figure S5. Phylogenetic tree of α2Ms, complement proteins and thioester-containing proteins constructed using the neighbor joining method. Figure S6. qPCR-based expression profiling of PxSPI genes across different developmental stages. Figure S7. Expression profiling of the P. xylostella SPI genes in different tissues. (DOCX 8635 kb) [file 12864_2017_3583_MOESM2_ESM.docx]

**Supplemental Figures**

**Additional file 2: Fig. S1**

Multiple sequence alignment of 25 *P. xylostella* serpins with known serpins from *M. sexta* (MsSRPN3-7), *D. melanogaster* (DmSpn27A) and *A. gambiae* (AgSRPN9) using Clustal X.

Conserved residues involving structural conformation of serpins are highlighted, residues highlighted in blue corresponding to the shutter region, in yellow to the breach region, and in violet to the gate region. The RCL domain is shown in green rectangle. Predicted helix and β-strand secondary structures of serpins are assigned based on the structure of AgSRPN9 (Suwanchaichinda & Kanost, 2009).

**Additional file 2: Fig. S2**

Alignment of *P. xylostella* serpins with some known serpins from other insect species by Clustal X2.

**A**, Alignment of PxSPI1 with MsSRPN1J and BmSPI1; **B**, Alignment of PxSPI3, 18 and 19 with MsSRPN3a, 3b, BmSPI3 and AgSRPN1, 2, 3; **C**, Alignment of PxSPI4 with MsSRPN4a, MsSRPN4b and BmSPI4; **D**, Alignment of PxSPIs 5, 16 and 22 with MsSRPN5A, 5B and BmSPI5. **E**, Alignment of PxSPI7 with MsSRPN7 and BmSPI7. **F,** Alignment of PxSPI6 with MsSRPN6, BmSPI6, AgSRPN9, DmSpn88Ea and DmSpn88Eb; Black shaded sequence indicates identical sequence across all the aligned serpins, gray shaded sequence indicates conserved amino acid substitutions, and light gray shaded sequences indicates semi-conserved amino acid substitutions. The hinge region and predicted P1 position are boxed in purple and red, respectively.

**Additional file 2: Fig. S3**

Alignment of serine protease inhibitor domains using Clustal X2 with default parameters and shading was done using GeneDoc.

Black shaded sequence indicates identical residues across all the aligned sequences, gray shaded sequence means conserved amino acid substitutions, and light gray shaded sequences indicates semi-conserved amino acid substitutions. The predicted P1 positions of SPIs are marked with asterisks. Conserved Cys residues are marked as C under the sequences. (A) TIL family, (B) Kunitz family, (C) WAP family, (D) Kazal family, (E) amfpi family, (F) Antistasin family, (G) Pacifastin family.

**Additional file 2: Fig. S4**

Multiple sequence alignment of *P. xylostella* α2Ms with other α2Ms using Clustal X2.

The α2Ms of *P. xylostella* were aligned with α2Ms of *Apis mellifera* (GenBank: XP_392454.3), *Eriocheir sinensis* (ADD71943), *Hasarius adansoni* (AB622470), *Harpegnathos saltator* (EFN79621), *Ixodes ricinus* (ACJ26770), *Litopenaeus vannamei* (ABI79454), *Nasonia vitripennis* (XP_001604193.2), and *Tribolium castaneum* (EFA07508.1). The bait region, thioester site and receptor-binding domain are marked with a purple box, a red box and a green box, respectively. The conserved FPETW sequence is marked with a pink box in the bait region. The GFIPLKPTVK sequence in the mammalian domain that is replaced by other residues, is underlined, and the conserved GGxxxTQDT is marked with black underline in the receptor-binding domain.

**Additional file 2: Fig. S5**

Phylogenetic tree of α2Ms, complement proteins and thioester-containing proteins constructed using the neighbor joining method.

The sequences used for phylogenetic analysis were obtained from GenBank (NCBI) with accession numbers: PxSPI59 (*P. xylostella*, Px015934), PxSPI60 (*P. xylostella*, Px013431), PxSPI61 (*P. xylostella*, Px013945), TcA2M (*Tribolium castaneum*, EFA07508.1), BmSPI75 (*B. mori*, XP_012546509), NvA2M (*Nasonia vitripennis*, XP_001604193.2), AmA2M (*Apis mellifera*, XP_392454.3), OmA2M (*Ornithodoros moubata*, AAN10129), IrA2M (*Ixodes ricinus*, ACJ26770), HaA2M (*Hasarius adansoni*, AB622470), MrA2M (*Macrobrachium rosenbergii*, ABK60046), FcA2M (*Fenneropenaeus chinensis*, ABP97431), LvA2M (*Litopenaeus vannamei*, ABI79454), EsA2M (*Eriocheir sinensis*, ADD71943), CcA2M (*Cyprinus carpio*, AB026128), LcA2M (*Lethenteron camtschaticum*, D13567), HsA2M (*Harpegnathos saltator*, EFN79621), HsTEP (*H. saltator*, EFN86807), AmTEP (*A. mellifera*, XP_001122599), NvTEP (*N. vitripennis*, XP_001599750), IsTEP (*Ixodes scapularis*, XP_002409560), TcTEP (*T. castaneum*, XP_972838), ApTEP (*Acyrthosiphon pisum*, XP_001944348.2), DpTEP (*Daphnia pulex*, EFX86067), AgTEP1 (*Anopheles gambiae*, AAG00600), AaTEP (*Aedes aegypti*, XP_001653325), BbC3 (*Branchiostoma belcheri*, AB050668), GgC3 (*Gallus gallus*, U16848), MmC3 (*Mus musculus*, K02782), and SpC3 (*Strongylocentrotus purpuratus*, AF025526).

**Additional file 2: Fig. S6**

qPCR-based expression profiling of PxSPI genes across different developmental stages. E: eggs; L1: 1^st^-instar larvae; L2: 2^nd^-instar larvae; L3: 3^rd^-instar larvae; L4: 4^th^-instar larvae; P: pupae; A: adults. X axis: developmental stage; Y axis: relative expression value.

**Additional file 2: Fig. S7**

Expression profiling of the *P. xylostella* SPI genes in different tissues.

The log2 RPKM values are presented by bar colors where red represents higher expression values, green represents lower expression values, and the gray represents missed values. L4M: midguts of 4^th^-instar larvae; L4H: heads of 4^th^-instar larvae; AMH: heads of adult males; AFH: heads of adult females.

Fig. S1


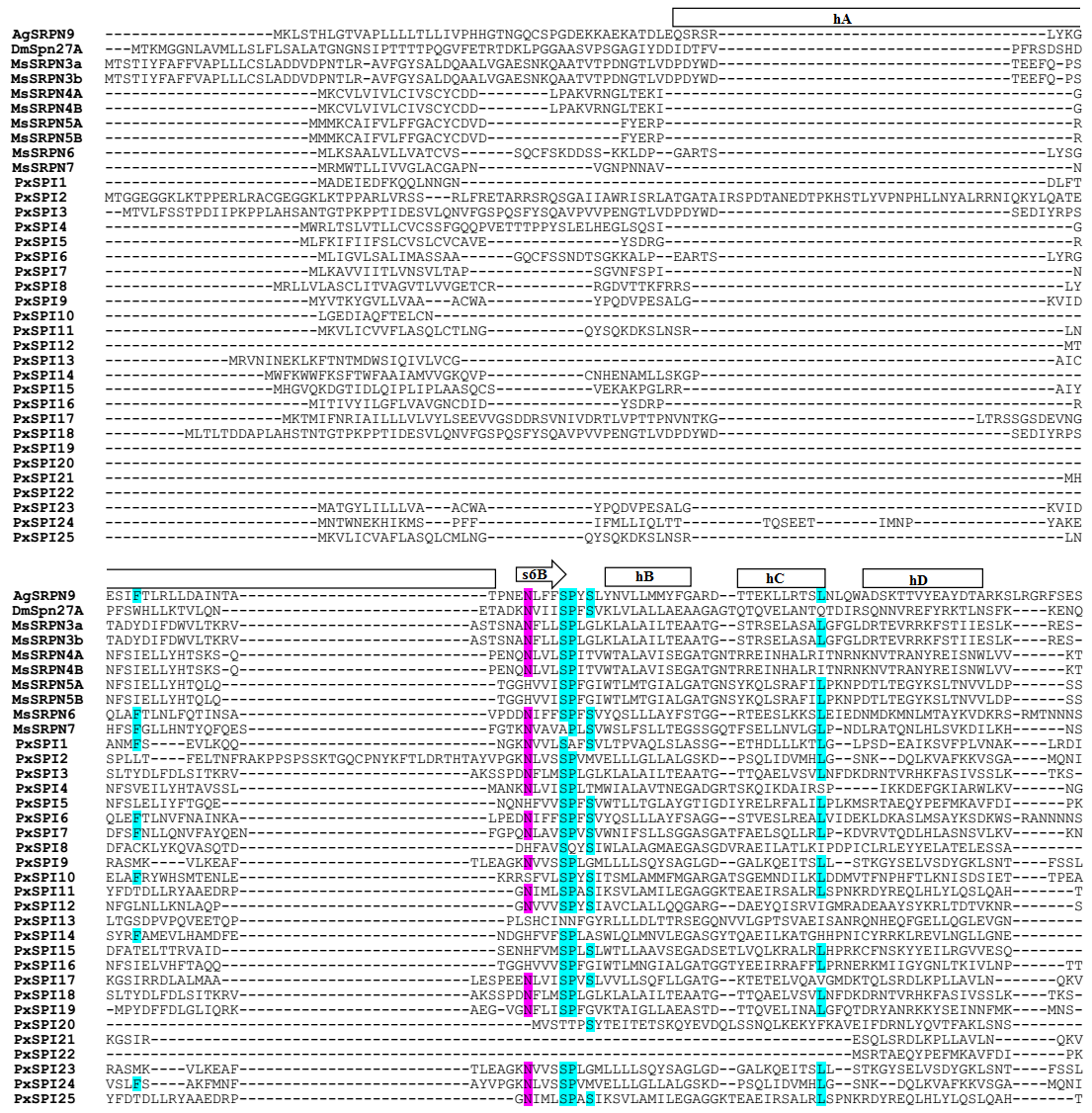

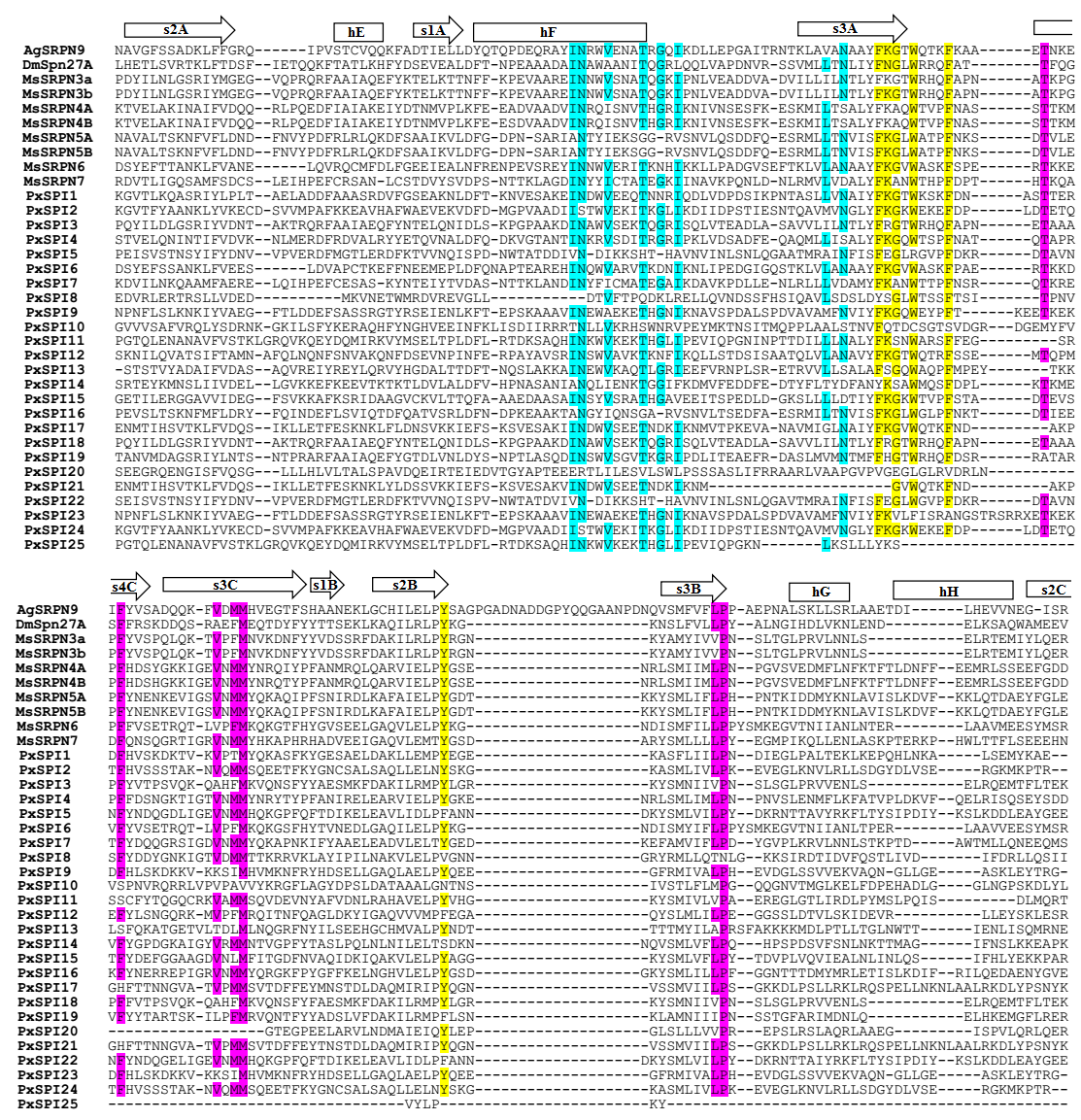

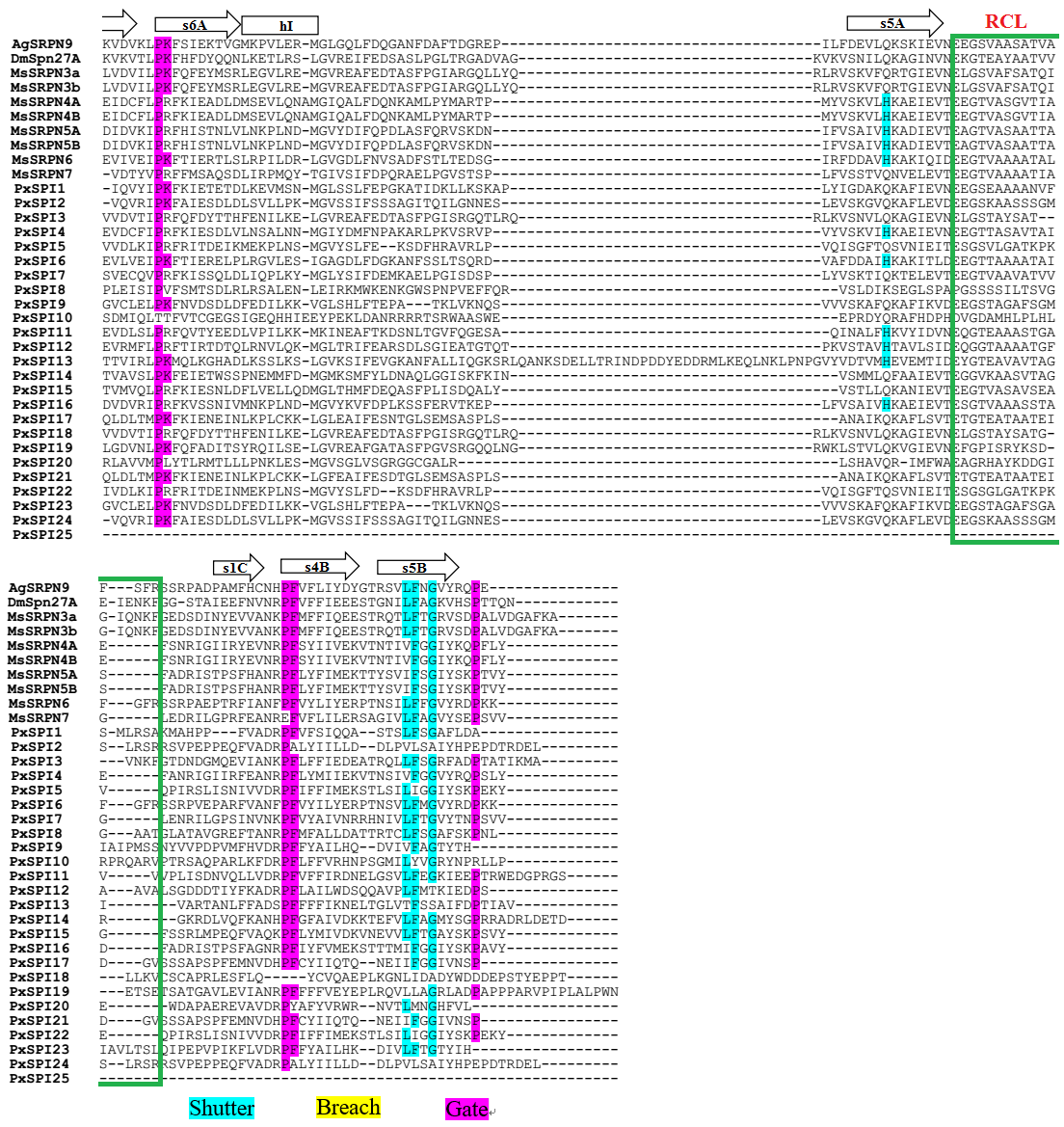


**Fig. S1** Multiple sequence alignment of 25 *P. xylostella* serpins with known serpins from *M. sexta* (MsSRPN3-7), *D. melanogaster* (DmSpn27A) and *A. gambiae* (AgSRPN9) using Clustal X.

Conserved residues involving structural conformation of serpins are highlighted, residues highlighted in blue corresponding to the shutter region, in yellow to the breach region, and in violet to the gate region. The RCL domain is shown in green rectangle. Predicted helix and β-strand secondary structures of serpins are assigned based on the structure of AgSRPN9 (Suwanchaichinda & Kanost, 2009).

Fig. S2


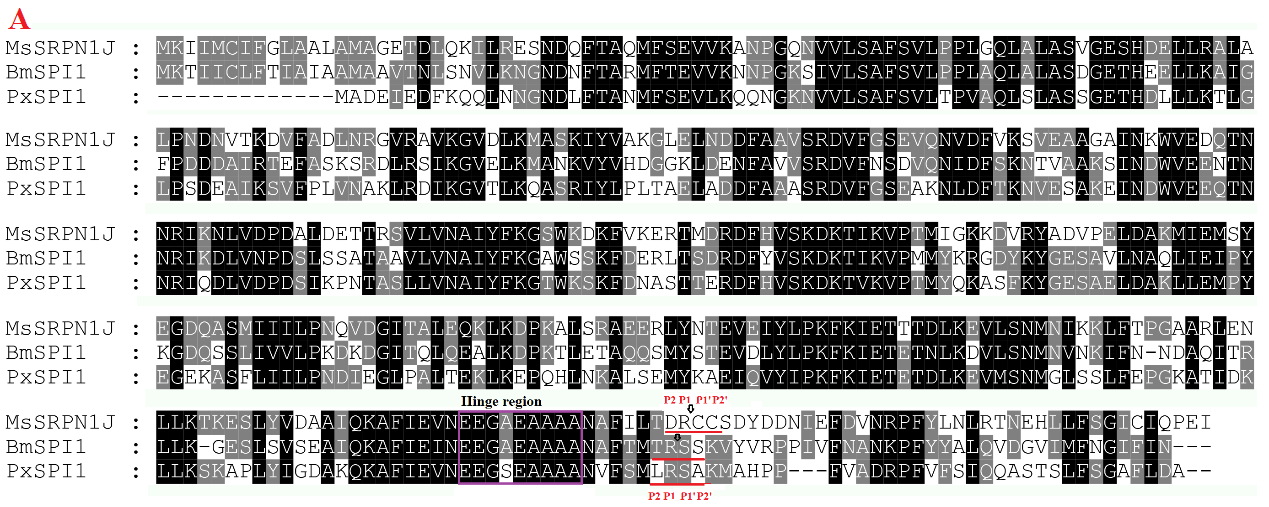

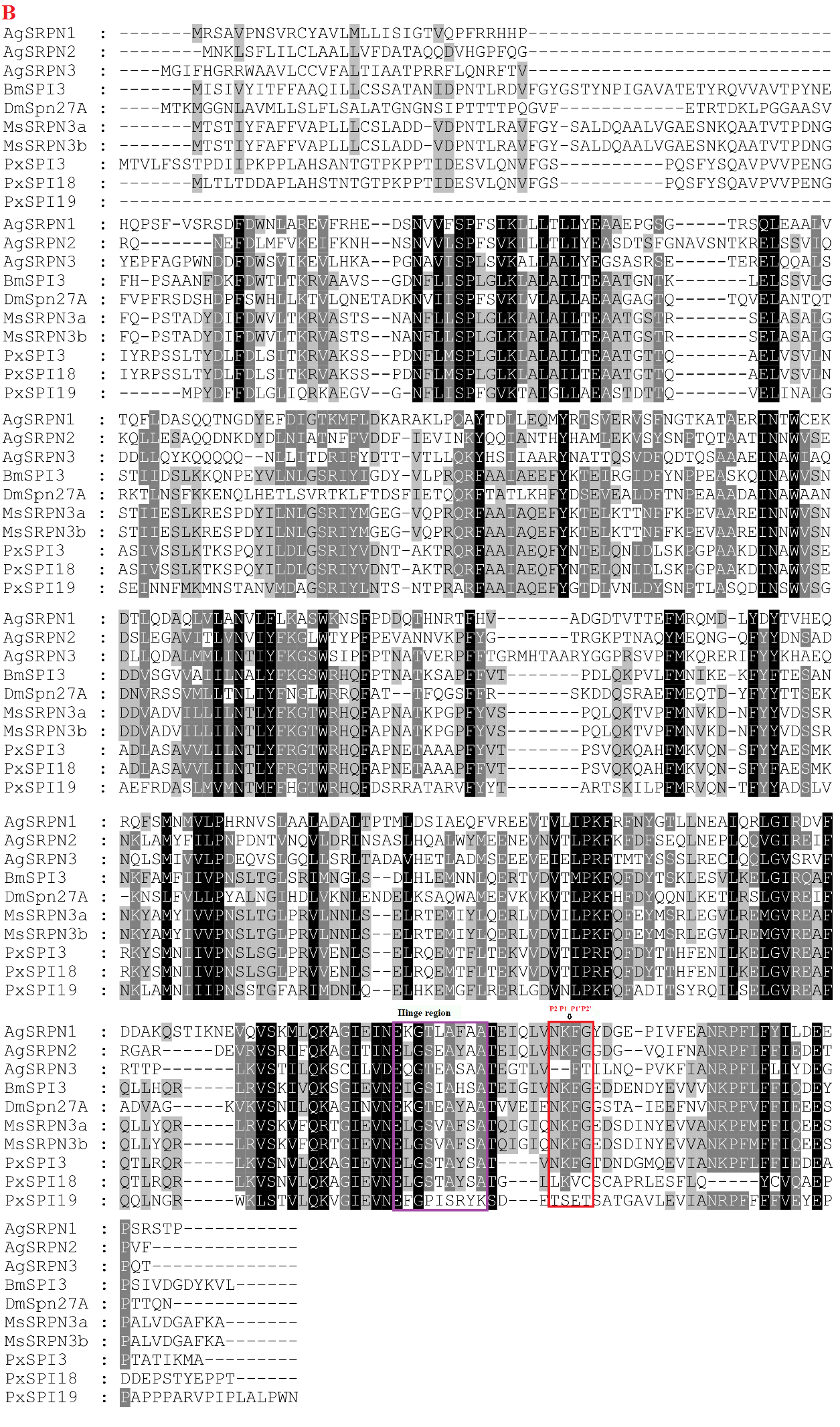

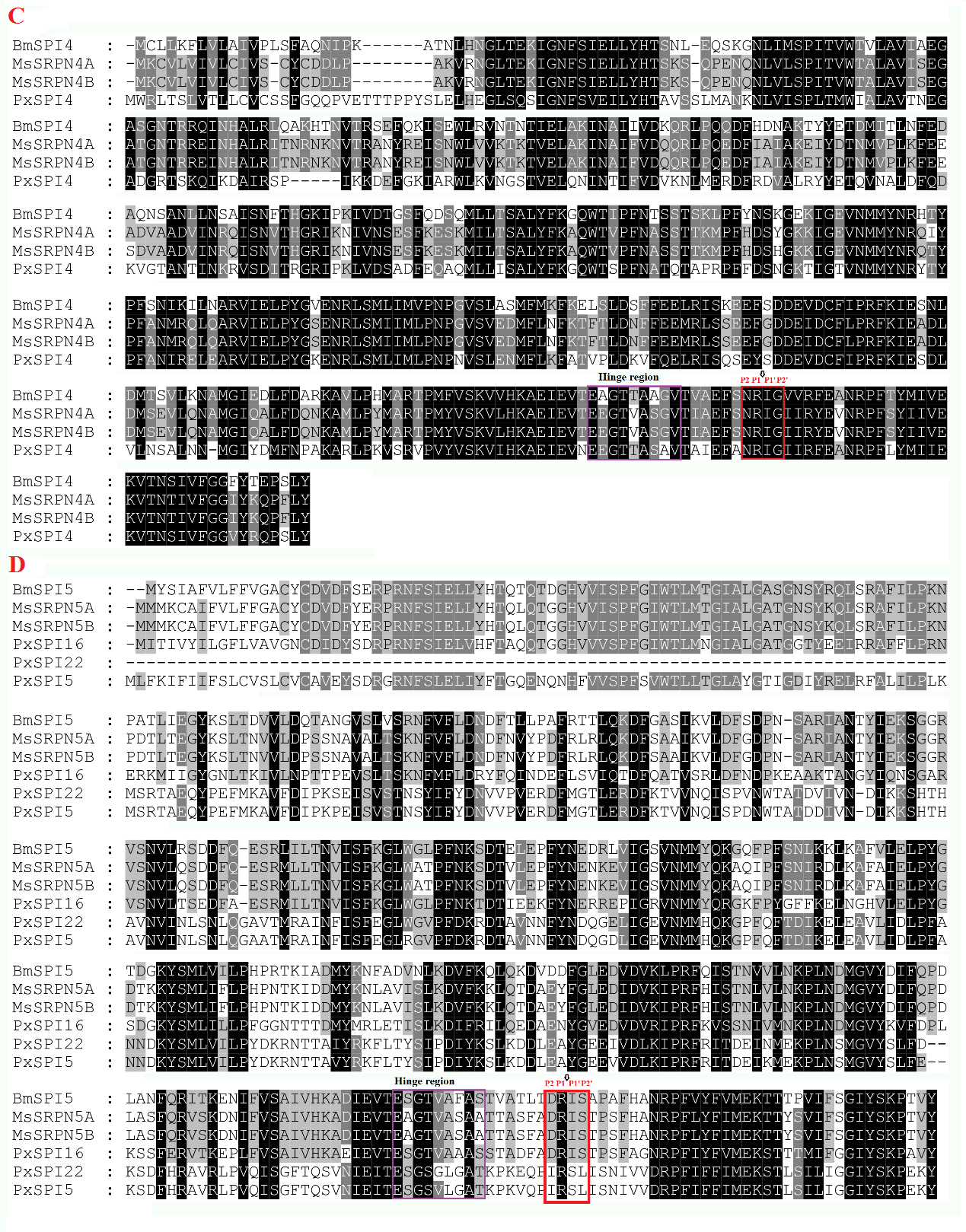

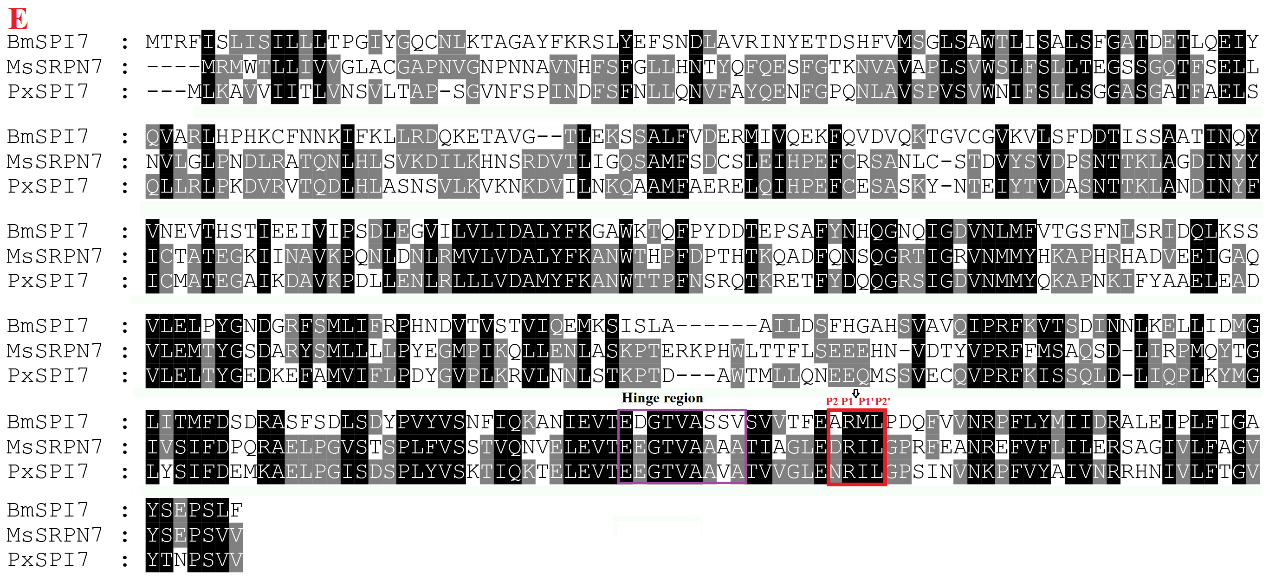

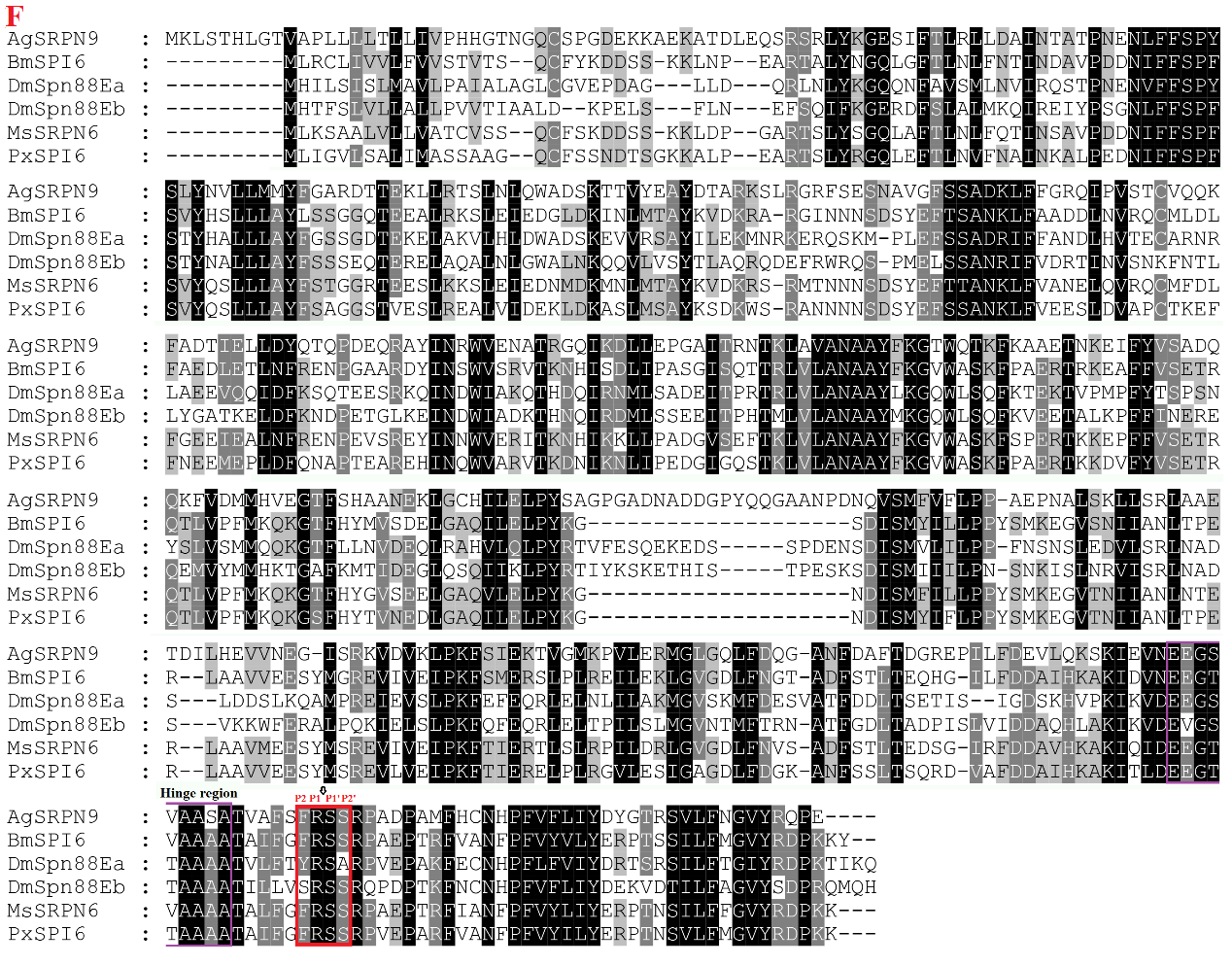


**Fig. S2** Alignment of *P. xylostella* serpins with some known serpins from other insect species by Clustal X2.

**A**, Alignment of PxSPI1 with MsSRPN1J and BmSPI1; **B**, Alignment of PxSPI3, 18 and 19 with MsSRPN3a, 3b, BmSPI3 and AgSRPN1, 2, 3; **C**, Alignment of PxSPI4 with MsSRPN4a, MsSRPN4b and BmSPI4; **D**, Alignment of PxSPIs 5, 16 and 22 with MsSRPN5A, 5B and BmSPI5. **E**, Alignment of PxSPI7 with MsSRPN7 and BmSPI7. **F,** Alignment of PxSPI6 with MsSRPN6, BmSPI6, AgSRPN9, DmSpn88Ea and DmSpn88Eb; Black shaded sequence indicates identical sequence across all the aligned serpins, gray shaded sequence indicates conserved amino acid substitutions, and light gray shaded sequences indicates semi-conserved amino acid substitutions. The hinge region and predicted P1 position are boxed in purple and red, respectively.

Fig. S3


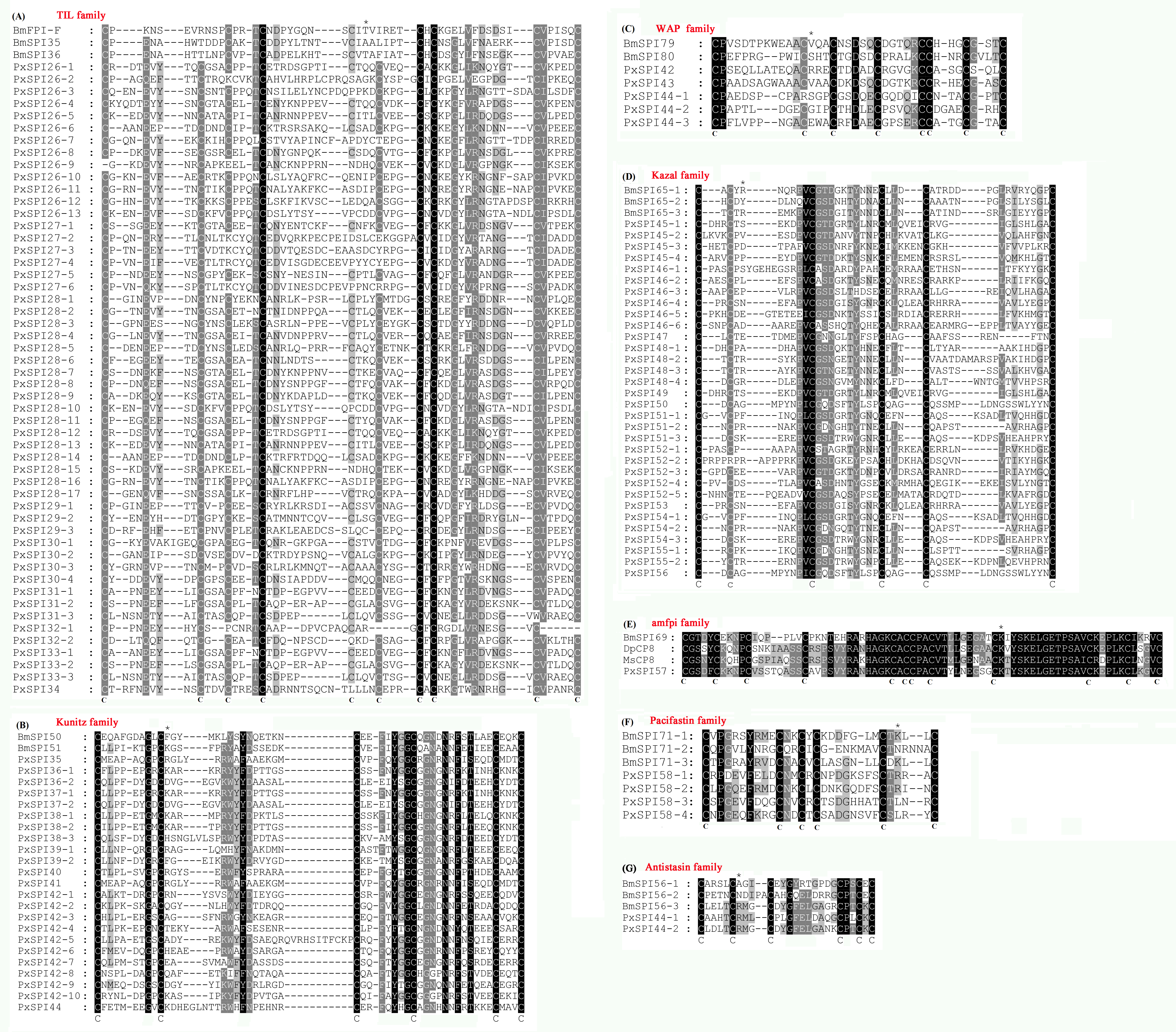


**Fig. S3** Alignment of serine protease inhibitor domains using Clustal X2 with default parameters and shading was done using GeneDoc.

Black shaded sequence indicates identical residues across all the aligned sequences, gray shaded sequence means conserved amino acid substitutions, and light gray shaded sequences indicates semi-conserved amino acid substitutions. The predicted P1 positions of SPIs are marked with asterisks. Conserved Cys residues are marked as C under the sequences. (A) TIL family, (B) Kunitz family, (C) WAP family, (D) Kazal family, (E) amfpi family, (F) Antistasin family, (G) Pacifastin family.

Fig. S4


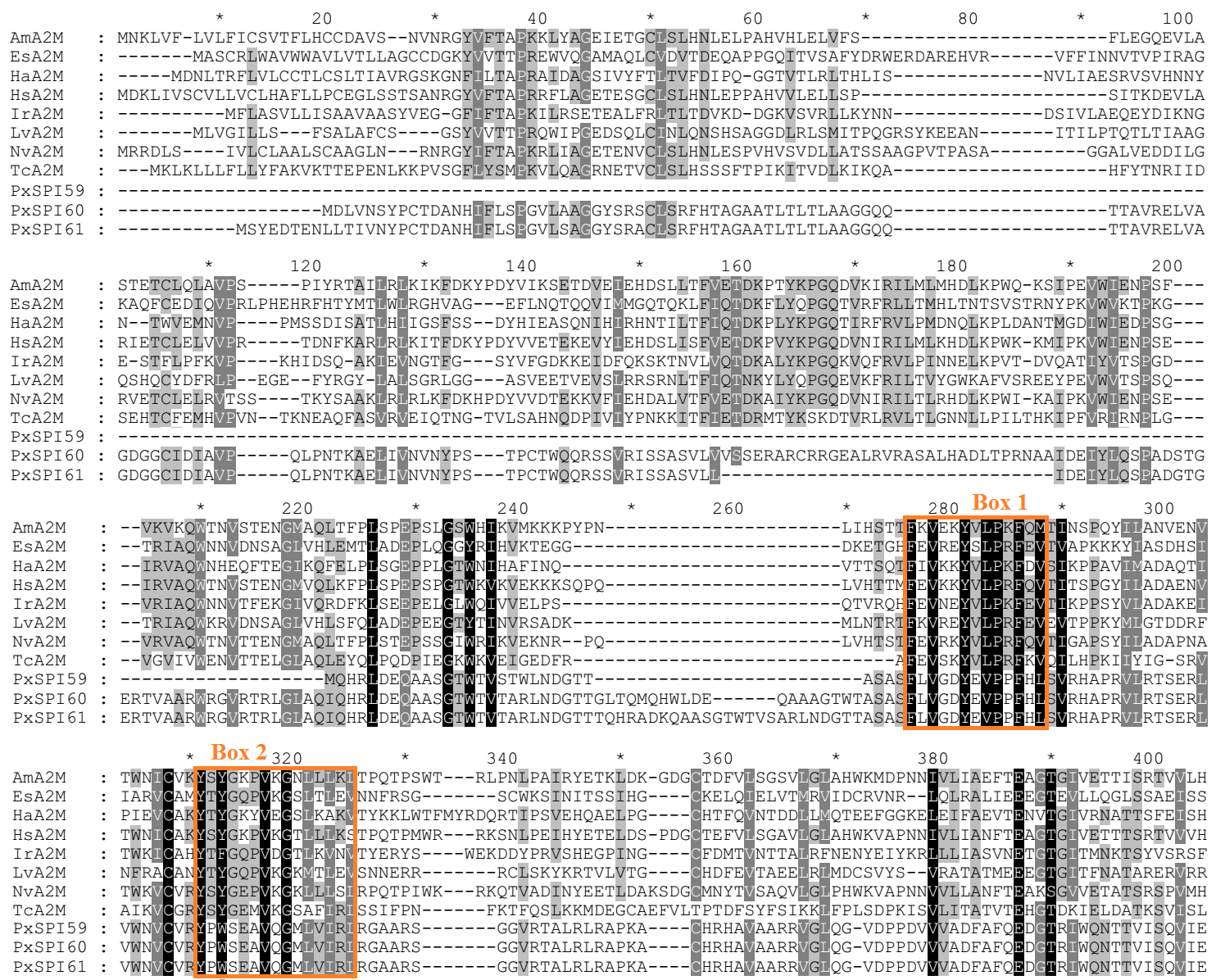

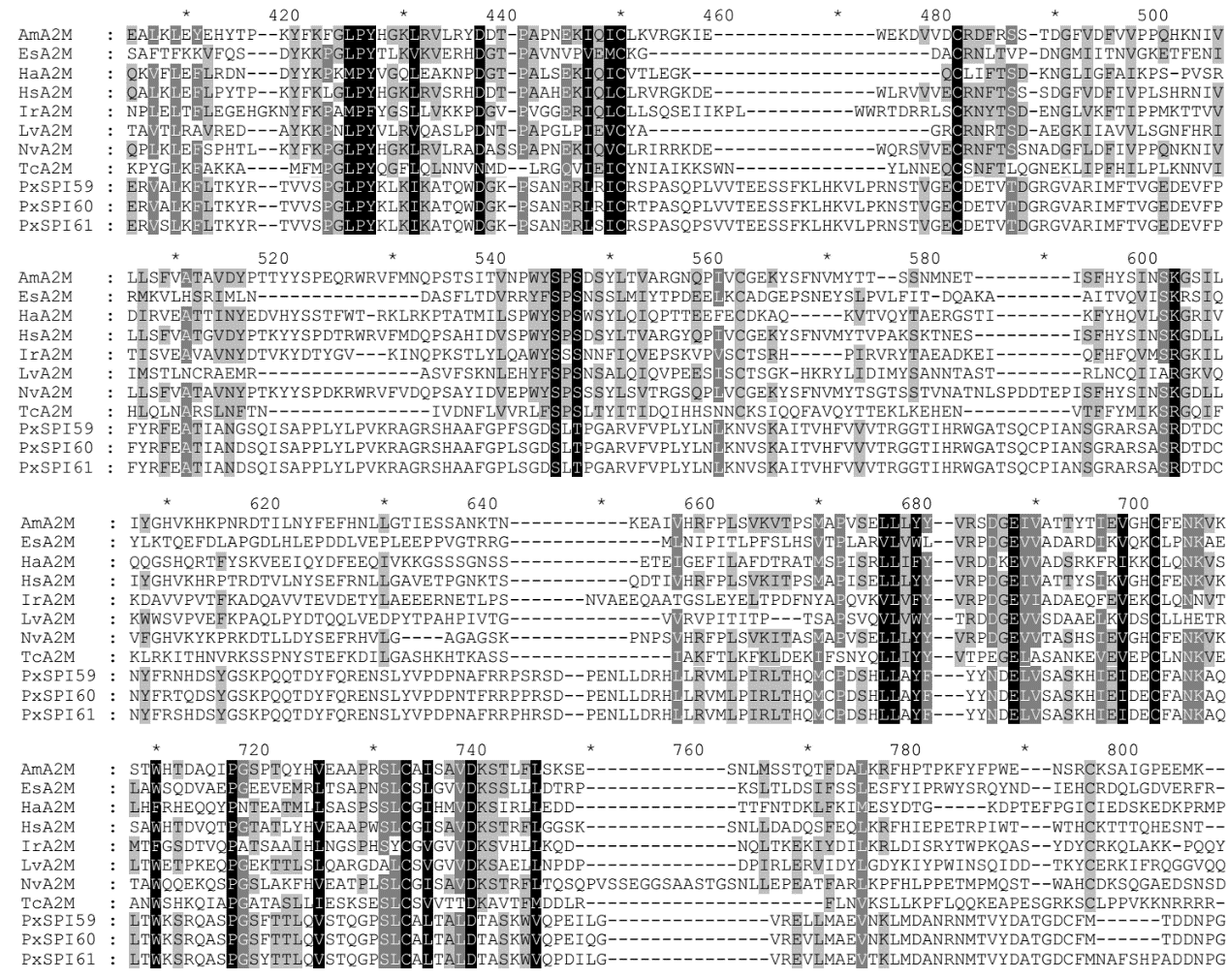

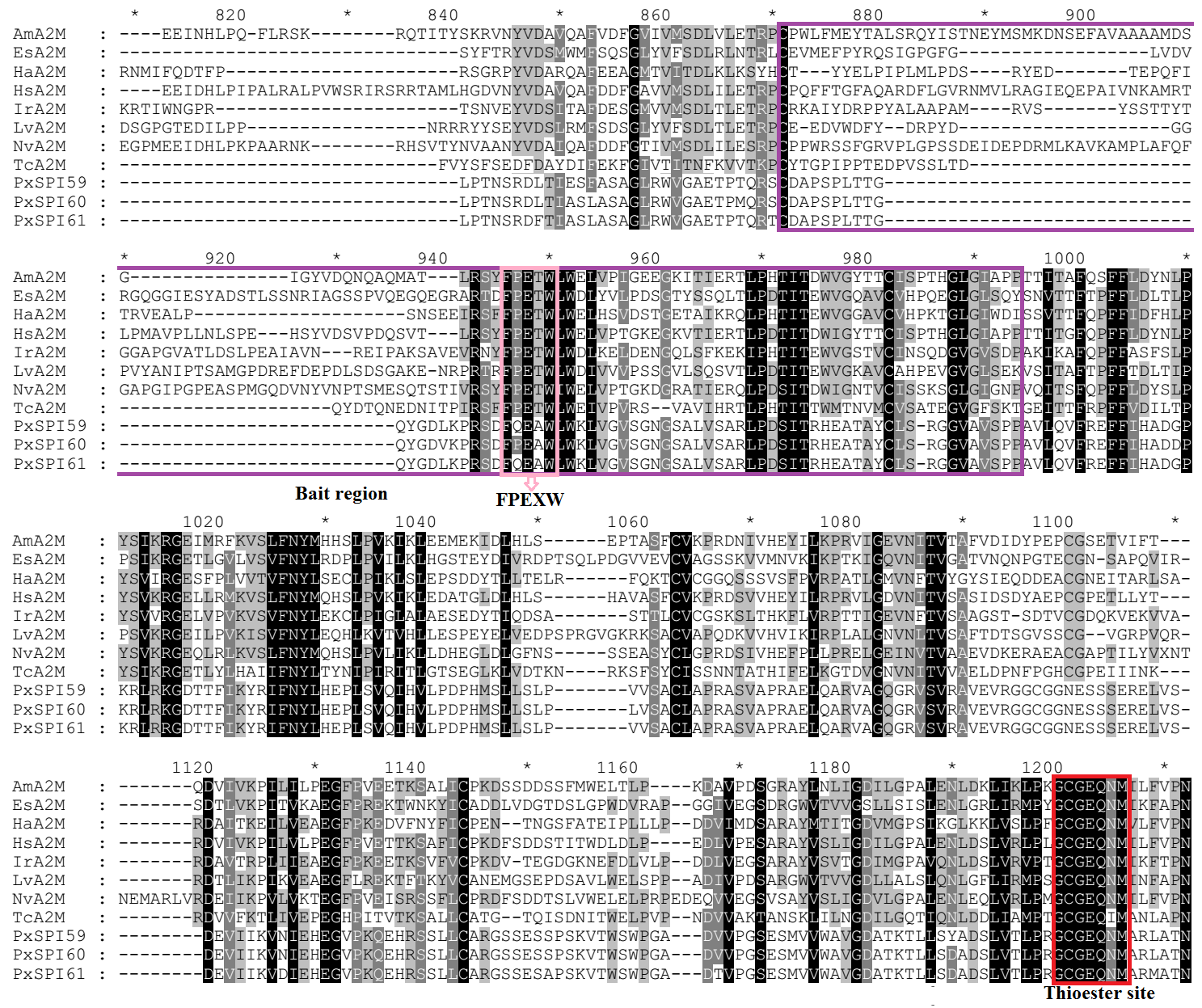

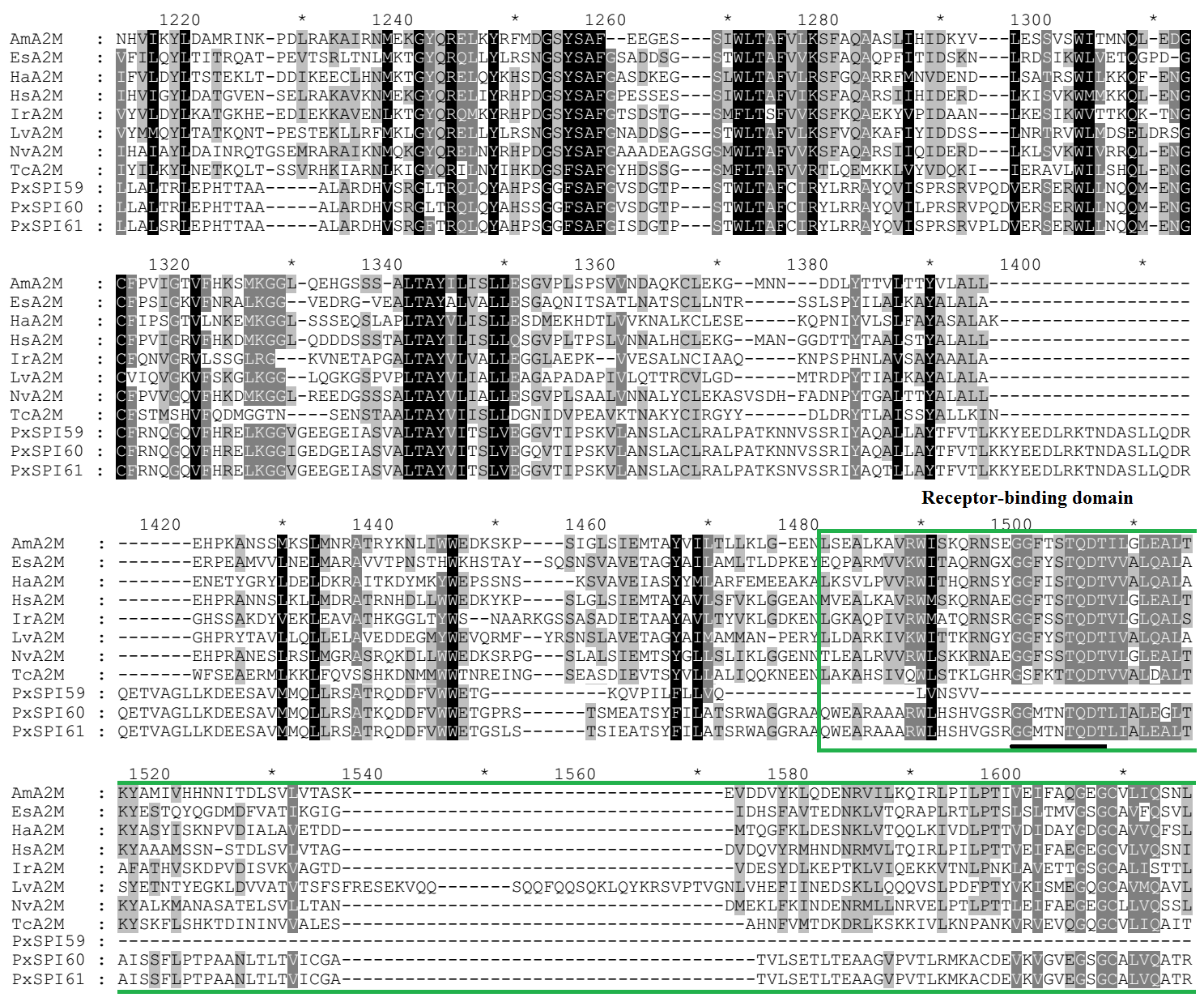

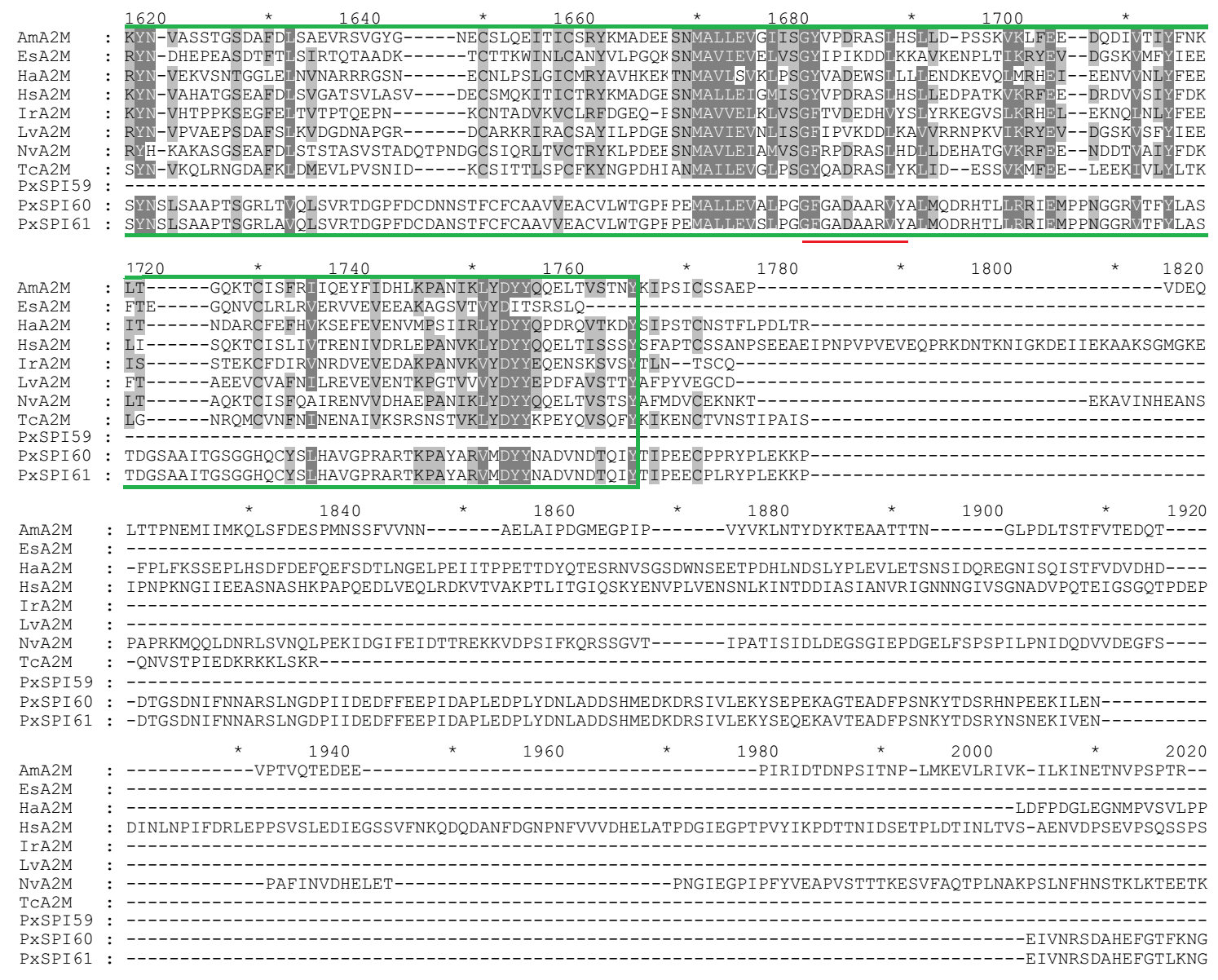

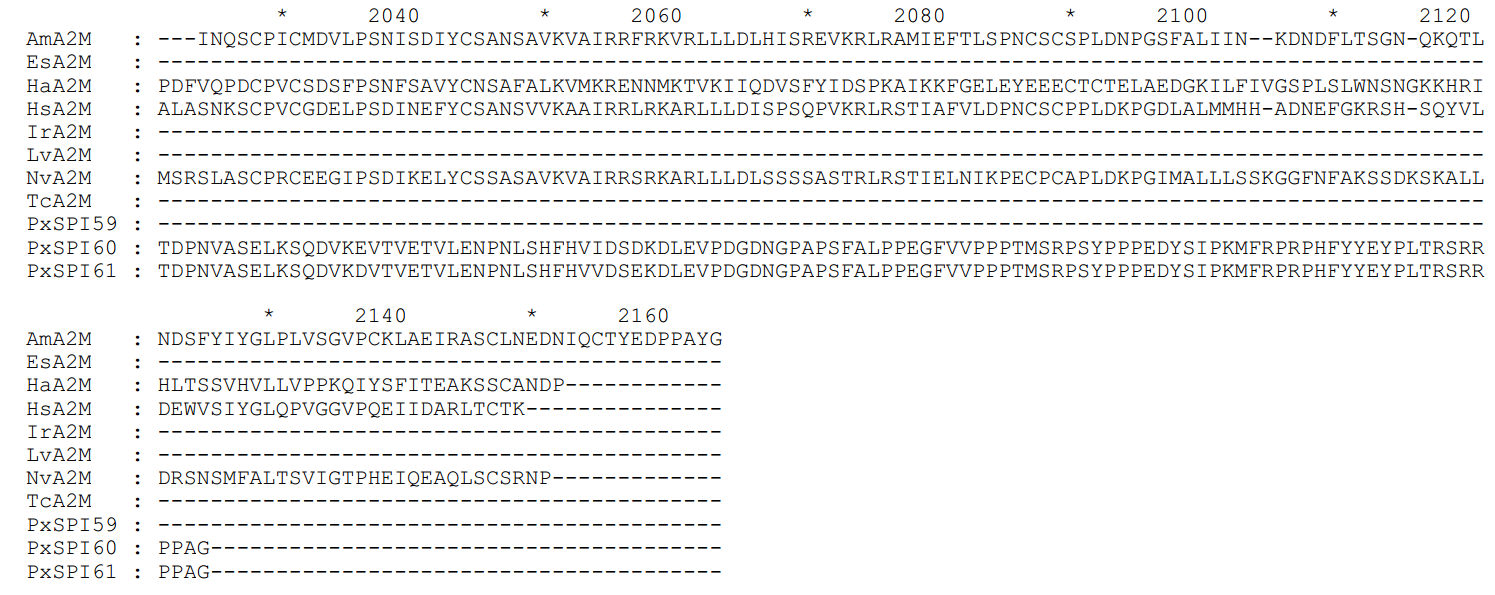


**Fig. S4** Multiple sequence alignment of *P. xylostella* α2Ms with other α2Ms using Clustal X2.

The α2Ms of *P. xylostella* were aligned with α2Ms of *Apis mellifera* (GenBank: XP_392454.3), *Eriocheir sinensis* (ADD71943), *Hasarius adansoni* (AB622470), *Harpegnathos saltator* (EFN79621), *Ixodes ricinus* (ACJ26770), *Litopenaeus vannamei* (ABI79454), *Nasonia vitripennis* (XP_001604193.2), and *Tribolium castaneum* (EFA07508.1). The bait region, thioester site and receptor-binding domain are marked with a purple box, a red box and a green box, respectively. The conserved FPETW sequence is marked with a pink box in the bait region. The GFIPLKPTVK sequence in the mammalian domain that is replaced by other residues, is underlined, and the conserved GGxxxTQDT is marked with black underline in the receptor-binding domain.

Fig. S5

**Fig. S5** Phylogenetic tree of α2Ms, complement proteins and thioester-containing proteins constructed using the neighbor joining method.


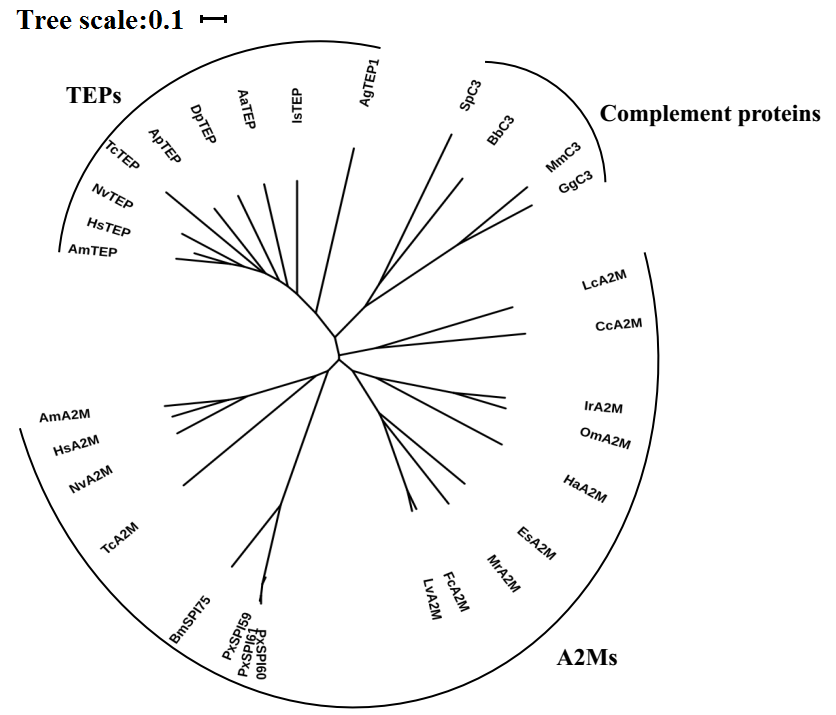


The sequences used for phylogenetic analysis were obtained from GenBank (NCBI) with accession numbers: PxSPI59 (*P. xylostella*, Px015934), PxSPI60 (*P. xylostella*, Px013431), PxSPI61 (*P. xylostella*, Px013945), TcA2M (*Tribolium castaneum*, EFA07508.1), BmSPI75 (*B. mori*, XP_012546509), NvA2M (*Nasonia vitripennis*, XP_001604193.2), AmA2M (*Apis mellifera*, XP_392454.3), OmA2M (*Ornithodoros moubata*, AAN10129), IrA2M (*Ixodes ricinus*, ACJ26770), HaA2M (*Hasarius adansoni*, AB622470), MrA2M (*Macrobrachium rosenbergii*, ABK60046), FcA2M (*Fenneropenaeus chinensis*, ABP97431), LvA2M (*Litopenaeus vannamei*, ABI79454), EsA2M (*Eriocheir sinensis*, ADD71943), CcA2M (*Cyprinus carpio*, AB026128), LcA2M (*Lethenteron camtschaticum*, D13567), HsA2M (*Harpegnathos saltator*, EFN79621), HsTEP (*H. saltator*, EFN86807), AmTEP (*A. mellifera*, XP_001122599), NvTEP (*N. vitripennis*, XP_001599750), IsTEP (*Ixodes scapularis*, XP_002409560), TcTEP (*T. castaneum*, XP_972838), ApTEP (*Acyrthosiphon pisum*, XP_001944348.2), DpTEP (*Daphnia pulex*, EFX86067), AgTEP1 (*Anopheles gambiae*, AAG00600), AaTEP (*Aedes aegypti*, XP_001653325), BbC3 (*Branchiostoma belcheri*, AB050668), GgC3 (*Gallus gallus*, U16848), MmC3 (*Mus musculus*, K02782), and SpC3 (*Strongylocentrotus purpuratus*, AF025526).

Fig. S6


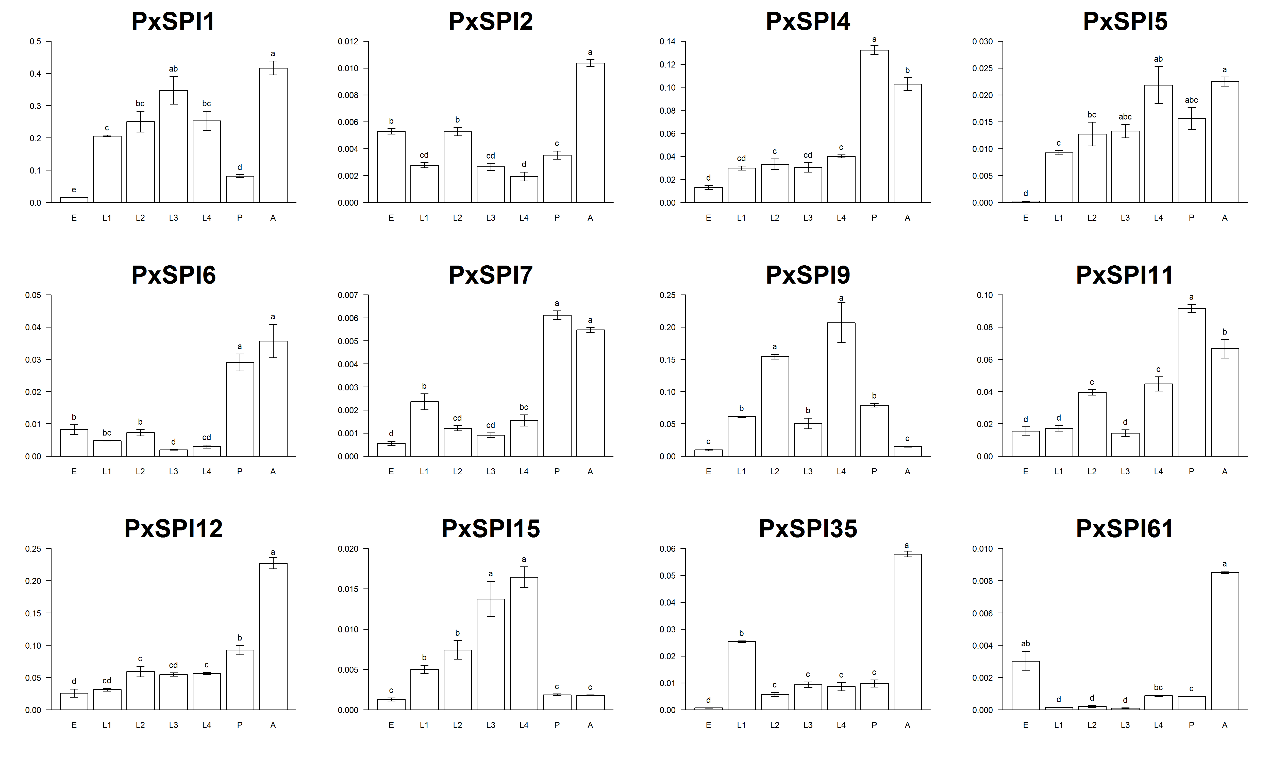


**Fig. S6** qPCR-based expression profiling of PxSPI genes across different developmental stages. E: eggs; L1: 1^st^-instar larvae; L2: 2^nd^-instar larvae; L3: 3^rd^-instar larvae; L4: 4^th^-instar larvae; P: pupae; A: adults. X axis: developmental stage; Y axis: relative expression value.

Fig. S7


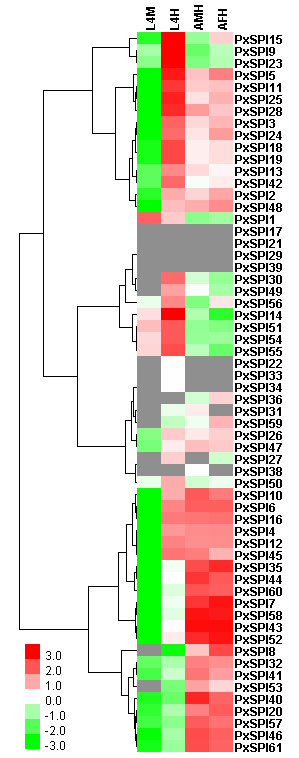


**Fig. S7** Expression profiling of the *P. xylostella* SPI genes in different tissues.

The log2 RPKM values are presented by bar colors where red represents higher expression values, green represents lower expression values, and the gray represents missed values. L4M: midguts of 4^th^-instar larvae; L4H: heads of 4^th^-instar larvae; AMH: heads of adult males; AFH: heads of adult females.
